# Novel niclosamide-derived Schiff bases as a dual-targeted anticancer agents

**DOI:** 10.1038/s41598-025-33185-2

**Published:** 2026-01-13

**Authors:** Magda M. F. Ismail, Tamer M. Nasr, Moustafa S. Abusaif, Abeer S. Abdelmoniem, Yousry A. Ammar

**Affiliations:** 1 Department of Pharmaceutical Medicinal Chemistry and Drug Design, Faculty of Pharmacy (Girls), Al-Azhar University, Cairo, 11754 Egypt; 2 Department of Medicinal Chemistry, Faculty of Pharmacy, Egypt Japan University of Science and Technology (E-JUST), P.O. 21934, Alexandria, Egypt; 3 Department of Pharmaceutical Chemistry, Faculty of Pharmacy, Helwan University, Ain-Helwan, Cairo, 11795 Egypt; 4 Department of Chemistry, Faculty of Science, Al-Azhar University, Cairo, 11754 Egypt; 5 Pharmaceutical Chemistry Department, Faculty of Pharmacy, MTI University, Cairo, Egypt

**Keywords:** Synthesis, Cytotoxicity, JAK1, CDK7, Apoptosis, Caspases, MD, In silico, Biochemistry, Cancer, Drug discovery, Oncology

## Abstract

Nicosamide (NIC), an approved anthelmintic medication, has demonstrated encouraging antitumor action. To enhance NIC’s pharmacokinetic and pharmacodynamic characteristics and make it a potential anticancer drug, thirteen NIC-Schiff bases were created by condensation reaction of NIC-amine **2** with various monocyclic/bicyclic/tricyclic aromatic aldehydes/acetophenone in absolute ethanol. Several spectroscopic methods, such as elemental analysis, IR, ¹H NMR, and MS, were used to determine the structures of these novel synthesized compounds. MTT assay was used to assess the target compounds’ activity against prostate cancer cell line (PC-3) and two breast cancer cell lines (MCF-7) and (MDA-MB-231). IC_50_ was determined for the most promising compounds using doxorubicin and NIC as reference standards. Among the compounds examined, the noteworthy compound **11** exhibited IC_50_ values of 2.85, 4.61, and 7.69 µM against MCF-7, MDA-MB-231, and PC-3, respectively, whereas, compound **8** displayed IC_50_ values of 8.70 and 8.20 µM against MCF-7 and MDA-MB-231, respectively. Using NIC as a reference standard, a mechanistic analysis of the interesting compounds **8** and **11** revealed dual inhibitory effect on JAK1 and CDK7 enzymes. They elicited greater amounts of JAK1 inhibition in MCF-7 and MDA-MB-231 (81-85.5%, 53.6-69.18%) than NIC (75.6% and 36.6%, respectively). Surprisingly, compound **11** inhibits CDK7 more than NIC does on MCF-7 (80.7%) and PC-3 (83.4%) cells. Additionally, compound **11** produced cell cycle arrest at G2/M (35.03%) with overexpression of pre-G (20.68%) in contrast to the control (20.95%) and (0.31%), respectively. With respect to the control, compound **11** performed better than NIC in raising the levels of apoptosis mediators, caspases 1, 3, and 9, in MDA-MB-231 cells by 4.60, 3.03, and 2.69 times, respectively. Like NIC, it also activates caspase 1 in PC-3 cells 3.5 times more than control. According to flow cytometry results, **11** significantly increased apoptotic cell death from 1.34% to 10.92% and necrotic cell death from 1.05% to 3.47% in comparison to control MCF-7 cells. The docking results of NIC-Schiff bases **8** and **11** confirmed the combined in vitro inhibitory effects of the JAK1 and CDK7 enzymes. Compound **11** establishes a more stable, tightly bound complex with JAK1, as indicated by molecular dynamics.

**Supplementary Information:**

The online version contains supplementary material available at 10.1038/s41598-025-33185-2.

## Introduction

 Niclosamide (NIC) is an anthelmintic drug authorized by the FDA in the early 1950s. It mostly acts locally in the intestinal lumen due to its restricted absorption from the human intestines^1^. Later, it was found that NIC had anticancer activity in vitro; with minimal effect on non-tumor cells^2^. NIC was shown to be an effective strategy for reducing cell proliferation in breast^3^ and prostate cancers^4,5^ causing apoptosis, and limiting metastasis. This was achieved by blocking number of carcinogenic signaling pathways such as Wnt, catenin^6^, mTORC1^7^, NF-κ^8^, CDK4/6^9^, and JAK\STAT3^10^. However, NIC’s therapeutic potential as a systemic cancer treatment has been hampered by its poor pharmacokinetic characteristics, due to its poor solubility, absorption, and metabolic instability^11^.

**Fig. 1:**
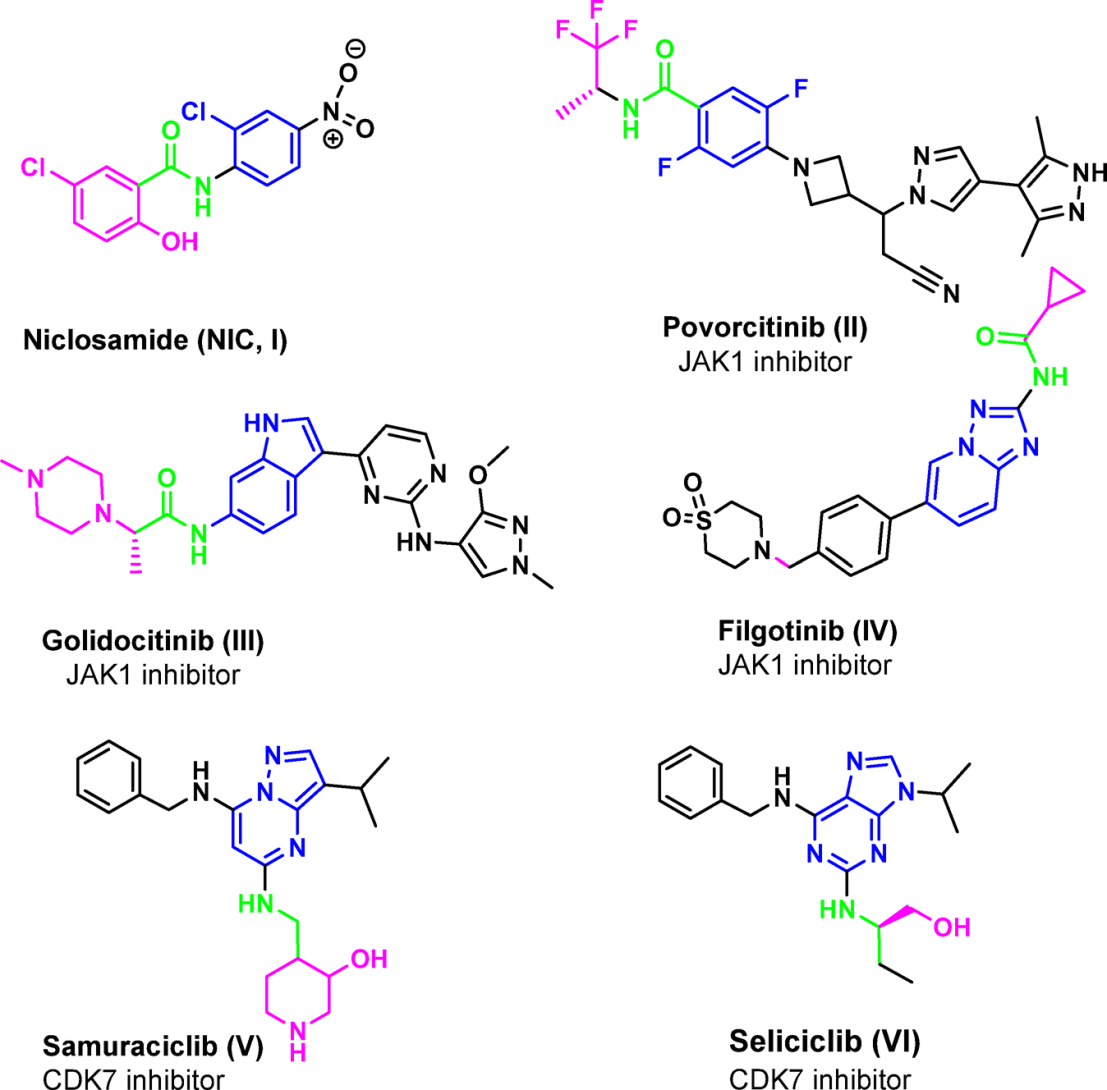
Some examples of JAK1 and CDK7 inhibitors.

A family of intracellular, non-receptor tyrosine kinases known as Janus kinase (JAK) uses the JAK-STAT pathway to transduce cytokine-mediated signals^10^. Four members of the JAK family (JAK1, JAK2, JAK3, and TY2K) are required for the transmission of cytokine-mediated signals *via* the JAK-signal transducers and activators of transcription (STAT) pathway^11^. The primary kinase that phosphorylates STAT3, so initiating downstream signaling, is often JAK1^12^. NIC **(I)**, povorcitinib **(II)**, golidocitinib **(III)** and filgotinib **(IV)** are some examples of JAK1 inhibitors used in the treatment of some cancers, inflammatory diseases such as rheumatoid arthritis and various skin conditions^13^, (Fig. 1). Collectively, these findings suggest that JAK1 inhibition by small molecules could be a viable therapeutic approach for addressing these resistance or “escape” mechanisms. According to reports^14^, in mutant cells, CDK7 inhibition decreases STAT3chromatin binding and expression of highly transcribed target genes like MYC, PIM1, MCL1, CD30, IL2RA, CDC25A and IL4R.

One of CDKs is cycle dependent kinase 7 (CDK7) which phosphorylates the C-terminal domain (CTD) of RNA polymerase II (Pol I) at active gene promoters. The CDK-activating kinase (CAK), which is composed of CDK7, cyclin H, and MAT1, provides the T-loop phosphorylation necessary for the activation of CDKs 1, 2, 4, and 6, hence promoting cell cycle progression^15^. CDK7 inhibitors, including samuraciclib **(V)**^16^ and seliciclib **(VI)**^17^ (Fig. 1) are promising cancer treatments that disrupt transcription and the cell cycle to induce apoptosis in cancer cells. Remarkably, NIC has been shown to activate cleaved caspase-3 in breast cancer, causing Go/G1 cell cycle arrest, apoptosis, and tumor growth suppression^3^.

**Fig. 2:**
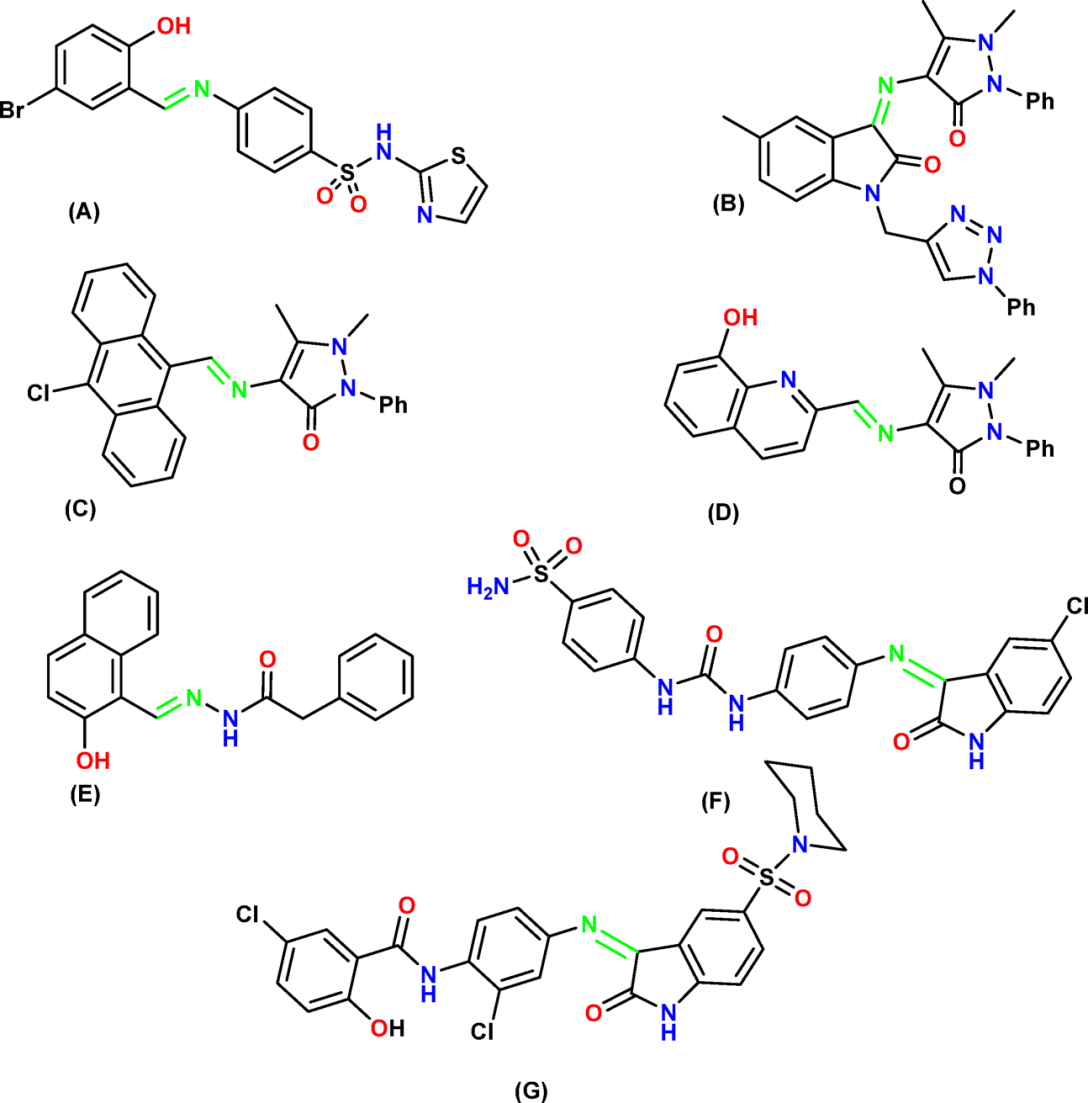
Schiff bases derivatives with anticancer properties.

In medicinal chemistry, Schiff bases and their derivatives are extremely adaptable substances with a wide range of biological activity. Antifungal, antibacterial, anticancer or antiproliferative are among these actions^18^. Schiff base **A** was shown to have an IC_50_ value of 16.0 µM against MCF-7 cells^19^ and, compound **B**, which is 2.1 times more potent than sunitinib, shows encouraging antiproliferative qualities against the MCF7 cancer cell line^20^. Another Schiff bases **C** and **D** showed IC_50_ values of 0.3 and 2.2 mM, respectively, on the same cell line after 48 h^21^. Compound **E** recently demonstrated strong cytotoxic impact, particularly on the human colorectal cancer cell line (HCT116, IC_50_ = 0.329 µg/ml) with a selectivity index (SI) of 15.93^22^. Furthermore, NIC-linked isatin Schiff base, **F** exhibits antiproliferative activity against HCT116, IC_50_: 20.05 ± 2.2 μm mediated by apoptosis^23^, while isatin-Schiff base **G** exhibits significant cytotoxicity against breast tumor cells, MDA-MB-231 and MCF-7 by blocking the MDA-MB-231 cell cycle at the G2/M phase and by potently inhibiting VEGFR-2 at nanomolar concentrations^24^ Based on the aforementioned findings and as a continuation of our previous efforts in the field of design and synthesis of new anticancer agents^25–29^, a new series of NIC-derived Schiff bases were designed using the primary pharmacophoric features of the JAK1 inhibitor, NIC, (Fig. 2).

### Rationale

The most popular term for the Schiff bases is the azomethine group, which is represented in organic chemistry as RHC = N–R1. Because the compound’s carbon and nitrogen atoms make it particularly reactive, the prevalence of lone pairs is explained by its sp2 hybridization which contribute to many Schiff base complexes. Due to their significant attributes, such as flexibility, simplicity, and utility, these compounds are of varying significance^30^. By combining niclosamide amine, **2** to multiple hydrophobic moieties (R) *via* CH = N linker, we were able to transform NIC with MLogP 2.44 into NIC-Schiff bases with higher MLogs, hence improving the pharmacokinetic and/or pharmacodynamic properties of NIC as an anticancer agent.

**Fig. 3:**
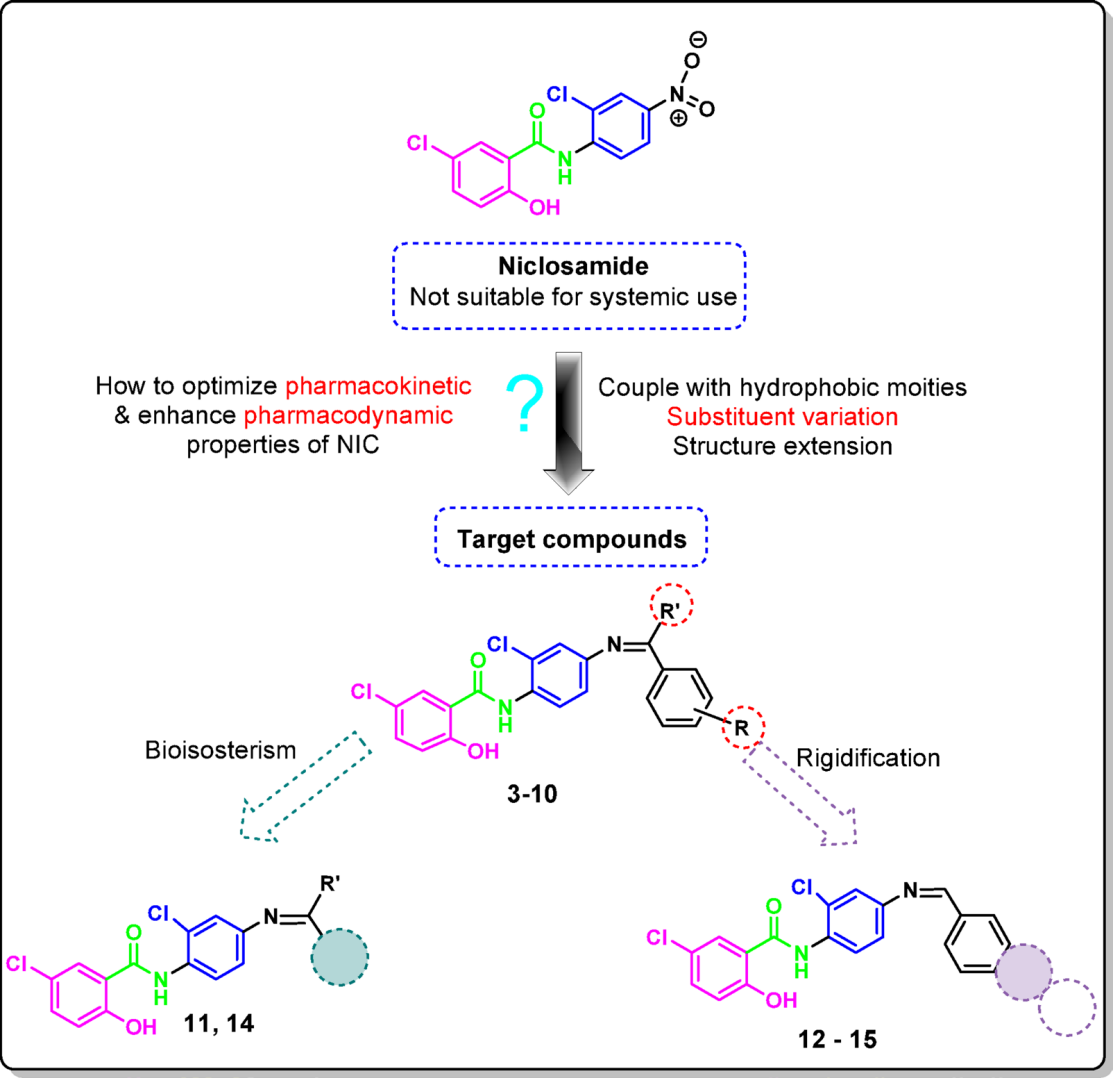
Rationale of the work to improve oral pharmacokinetic and pharmacodynamic properties.

All aspects considered, Schiff bases are a fascinating class of compounds with many properties and applications used in everything from fundamental chemistry to industry and medicine^31^. In order to produce NIC-Schiff’s bases, the nitro functionality is reduced to an aromatic amino group and condensed with different aldehydes or acetophenone to generate novel NIC derivatives. In our work plan, we used the following drug design techniques: rigidification, ring variation, structural extension, and substituent alteration (Fig. 3). The cytotoxicity and selectivity of the potential NIC derivatives will be will be assessed. We will mechanistically investigate the JAK1 and CDK7 inhibitory actions of those that shown selective cytotoxicity; we hope that these compounds will work as stand-alone anticancer agents, allowing us to prolong the drug’s shelf life. In order to confirm the promising hits’ potential for apoptosis and cell cycle arrest in comparison to NIC, the caspase 1, 3, and 9 triggering capability was assessed for each examined cell line. Finally, *ADME* and docking studies will be carried out in order to precisely describe the pharmacokinetic and/or pharmacodynamic quality of niclosamide derivatives as anticancer agents.

## Results and discussion

### Chemistry

Zinc dust is introduced as a suspension to a combination of NIC and acetic acid in ethanol using drops of HCl and stirring at room temperature, this is a gentle eco-friendly technique for reducing the aromatic nitro group of NIC **1** to NIC-amine **2** as reported with m.p. 195–197 °C.

**Fig. 4:**
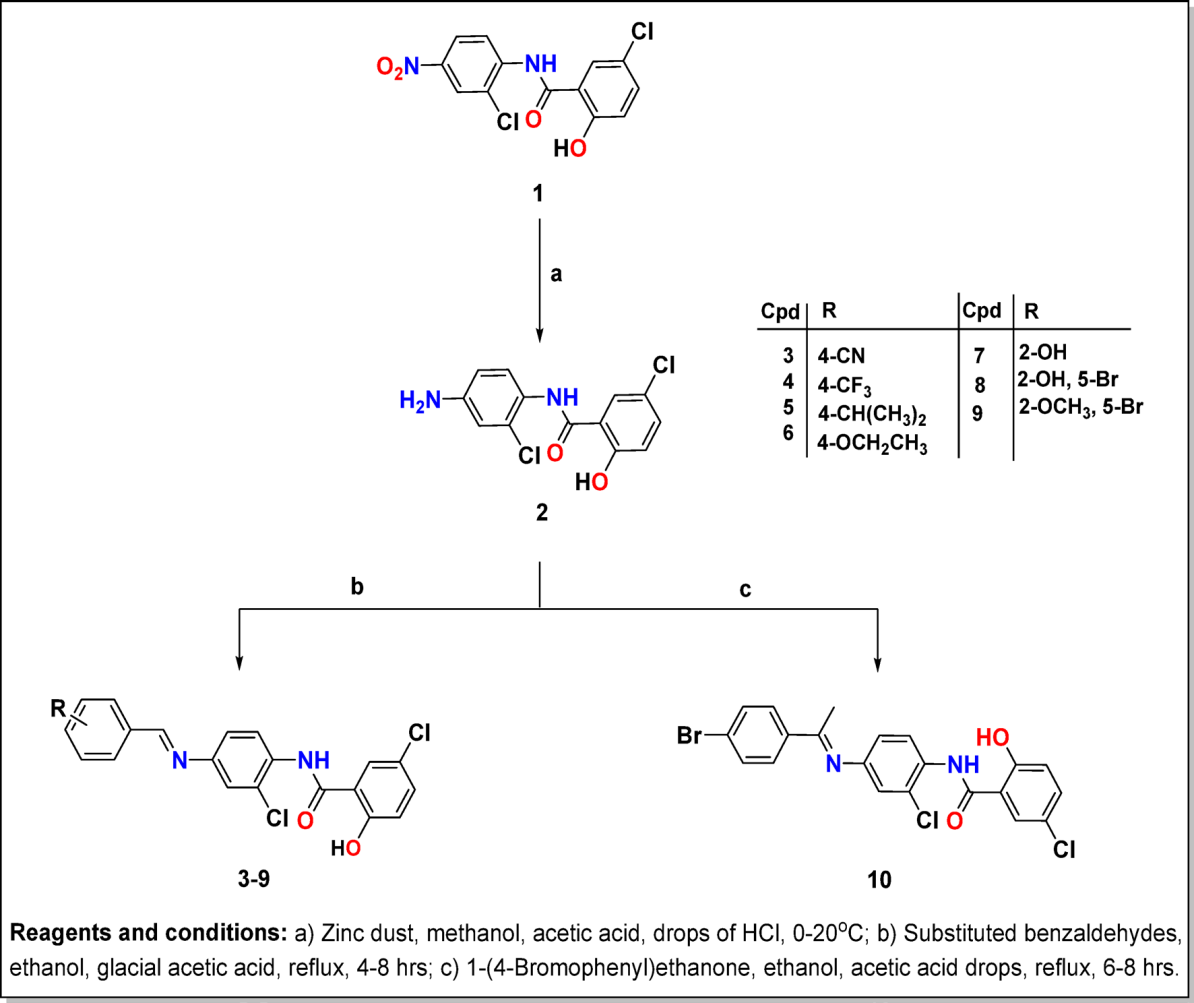
Synthesis of the target compounds **3–10**.

**Fig. 5:**
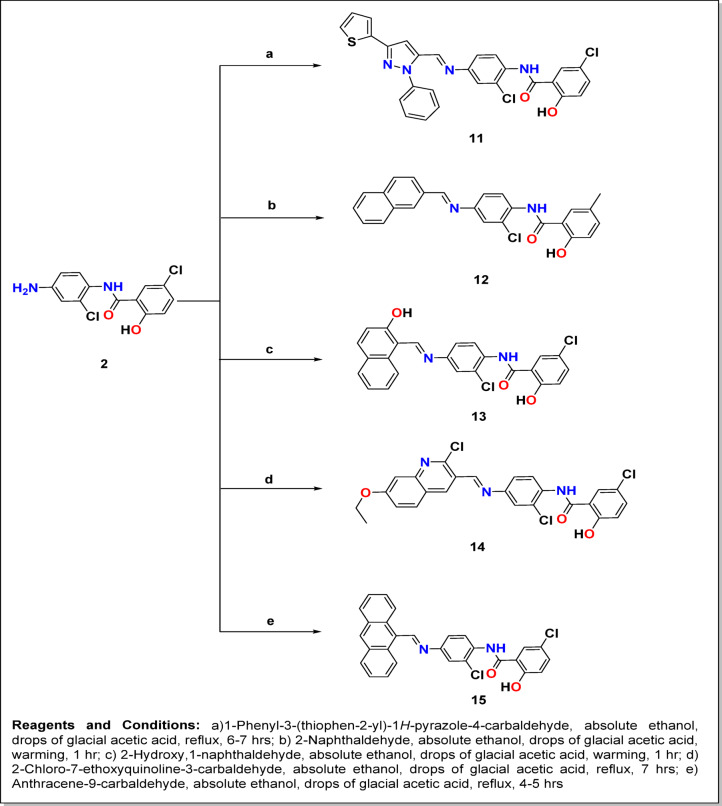
Synthesis of the target compounds **11–15**.

According to Figs. 4 and 5, novel Schiff’s bases^32–35^ of NIC, **3–15** were produced by the condensation reaction of NIC-amine **2** with various monocyclic/bicyclic/tricyclic aromatic aldehydes/acetophenone in absolute ethanol. During the reflux condition, a few drops of glacial acetic acid catalyzed this reaction for four to eight hours. It is noteworthy that Schiff bases **7** and **8** are formed on cold.

Using spectral data (IR, ^1^H NMR, ^13^C NMR, and MS), we were able to determine the structure of our target compounds **3–10**. The amino group absorption band disappeared along with the emergence of absorption bands at 1559–1623 cm^− 1^ that were ascribed to C = N in the IR. Additionally, a methine-H singlet signal at 8.56–8.81 ppm was shown by ^1^H NMR, confirming the synthesis of our compounds. The structure of our target compounds **3–15** was elucidated based on spectral data. Compound **8**’s IR spectrum, for instance, revealed the absence of the forked band for the NH_2_ group, which was already present in the NIC, together with a new absorption band at 1620 (C = N) cm^− 1^ that was ascribed to C = N. Furthermore, ^1^H NMR confirmed our compounds with a methine-H singlet signal at δ 8.95 ppm along with, a singlet for H6 at δ 7.94 ppm and two doublets representing H3 and H4 with *J* 8.6 Hz emerged at δ 6.92 and 7.52 ppm. Additionally, the hydroxy salicylaldehyde moiety’s OH proton emerged at δ 12.27 ppm and vanished upon deuteration. ^13^C NMR showed a distinctive signal at 152.42 for (C = N), (cf. Supplementary S1).

Compound **14** was chosen to serve as an illustration of Schiff-bases employing the rigidification technique. IR (KBr, υ/cm^− 1^) showed a novel band at 1539 that represented C = N lacking the NIC’s NH_2_ band; ^1^H NMR (500 MHz, in DMSO-d6) showed methine-H as a singlet at δ 9.01 and ester functionality as a triplet-quartet pattern at δ 1.38 and 4.20 ppm. Additionally, the quinoline moiety indicated an increase in aromatic protons (4Hs) as two singlets at δ7.96, 8.90, and two doublets at δ 8.11 and 7.04. Further, ^13^C NMR (125 MHz, in DMSO-d6) δ ppm revealed two peaks at 156.61 (methine C = N) and 150.46 ppm (quinoline C = N) along with ester singlets at 14.70 and 64.74, (cf. Supplementary S1).

### Biological evaluation

#### In vitro viability assay

Using the MTT assay, the thirteen test compounds (Table 1) were evaluated for their in vitro anti-tumor activity against two breast cancer cell lines (MCF-7 and MDA-MB-231) and prostate cancer cells (PC-3)^36^. Preliminary screening of the compounds was carried out *via* % viability at 50 µM to ensure that the synthesized compounds have cytotoxic activity against the selected cell lines (Table 1).

**Table 1:** In vitro viability of the test compounds against MCF-7, MDA-MB-231, and PC-3, cell lines.

Comp.	MCF-7	MDA-MB-231	PC3
NIC	20.0 ± 7.86	10.5 ± 1.51	7.70 ± 0.46
3	48.1 ± 26.68	84.4 ± 1.07	122.1 ± 15.93
4	23.4 ± 9.98	63.0 ± 0.43	37.5 ± 5.69
5	18.2 ± 6.22	81.0 ± 3.26	43.6 ± 1.55
6	75.2 ± 15.82	99.8 ± 2.38	101.0 ± 8.09
7	42.0 ± 0.50	37.7 ± 1.8	33.9 ± 00.8
8	14.7 ± 1.80	39.7 ± 3.2	8.2 ± 1.50
9	19.5 ± 1.56	25.5 ± 3.13	48.8 ± 28.98
10	16.6 ± 2.55	72.3 ± 1.14	66.1 ± 7.64
11	14.5 ± 0.50	32.6 ± 7.50	32.1 ± 5.40
12	40.1 ± 3.06	87.7 ± 5.36	59.0 ± 1.56
13	17.6 ± 5.20	77.5 ± 6.15	61.7 ± 2.97
14	11.3 ± 2.43	31.9 ± 7.68	24.7 ± 1.13
15	23.1 ± 10.81	16.1 ± 1.88	26.0 ± 1.73

##### SARs study

**Fig. 6:**
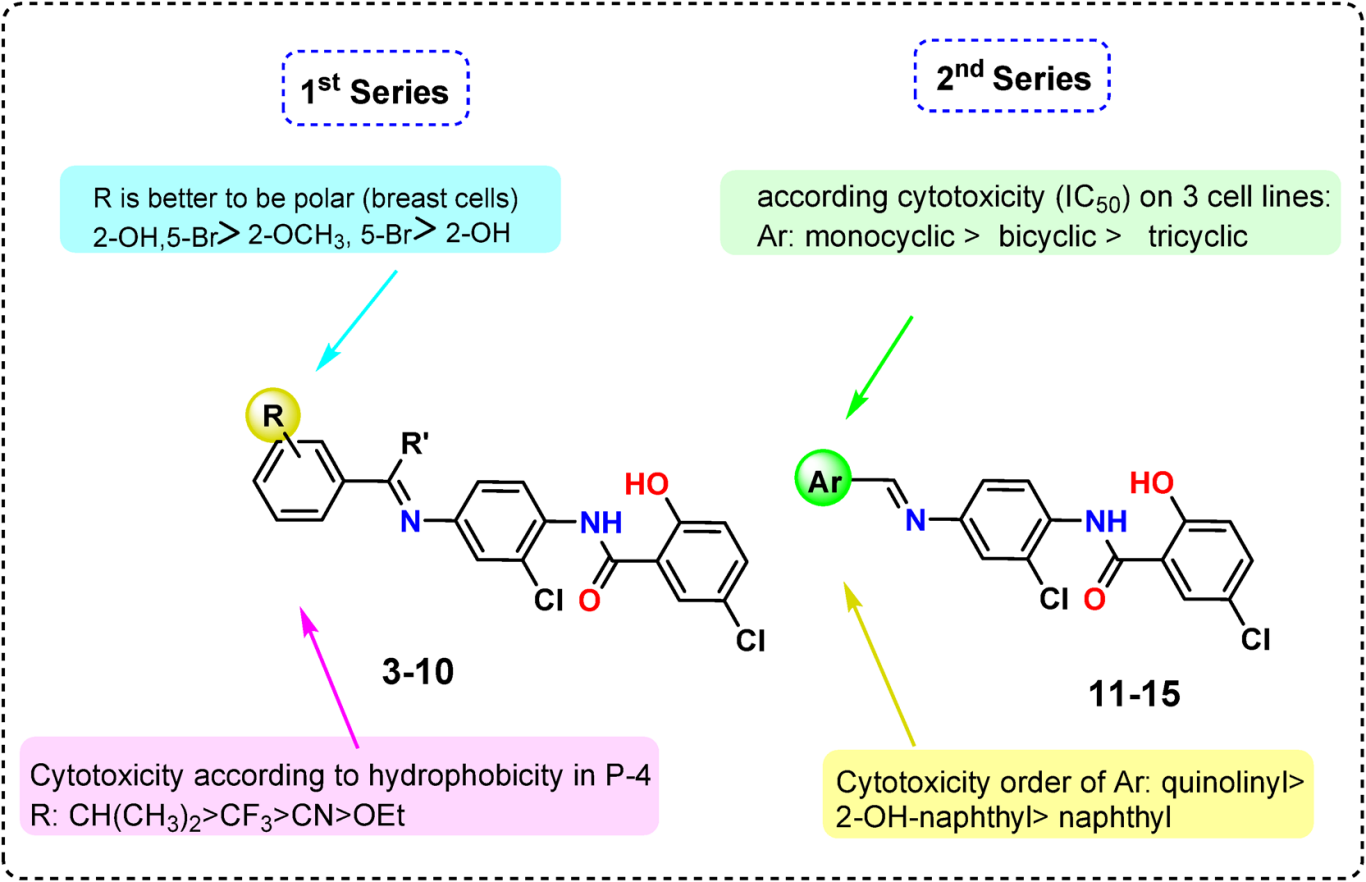
Main structure-activity relationships derived from this study.

The cytotoxicity order for Schiff bases **7–9** is **8 > 9 > 7** (see Table 1; Fig. 6). Compared to compound **9** with 2-OCH₃-5-Br, compound 8 with R: 2-OH-5-Br on the phenyl ring is somewhat more electron-rich and more activating. This is because the OH group has a stronger electron-donating resonance effect (+ R) than the OCH₃ group. The lack of the hydrophobic group (Br), which could contribute to anticancer activity, could be the cause of the analog’s lowest activity, **7** (R; 2-OH). The cytotoxicity order of Schiff bases **3–6** with the primary substituent at P-4 of phenyl is **5 > 4 > 3 > 6**, which corresponds to the hydrophobicity order of the 4-substituent where R: 4-CH (CH_3_)_2_ > CF_3_ > CN > OEt. Once more, Ar with a high electron density, such as in **14** (Ar: quinolinyl), was more cytotoxic than **13** (Ar: 2-OH-naphthyl), which is superior to **12** (Ar: naphthyl). Based on the three cell lines’ cytotoxicity results (IC_50_), monocyclic Schiff bases outperformed bicyclic ones, which are likewise more potent than tricyclic ones.

####  In vitro 5-dose (IC_50_) assay^37,38^

Using doxorubicin and NIC as reference drugs, five promising NIC derivatives **(7**,** 8**,** 11**,** 14**, and **15)** that met predetermined threshold inhibition criteria (one-dose assay) were selected for five-dose determination using the MTT assay on breast (MCF-7, MDA-MB-231), prostate (PC3), and normal (WI38) cell lines (Table 2). When NIC was condensed with monocyclic aldehyde, such salicylaldehyde, **7** and **8** were created. These compounds demonstrated strong safety margins against breast cancer (MCF-7, MDA-MB-231), with selectivity indices (SIs) ranging from 8.12 to 10.19. Their cytotoxicity against breast cancer was shown to be very strong to strong (MCF-7, IC_50_: 8.21–8.7, MDA-MB-231: 8.20–10.30 µM). They also showed moderate activity against the PC-3 (IC_50_: 28.77–29.70 µM).

The condensation of NIC with heterocyclic aldehyde, a derivative of pyrazole carboxaldehyde, produced Schiff’s base **11**, which had a notable single-digit IC_50_ on all cell lines under investigation (IC_50_ MCF-7: 2.85, MDA-MB-231: 4.61, PC-3: 7.69 µM) and SIs of 6.85, 4.23, and 2.54, respectively. In its effects against MCF-7 and PC-3 cells, target compound **11** unexpectedly demonstrated greater promise than the reference medication doxorubicin (IC_50_ MCF-7: 4.17, PC-3: 8.87µM). Additionally, **11** produced higher selectivity indices towards the studied cancer cell lines in comparison to doxorubicin (SI MCF-7: 1.61, MDA-MB-231: 2.11, PC-3: 0.76 µM). To look into other binding sites for the enzyme (JAK-1), Schiff’s bases of niclosamide were made utilizing a rigidification process using tricyclic and bicyclic aromatic aldehydes.

However, out of all the compounds that were studied, Schiff’s base **14**, which was created by condensation of NIC with bicyclic aromatic aldehydes exhibited the best selectivity indices towards the three screening cell lines (SI MCF-7: 11.13, MDA-MB-231: 10.16, and PC-3: 4.90). It also showed very excellent anticancer activity against breast cancer (IC_50_: MCF-7: 5.63, MDA-MB-231: 6.17µM) and considerable cytotoxicity for prostate cancer (IC_50_ PC-3: 12.79 µM).

Schiff’s base **15**, which was ultimately created *via* the reaction of NIC with tricyclic aromatic aldehyde, demonstrated a significant impact on breast cancer cell lines (IC_50_ MCF-7: 18.07, MDA-MB-231: 15.48 µM) and moderate activity against PC-3 (IC_50_: 32.62 µM). Thus, the anticancer order of their Schiff bases is bicyclic aldehyde > tricyclic aldehyde, (Table 2), (cf. Supplementary S2).

**Table 2:** IC_50_ values (µM^a^) of the test compounds against human normal and cancer cells in vitro.

Compd No	WI-38	MCF-7	SI	MDA-MB-231	SI	PC-3	SI
DOX	6.72 ± 0.5	4.17 ± 0.2	1.61	3.18 ± 0.1	2.11	8.87 ± 0.6	0.76
NIC	> 100	0.95 ± 2.3	> 105	2.87 ± 2.1	> 34.8	0.33 ± 2.9	> 303
7	83.68 ± 4.2	8.21 ± 0.5	10.19	10.30 ± 0.9	8.12	28.77 ± 1.8	2.91
8	76.48 ± 3.9	8.70 ± 1.5	8.79	8.20 ± 1.7	9.33	29.70 ± 2.3	2.58
11	19.52 ± 1.3	2.85 ± 0.1	6.85	4.61 ± 0.3	4.23	7.69 ± 5.3	2.54
14	62.69 ± 3.5	5.63 ± 0.2	11.13	6.17 ± 0.4	10.16	12.79 ± 0.9	4.90
15	27.15 ± 1.8	18.07 ± 1.4	1.5	15.48 ± 1.2	1.80	32.62 ± 2.0	0.83

^a^ IC_50_ values are the mean ± S.D. of three separate experiments. 1–10 (very strong). 11–20 (strong). 21–50 (moderate). 51–100 (weak) and above 100 (non-cytotoxic).

#### Evaluation of inhibitory activity against JAK-1^39^

Niclosamide demonstrated antiproliferative action that, functions as a novel JAK-1/STAT3 inhibitor. Enzymes called Janus kinases (JAKs) are part of signalling networks that influence immune cell and haematopoietic activities. Additionally, our new NIC prodrugs 8 and 11 were evaluated for their mode of action against the JAK-1 enzyme using the immunosorbent assay (ELISA) technique since they show promising cytotoxic effects on MCF-7, MDA-MB-231, and PC-3. Remarkably, findings showed that Schiff bases coupled to pyrazole (**11**, IC_50_: 0.017 ± 0.39) and bromosalycilaldehyde (**8**, IC_50_: 0.05 ± 0.29) have remarkable JAK-1 inhibitory activity that is higher than that of NIC, IC_50_: 0.22 ± 0.21 on the MCF-7 cell line. They demonstrated impressive JAK-1 inhibitory efficacy by running the same experiment on the MDA-MB-231 cell line: **8**, IC_50_: 0.03 ± 0.15, and **11**, IC_50_ 0.01 ± 0.11, surpassing NIC (IC_50_ 0.17 ± 0.05). Furthermore, compared to NIC (IC_50_: 0.20 ± 0.08), the PC-3 cell line data demonstrated stronger JAK1 inhibitory activity: **8**, IC_50_: 0.04 ± 0.12, and **11**, IC_50_ 0.10 ± 0.12. Notably, as compared to NIC, all of the target compounds had better inhibitory activity on all evaluated cell lines, (Table 3).

**Table 3:** In-vitro JAK-1 inhibition (IC_50_ µM).

Compound	MCF-7	MDA-MB-231	PC-3
NIC	0.22 ± 0.21	0.17 ± 0.05	0.20 ± 0.08
8	0.05 ± 0.29	0.03 ± 0.15	0.04 ± 0.12
11	0.017 ± 0.39	0.01 ± 0.11	0.10 ± 0.12

^a^ Data were expressed as Mean ± Standard Deviation (S.D.) of three independent experiments.

#### Evaluation of inhibitory activity against CDK7^40,41^

Cyclin-dependent kinase (CDK) 7 has a unique functional repertoire by virtue of its dual role in transcription and cell cycle progression. Importantly, it is now agreed that targeting transcription selectively limits the synthesis of mRNAs involved in tumor growth without causing outage of transcription of housekeeping genes. Thus, CDK7 has been considered as a viable therapeutic target in cancer. CDK7 inhibition induces cell cycle arrest, apoptotic cell death, and DNA damage through the STAT3-MCL1-CHK1 axis. Deletion of CDK7 inhibited PDAC cell proliferation, promoted apoptosis, and suppressed cell cycle progression in human and murine cell lines, suggesting that CDK-7 expression is required for chemoresistance^33^.

We attempted to further explore the effect of our hits on CDK7 expression on the three tested cancer cell lines. Our novel NIC-Schiff bases, **8**, and **11** were further tested for their action on apoptosis through CDK-7 enzyme inhibition using immunosorbent assay (ELISA) technique. Interestingly, in MCF-7 and PC3 cell lines, our hit **11** have amazing CDK-7 inhibitory activity, with % inhibition 80.7 ± 0.17 and 83.4 ± 0.09, which is significantly higher than NIC (MCF-7 64.3 ± 0.26 and PC3 66.6 ± 0.27) respectively. However, CDK-7 inhibitory action by Schiff base **8** was equivalent to that of NIC on MCF-7 and PC-3 cell lines, (Table 4).

**Table 4:** In-vitro CDK7 inhibition %.

Compound	MCF-7	MDA-MB-231	PC-3
	% inhibition	% inhibition	% inhibition
NIC	64.3 ± 0.26	71.6 ± 0.17	66.7 ± 0.27
8	64.5 ± 0.16	66.6 ± 0.91	66.7 ± 0.12
11	80.7 ± 0.17	68.1 ± 0.42	83.4 ± 0.09

Since compound **11** shown a dual inhibitory impact on JAK1 and CDK7 in the previous mechanistic investigation, further investigation was planned to ascertain whether it might cause cell cycle arrest, and trigger apoptosis *via* activating caspases 1, 3, and 9.

#### Effect on apoptosis

#####  Cell cycle analysis^24^

**Fig. 7:**
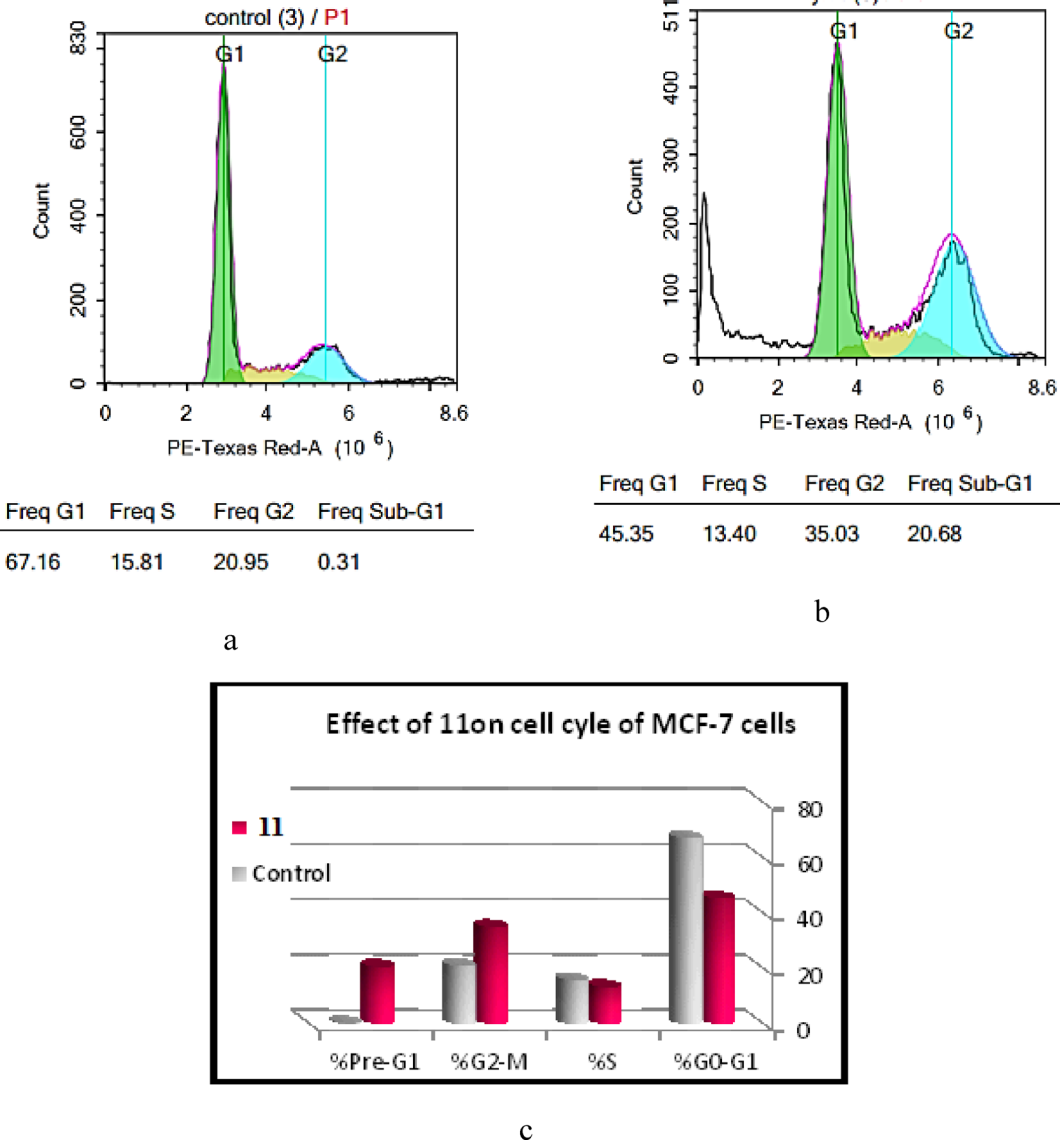
(**a**) Cell cycle analysis of MCF-7 cells in DMSO. (**b**) Cell cycle analysis of MCF-7 cells treated with **11**. (**c**) Effect of **11** and **control** on cell cycle of MCF-7 cells.

The target compound **11** produced cell cycle arrest at G2/M (35.03%) with overexpression of pre-G (20.68%) in contrast to the control (20.95%) and (0.31%), respectively. It dramatically raised the percentage of cells in the G2/M phase by 1.67 times and the proportion of cells in the Pre-G1 phase by around 66.71 times when compared to the control, according to cell cycle analysis results displayed in Figs. 7a–c. The presence of this sub-G1 peak is a strong indicator that the compound is activating an internal, programmed cell death pathway. Noticeably, this dramatic increase was coincided with a decline in the proportion of cells in the Go/G1 and S stages of the cell cycle in MCF-7 cells, (Table 5).

**Table 5:** Cell cycle arrest and apoptosis of MCF-7 cells by NIC analog, **11**.

Sample No.	%G0-G1	%S	%G2-M	%Pre-G1
11	45.35	13.40	35.03	20.68
Control	67.16	15.81	20.95	0.31

Data were presented as mean ± SD; *n* = 3.

##### Effect of 8 and 11 on active caspase-1,** 3**,** 9 levels**^42,43^

The family of cysteine proteases known as caspases is in charge of encouraging apoptosis, or cell death. To create the active pro-inflammatory cytokine interleukin 1β, the initiator caspase-1 cleaves latent prointerleukin 1β. It has been demonstrated that overexpression of caspase-1 causes apoptosis in both insect and mammalian cells^44^. Another initiator caspase called caspase-9 triggers downstream executioner caspases, such as caspase-3, which in turn starts a cascade that results in apoptosis. Caspase-3 is essential for triggering apoptosis in a number of human malignancies^45^.

**Fig. 8:**
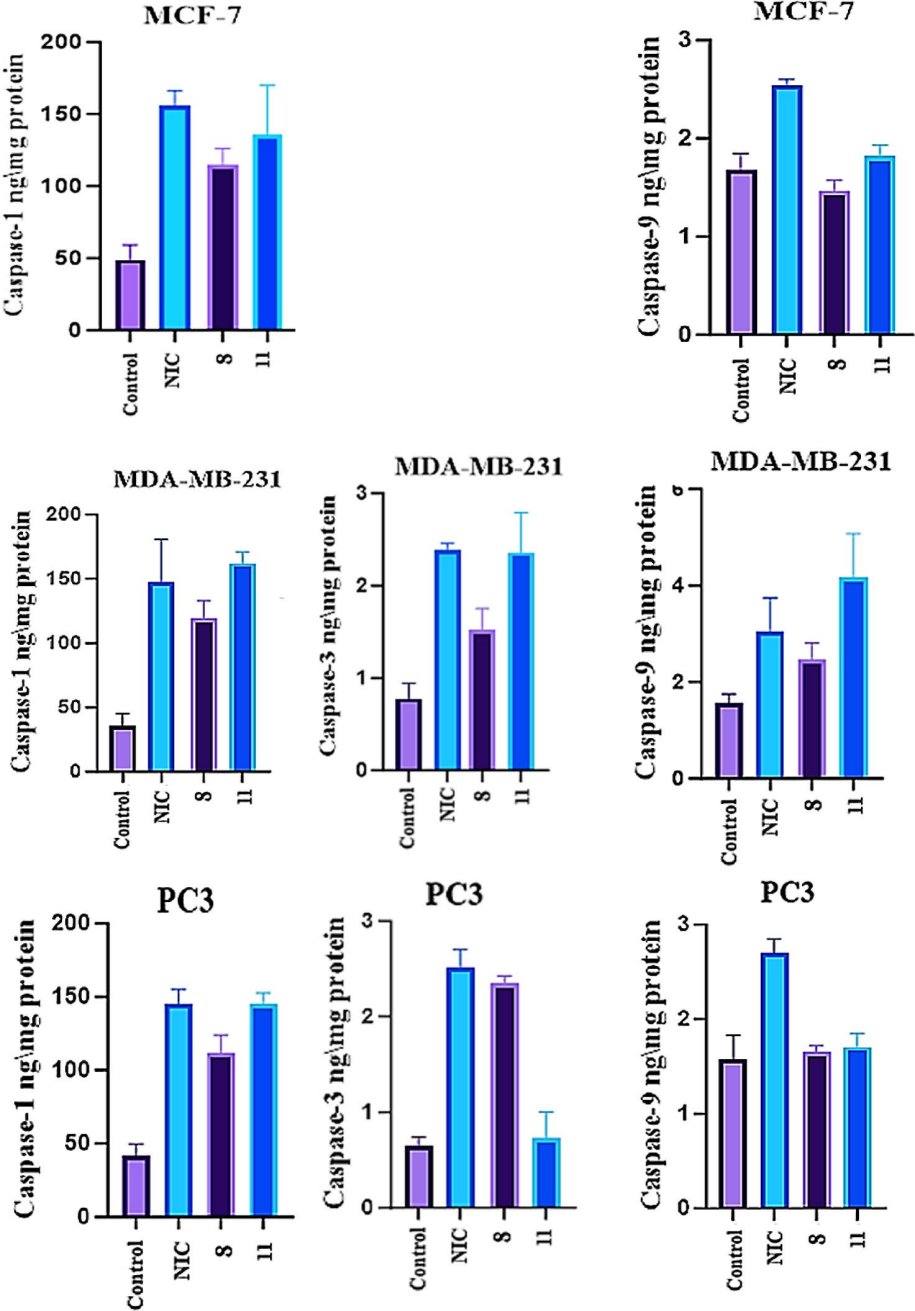
Caspases 1, 9 inducing action in MCF-7 and caspases 1, 3, 9 in MDA-MB-231 and PC-3 cell lines.

Since compound **8** was comparable to NIC and compound **11** showed more potent CDK7 inhibition according our results, they can induce apoptosis in cancer cells. The impact of the promising hits **8** and **11** on apoptosis markers, caspase-1, -3, and − 9, in MDA-MB-231, and PC-3 cell lines and caspase-1 and − 9 in MCF-7 cells was further assessed. For compounds **8** and **11**, our results showed that activation of caspases was lower than that of NIC in MCF-7 cells, (Table 6; Fig. 8).

**Table 6:** Relative fold change of test compounds on the level of caspases-1 and 9 at MCF-7 cells.

Sample No.	Caspase-1 (Pg./mL)	Fld.	Caspase − 9 (Pg./mL)	Fld.
8	114.8 ± 11.23	2.35	1.46 ± 0.11	0.87
11	135.8 ± 34.26	2.87	1.81 ± 0.12	1.08
NIC	155.4 ± 10.81	3.18	2.54 ± 0.06	1.51
Control	48.83 ± 10.35	1	1.68 ± 0.17	1

According to the data, **11** increased the caspase-1 level in MDA-MB-231 by over 4.60 times when compared to the control, whereas NIC only increased the level by 4.18 times. With regard to caspase-9, compound **11** again results in overexpression 2.69 times that of the control, while NIC influences 1.96 times the upregulation for its level. Additionally, compound **11** increases the level of caspase-3 by nearly 3.03 times in comparison to the control, which is similar to NIC’s 3.05 times, (Table 7; Fig. 8).

**Table 7:** Relative fold change of test compounds on caspases-1, 3 and 9 at MDA-MB-231 cell line.

Sample No.	Caspase-1 (Pg./mL)	Fld.	Caspase- 3 (Pg./mL)	Fld.	Caspase − 9 (Pg./mL)	Fld.
8	118.9 ± 14.29	3.38	1.52 ± 0.23	1.95	2.48 ± 0.34	1.59
11	161.8 ± 8.69	4.60	2.36 ± 0.42	3.03	4.14 ± 0.89	2.69
NIC	146.9 ± 33.65	4.18	2.38 ± 0.08	3.05	3.10 ± 0.68	1.96
Control	35.2 ± 10.0	1	0.78 ± 0.17	1	1.65 ± 0.20	1

Similar to NIC, the results for the PC-3 cell line indicated that **11** upregulated the caspase-1 level by 3.50 times as compared to the control. Additionally, compound **8** up regulates the caspase-3 level by nearly 3.70 times that of the control, which is an excellent outcome when compared to NIC, which raises the caspase-3 level by 3.90 times, (Table 8; Fig. 8).

**Table 8:** Relative fold change of test compounds on the level of caspases-1, 3, 9 at PC-3 cell line.

Sample No.	Caspase-1 (Pg./mL)	Fld.	Caspase- 3 (Pg./mL)	Fld.	Caspase − 9 (Pg./mL)	Fld.
8	111.9 ± 11.97	2.7	2.35 ± 0.75	3.7	1.65 ± 0.07	1.05
11	145.1 ± 7.72	3.5	0.73 ± 0.27	1.2	1.71 ± 0.14	1.08
NIC	145.2 ± 9.95	3.5	2.52 ± 0.18	3.9	2.70 ± 0.16	1.7
Control	41.5 ± 8.21	1	0.64 ± 0.10	1	1.57 ± 0.26	1

#####  Annexin V-FITC apoptosis assay^46^

**Fig. 9:**
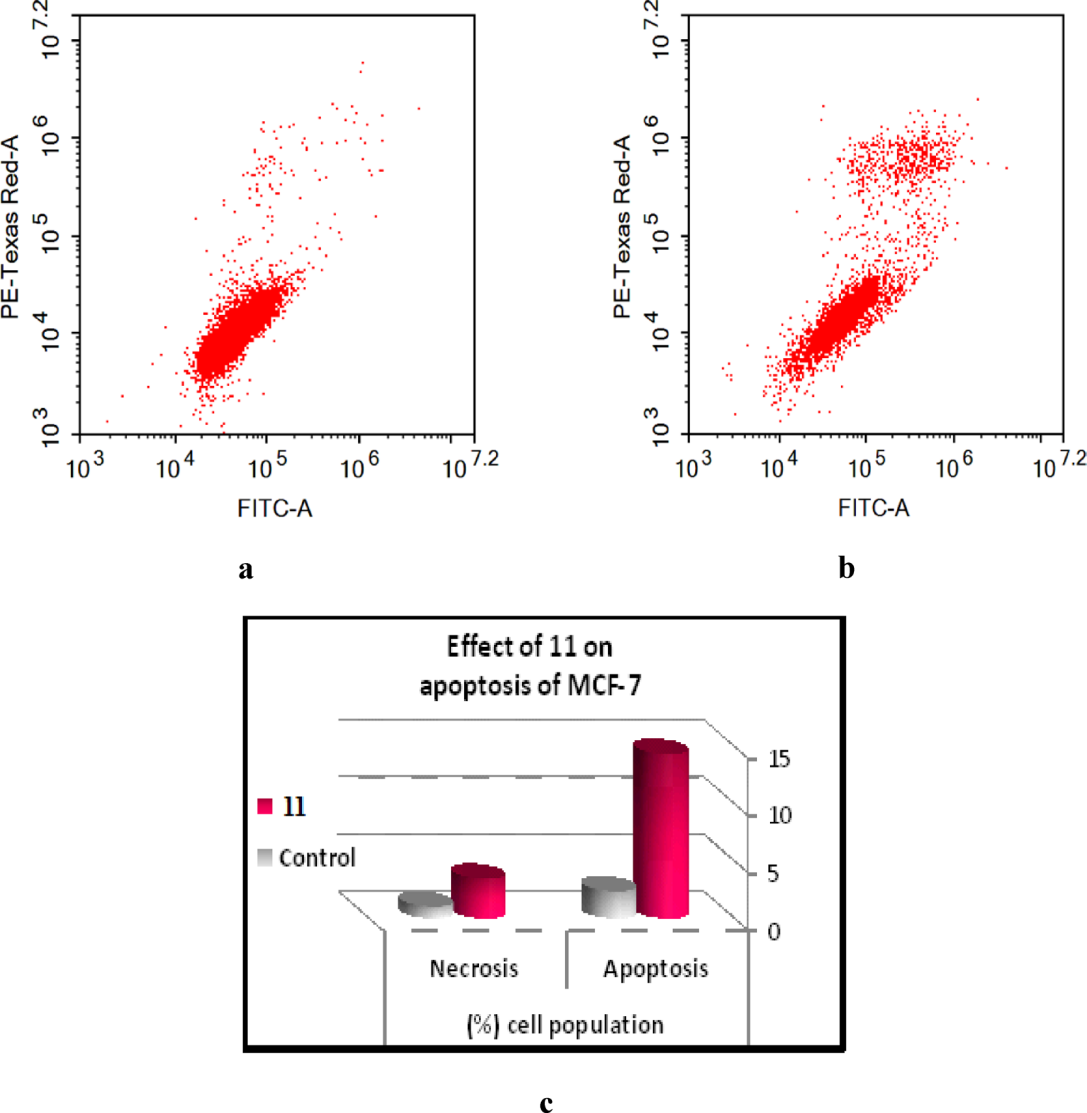
(**a**) Effect of control on apoptosis of MCF-7. (**b**) Effect of **11** on apoptosis of MCF-7, the quadrants in the cytograms represent the following: Necrotic cells (higher left quadrant of the cytogram); Late apoptotic cells (higher right quadrant of the cytogram); non-apoptotic and non-necrotic cells (living cells) (lower left quadrant of the cytogram); early apoptotic cells (lower right quadrant of the cytogram). (**c**) Effect of compound **11** on apoptosis of MCF-7 cells versus control.

Our results indicate that since compound **11** inhibits CDK-7, it can induce apoptosis in cancer cells. Annexin V binding detection by flow cytometer using propidium iodide (PI) double labeling was carried out in MCF-7 cells to confirm compound **11**’s ability to induce apoptosis and ascertain whether it did so by causing necrosis or apoptosis. The results showed that, in comparison to control untreated MCF-7 cells, **11** significantly increased necrotic cell death from 1.05% to 3.47% and apoptotic cell death from 1.34% to 10.92%. Target compound **11** had a substantial apoptotic effect, 8.15 times greater than that of the control trial, according to a comparative investigation on apoptosis. The two forms of cell death are supported by our results, mostly through the apoptotic pathway with a low necrosis ratio as inherent mechanisms underpinning compound **11**’s cytotoxicity, (Table 9; Fig. 9a–c).

**Table 9:** Effect (%) of compound **11** on apoptosis of MCF-7 cells.

Sample No.	Viable	Total	Early apoptosis	Late apoptosis	Necrosis
11	85.61	14.39	2.33	8.59	3.47
Control	97.61	2.39	0.56	0.78	1.05

#### *In silico* studies

##### *ADME* investigation

A computer analysis was used to evaluate the physicochemical and *ADME* properties of the generated compounds. Swiss*ADME*^47^ software was used to assess the compounds’ likelihood of being bioactive based on important features such as the Lipinski rule. Most of the test compounds’ physicochemical properties, as indicated in Table 10, are in line with the Lipinski parameters, which demonstrate zero violation^48,49^, (Table 11), indicating that these derivatives have promising drug-like properties. While compounds **11** and **15** both have molecular weights greater than 500, compound **14** showed a substantial degree of hydrophobicity with MLogP greater than 5. All compounds meet Veber’s criterion based on the topological polar surface area (TPSA)^50^ since their computed TPSA values are within the range required to pass through cell membranes. While compound No.**11** exhibited lesser absorption (71.83%) than NIC, all of the derivatives have higher absorption percentages (79.51–87.72%) when administered orally, according to the formula % ABS = 109 - (0.345 x TPSA), (Table 10). These systemically targeted compounds were guaranteed to have little or no CNS adverse effects because all did not pass the blood-brain barrier.

**Table 10:** Physicochemical and pharmacokinetic properties of new compounds and NIC.

Compd	HBD	HBA	M.Wt (gm\mol)	No. of rotatable bond	TPSA (Å²)	MlogP	BBB permeability	% ABS
NIC	2	4	327.12	4	95.15	2.36	No	76.17
3	2	4	410.25	5	85.48	3.52	No	79.51
4	2	6	453.24	6	61.69	4.73	No	87.72
5	2	3	427.22	6	61.69	4.84	No	87.72
6	2	4	429.30	7	70.92	3.80	No	84.53
7	3	4	401.24	5	81.92	3.37	No	80.74
8	3	4	480.14	5	81.92	3.96	No	80.74
9	2	4	494.17	6	70.92	4.17	No	84.53
10	2	3	478.17	5	61.69	5.00	No	87.72
11	2	4	533.43	7	107.7	4.34	No	71.83
12	2	3	435.30	5	61.69	4.87	No	87.72
13	3	4	451.30	5	81.92	4.31	No	80.74
14	2	3	485.36	5	61.69	5.49	No	87.72
15	2	5	514.79	7	83.81	3.77	No	80.09

Furthermore, the medicinal chemistry properties and drug-likeness of the test compounds were predicted (Table 11). It is essential to check for any PAINS alerts regarding the newly produced derivatives. A PAIN conducted by SwissADME revealed no alarms for any of the hits. The SwissADME Synthetic Accessibility (SA) Score is based primarily on the assumption that the frequency of molecular fragments in “really” attainable molecules correlates with the ease of synthesis; SA scores of all the analogues were found to be between 2.70 and 3.72, indicating that they can be easily synthesized on a large scale. Bioavailability, considered the most significant component affecting absorption, measures the amount of medication present in the plasma. It’s noteworthy that, with the exception of compound **11** (0.17), all the new compounds had bioavailability ratings (0.55) comparable to NIC. Additionally, all NIC-Schiff bases do not violate the Veber rule (Table 11). Overall, our hits were found to have acceptable medicinal chemistry criteria and drug-likeness values, potentially making them drug-like opportunities.

**Table 11:** Medicinal chemistry characters and drug likeness of new compounds and NIC.

Compound	S.A.	PAINS alert	Lipiniski’s violation	Veber violation	Bioavailability score
Niclosamide	2.01	0	0	Yes	0.55
3	2.71	0	0	Yes	0.55
4	2.73	0	0	Yes	0.55
5	2.84	0	0	Yes	0.55
6	2.82	0	0	Yes	0.55
7	2.70	0	0	Yes	0.55
8	2.82	0	0	Yes	0.55
9	2.92	0	0	Yes	0.55
10	2.72	0	0	Yes	0.55
11	3.72	0	1	Yes	0.17
12	2.79	0	0	Yes	0.55
13	2.92	0	0	Yes	0.55
14	3.19	0	1	Yes	0.55
15	3.01	0	1	Yes	0.55

##### Molecular docking

*Docking in JAK1*

From the online Protein Data Bank (PDB) database (www.pdb.org) the model of ATP-binding site of JAK (PDB ID: 4E4N, resolution: 1.85 Å)^51^ was downloaded and prepared for flexible molecular docking by Molecular Graphics Laboratory (MGL) Tools utilities. The preparation of this receptor involved removal of the surplus copies of the enzyme chains, non-bonded inhibitors, addition of polar hydrogens and merging of non-polar ones. Default Gasteiger charges were assigned to all atoms, water molecules had to be removed from the system. The following xyz coordinates of the grid box centers were applied. The grid box dimension of targeted proteins was determined using Auto-Dock (MGL-tools). The grid box was exported in text format. In the meanwhile, the target enzyme was exported in PDQT format. The active site grid box speciation was: Spacing (0.375), N.pts. (X; 59, Y; 40; Z; 40), and Center (X; 11.767, Y; 0.276; Z; -2.0). The docking was performed Auto-Dock Vina (MGL-tools) script. The results of docking were exported as Comma-separated files (CSV). Finally, the output of Auto-Dock Vina was then visualized using Discovery studio Biovia. Initially, we carried out docking for the co-crystallized ligand, followed by docking for the synthesized compounds.

**Fig. 10:**
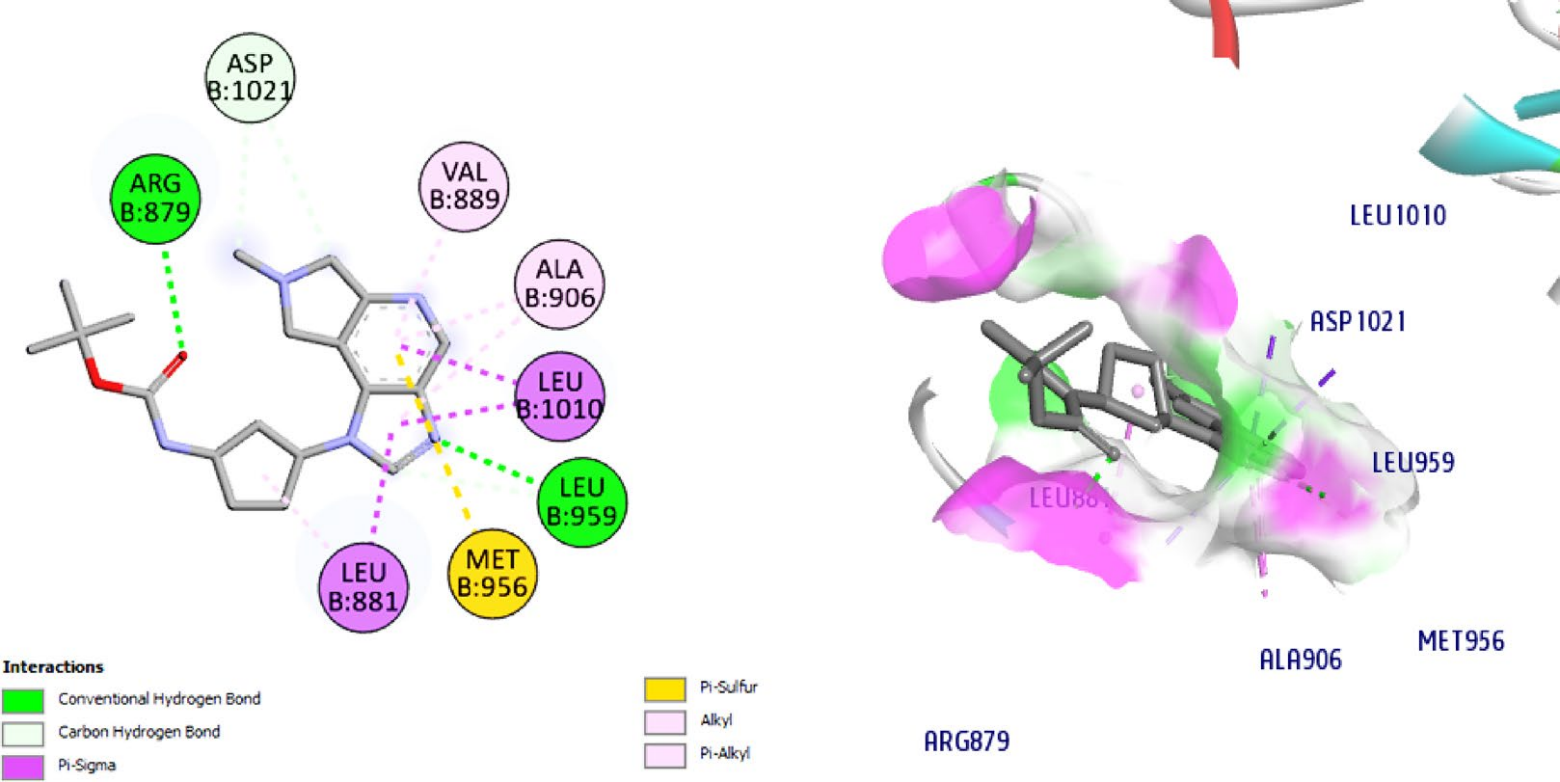
2D & 3D interactions of **ligand** with 4E4N.

**Fig. 11:**
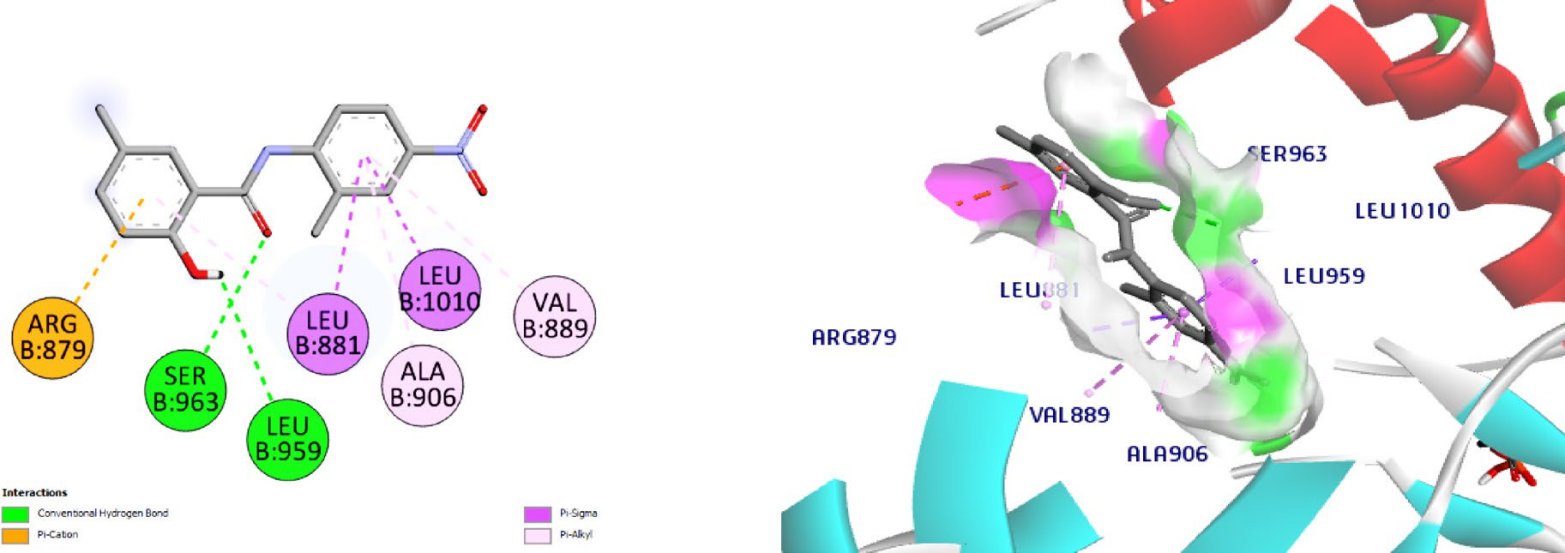
2D & 3D interactions of **NIC** with 4E4N.

On the centroid of the co-crystallized ligand, JAK1’s Leu881, Glu883, Val889, Ala906, Met956, Glu957, Phe958, Leu959, Gly962, Ser963, Glu966, Arg1007, Asn1008, Leu1010, Gly1020, and Asp1021 were produced, encircling the residues of the ATPbinding site. The affinity of the co-crystallized **ligand** for the JAK1 active site is -8.6 kcal / mol. The amino acids Leu959 (2.3 Å) was connected to the ligand through one H bond. The amino acid, Asp1021 is connected to the ligand through both H bond and C-H bond, (Fig. 10). The docking of **NIC** suggesting a lower affinity (-7.4) than the ligand; it displayed a 2 H bonds with Ser963 (2.6 Å) and the hinge region amino acid, Leu959 (2.7 Å), (Fig. 11).

**Fig. 12:**
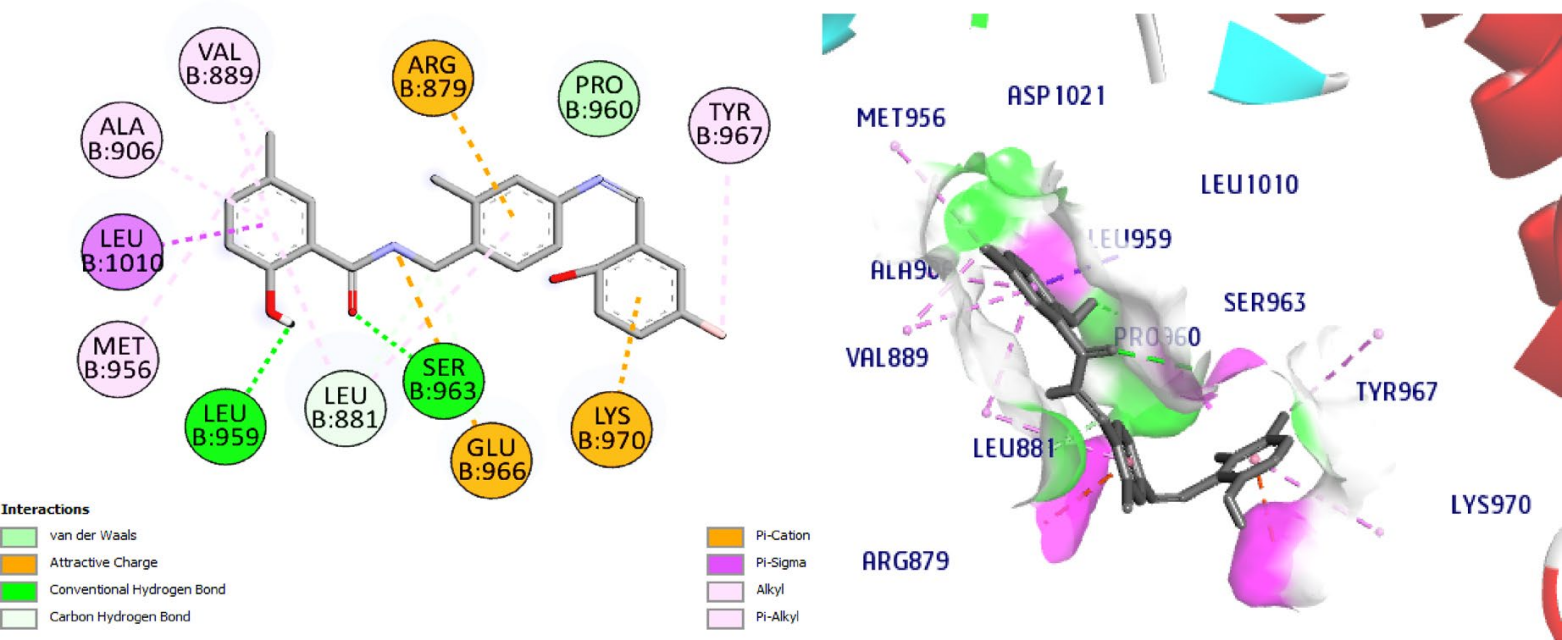
2D & 3D interactions of **8** with 4E4N.

**Fig. 13:**
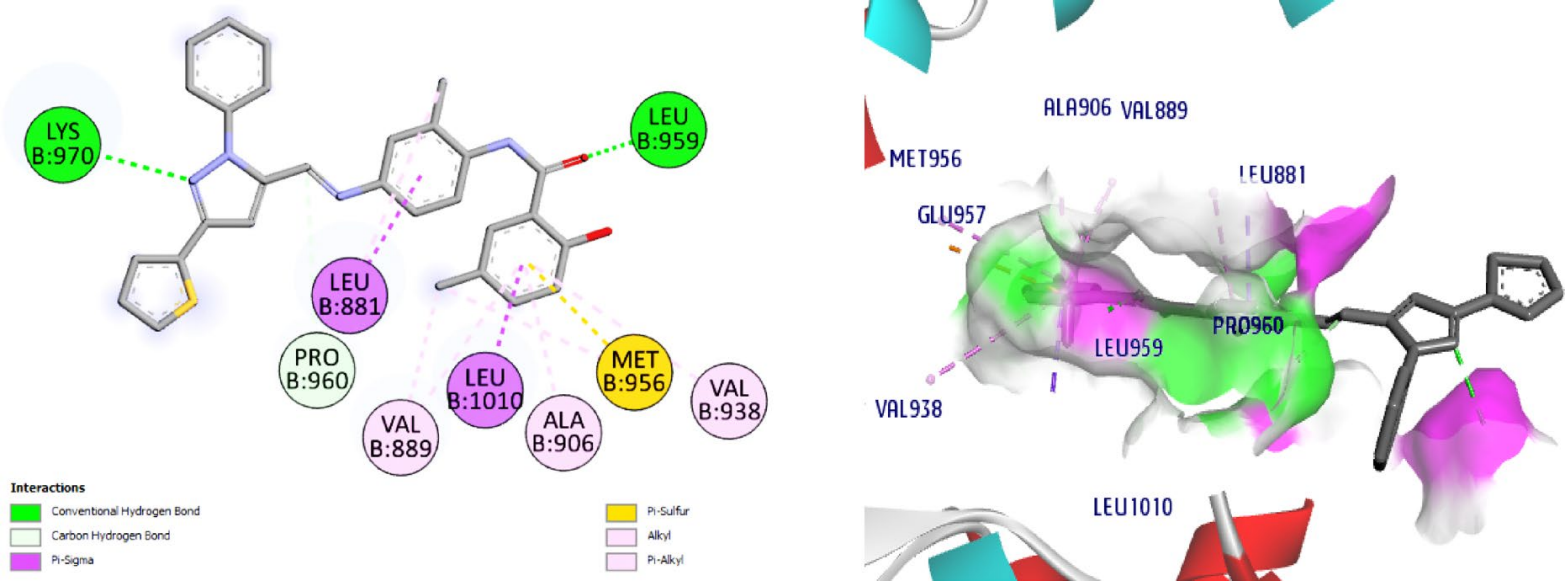
2D & 3D interactions of **11** with 4E4N.

Interestingly, our hits **8** and **11** displayed affinities for the JAK1 active site that were higher than **NIC** (-7.4), -8.9 and − 10.3, respectively. In reference to Schiff base **8**, it established two C-H bonds with Glu966 and Leu881 and two H bonds with Ser963 (2.9 Å) and the hinge region amino acid, Leu959 (2.1 Å) are seen in Fig. 12. Concerning compound **11**, it exhibited two conventional H bonds with Lys970 (2.7 Å) and the crucial amino acid residue, Leu959 (2.5 Å). Docking results also revealed one C-H bond with Pro960, (Fig. 13). Collectively, the ligand, NIC and our hits showed more hydrophobic connections to the following amino acid residues: Arg879, Leu881, Val889, Ala906, Met956 and Leu1010. Nevertheless, our hits **8** and **11** showed additional hydrophobic interactions with Val938, Glu966, Tyr967, and Lys970, indicating that our hits had higher binding affinities than NIC and can bind to the ATP binding pocket, a crucial part of JAK1 inhibition.

*Docking in CDK7*

**Fig. 14:**
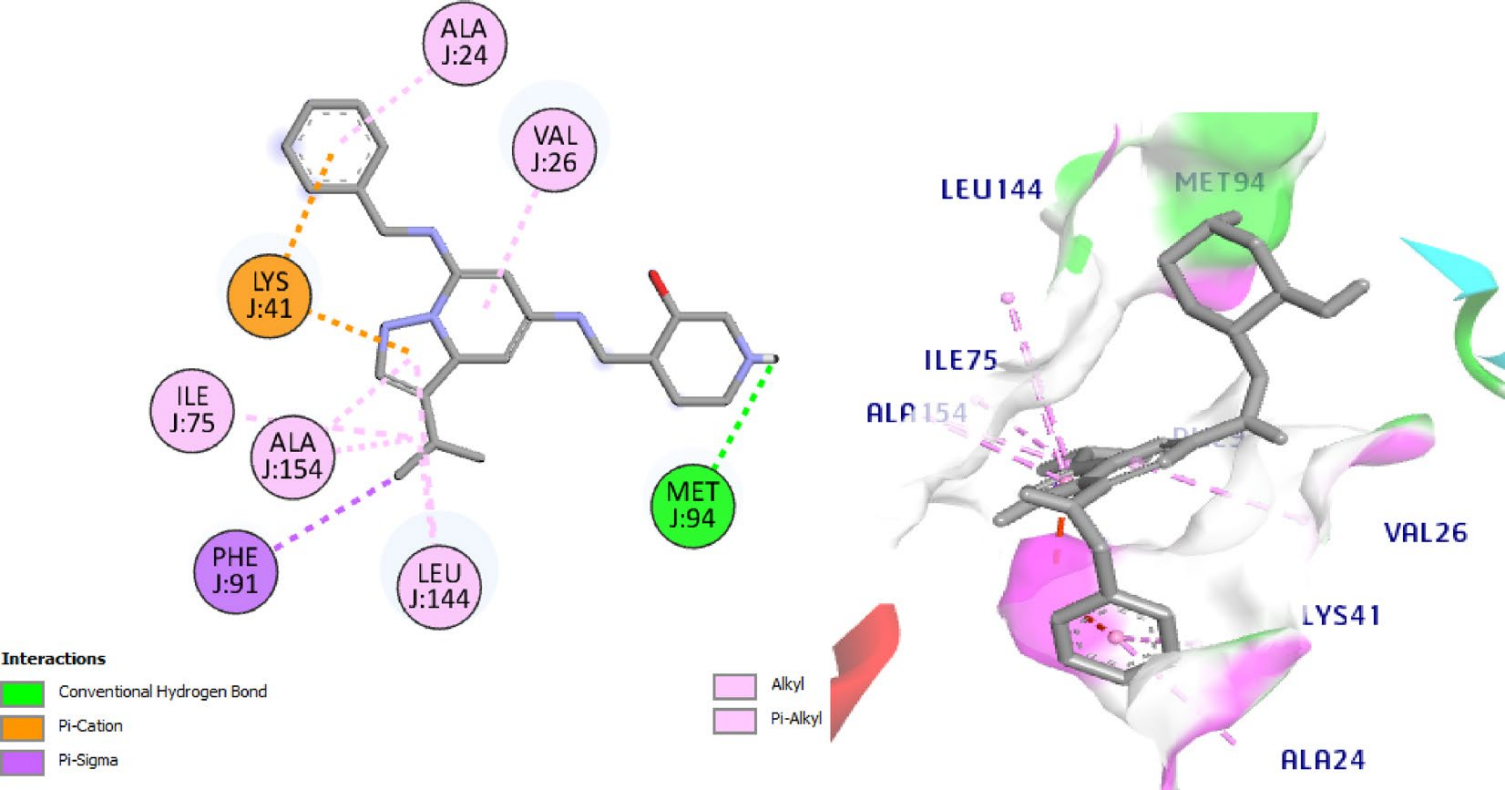
2D and 3D interactions of Co-crystalized ligand with 7B5O.

**Fig. 15:**
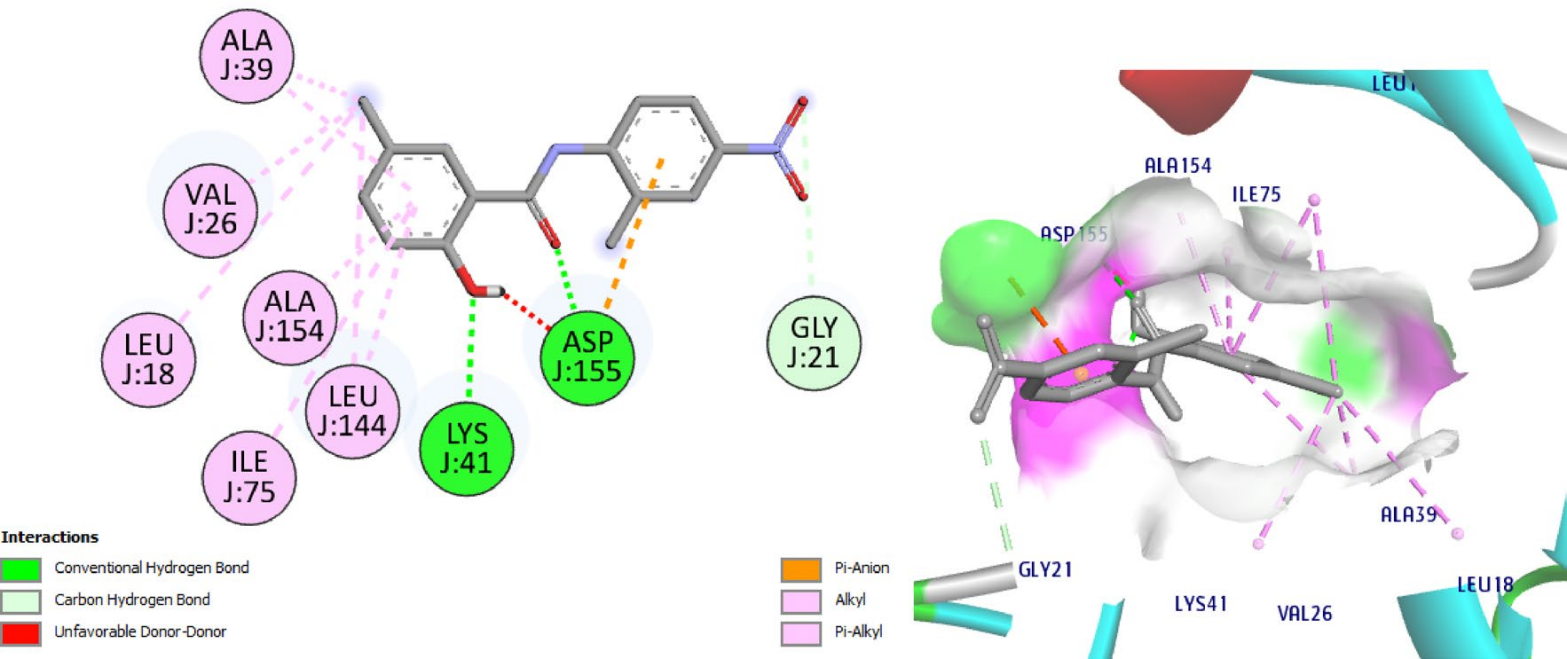
2D & 3D interactions of **NIC** with 7B5O.

The most active compounds **8** and **11** from the screening and NIC were docked with CDK7 to understand its interaction pattern at the ATPbinding site. Samuraciclib^52,53^ is an orally available, selective inhibitor of CDK7 (cyclin-dependent kinase 7) with potential anticancer activity is used as ligand where the 7B5O structure bound with samuraciclib was chosen for our study. Samuraciclib occupies the ATP-binding site of CDK7 (dock score = -8.7 kcal/mol) and shows a vital H-bonding with the hinge region residue, Met94 (2.1 Å), *via* piperidine ring NH functionality and hydrophobic interaction with the key amino acid Lys41. Samuraciclib displays other hydrophobic interactions with Ala24, Val26, Ile75, Phe91, Leu144, Ala154 residues, (Fig. 14). The docking of **NIC** indicated that it perfectly occupies the ATP-binding site of CDK7 with a dock score of -7.7 kcal /mol. The phenol- OH forms HBA with the hinge-pocket residue, Lys41 (2.3 Å) and carbonyl-O form another H bond with Asp155 (2.9 Å) and hydrophobic interaction with Leu18 and Gly21, Val26, Ala39, Ile75, Leu144, (Fig. 15).

**Fig. 16:**
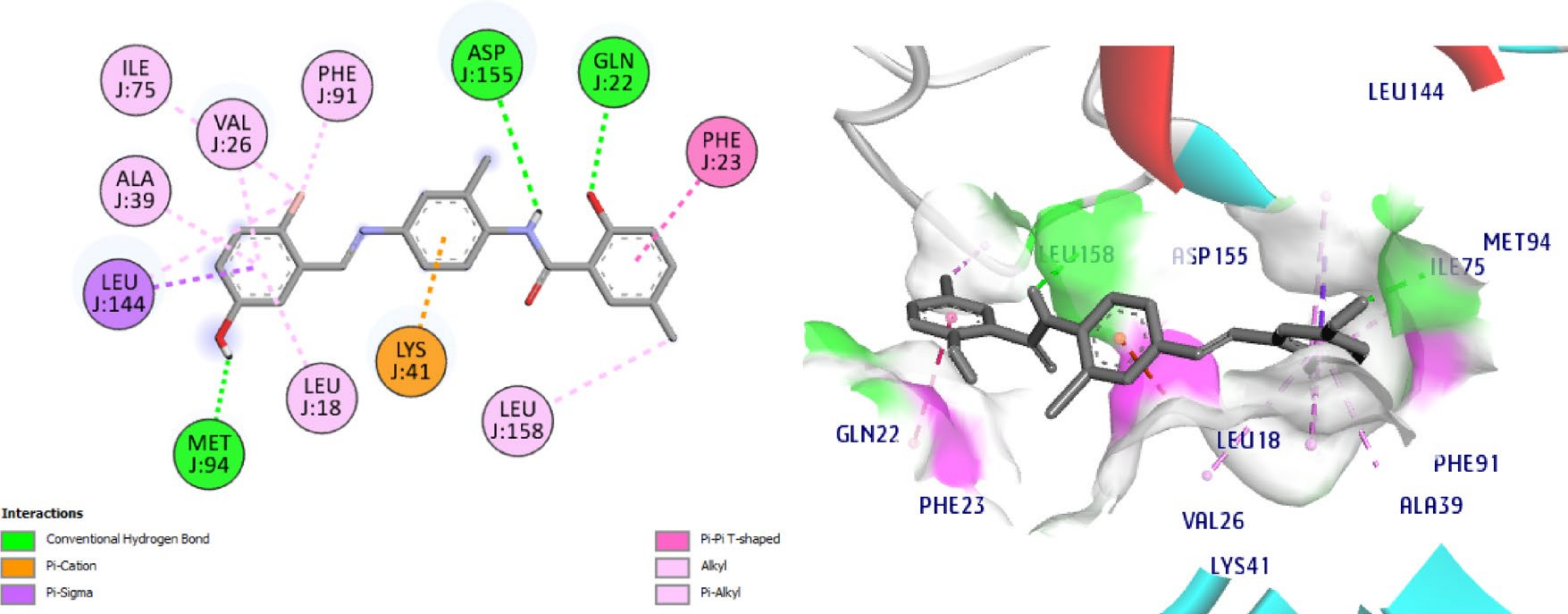
2D & 3D interactions of compound **8** with 7B5O.

Interestingly, docking of the target compounds, **8** and **11** revealed their dock scores of -8.9 and − 9.9 kcal/mol respectively which are positively impacted on the affinity of our new hits to the active site of CDK7 enzyme. Most of the essential H-bonding interactions of samuraciclib were reproduced in NIC-Schiff base **8**, which could be the reason why it exhibits significant CDK7 inhibitory activity. Its docking shows three H bonds with Met94, Asp155 and Gln22. It worth to mention that, the new aryl moiety of the NIC-Schiff base, **8** makes H bond between Ph-OH with the hinge region residue Met94, in addition to many hydrophobic connections with Leu18, Val26, Ala39, Ile75, Phe91 Leu144 amino acid residues. Also, it forms 2 H bonds connecting the original structure of NIC to Asp155 and Leu18 amino acids in addition to Pi-Cation interaction with the fourth important amino acid Lys41, (Fig. 16).

**Fig. 17:**
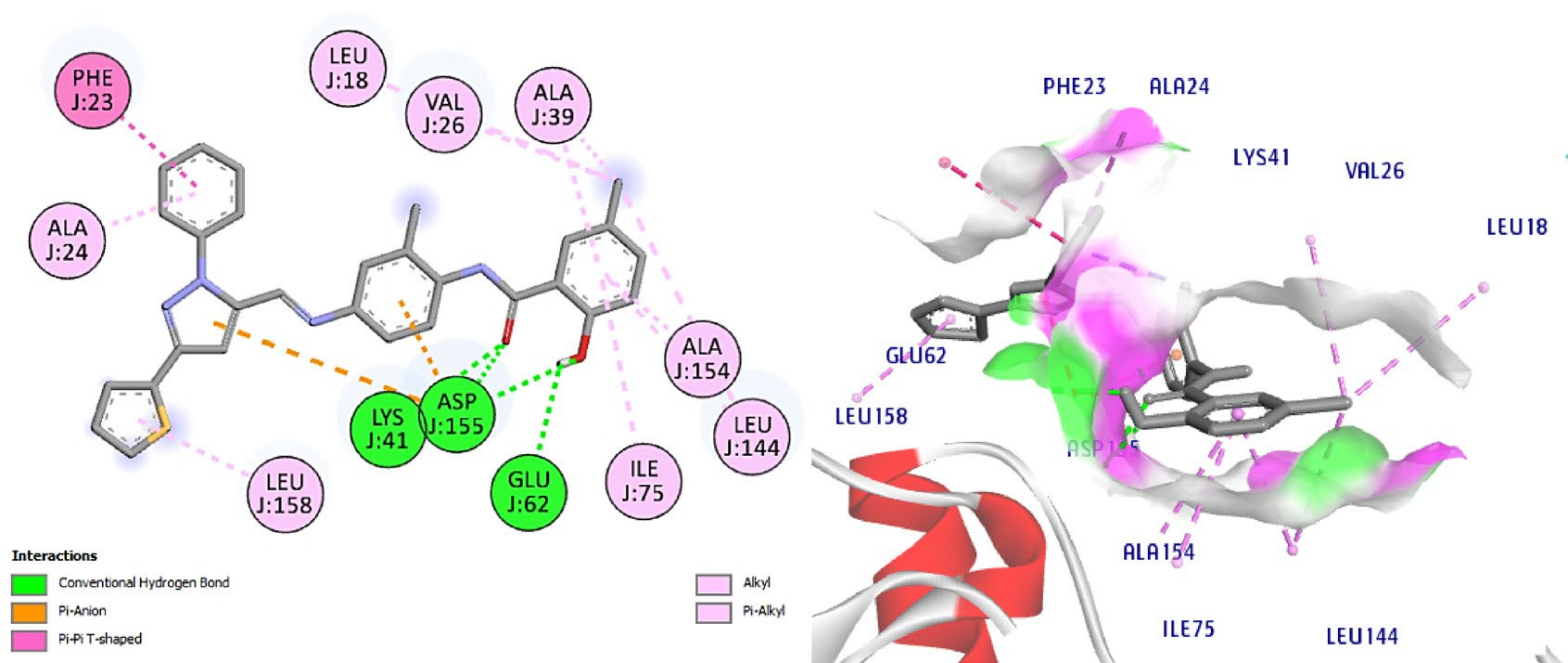
2D & 3D interactions of compound **11** with 7B5O.

Regarding the docking results of compound **11**, it shows four conventional H bonds with Lys41 (2.0 Å), Asp155 (2.8 Å) and Glu62 (2.5 Å). Here the new aryl moiety impacted more affinity *via* hydrophobic connections with the amino acids Phe23, Ala24 and Leu158, (Fig. 17). Our NIC-Schiff bases **8** and **11** also have various hydrophobic interactions with the following amino acids; Phe23, Ala24, Val26, Ala39, Ile75, Leu144, Leu158.

**Fig. 18:**
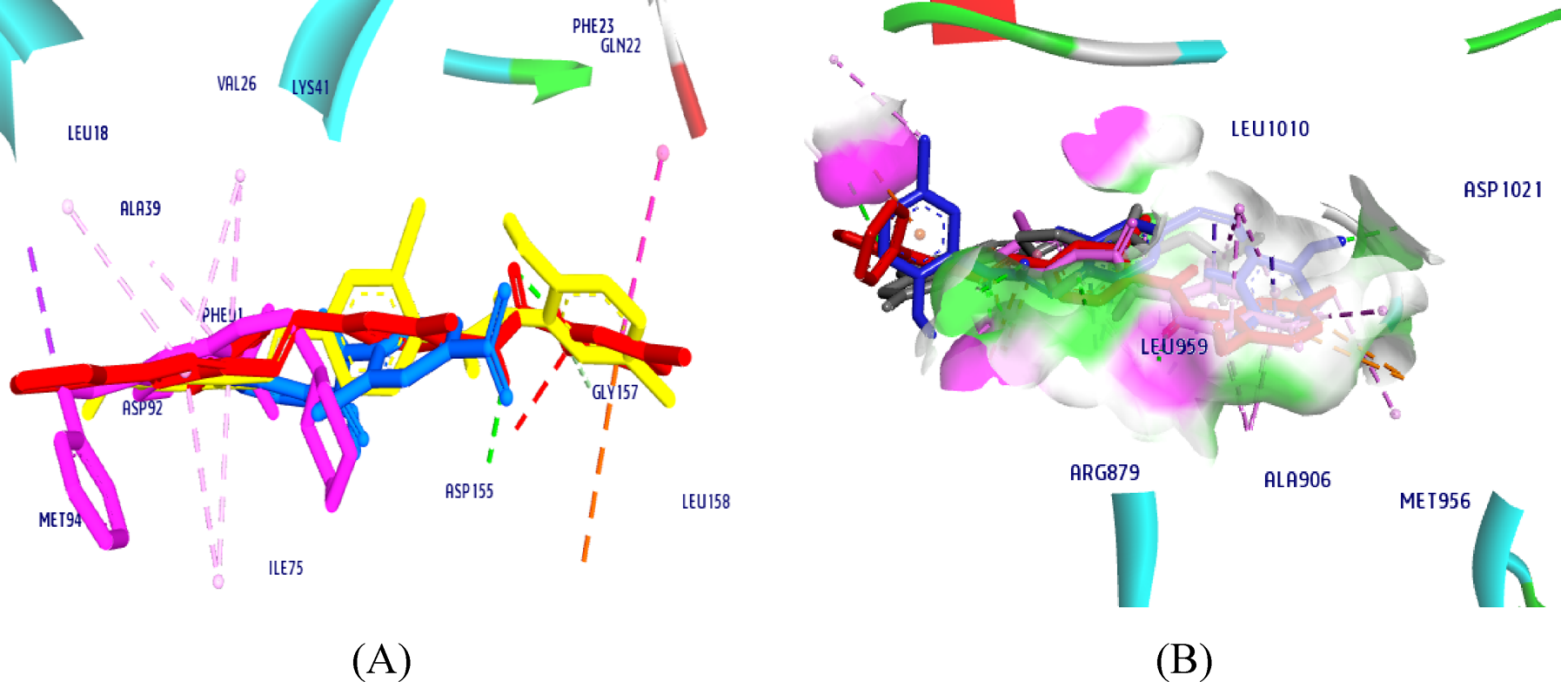
Superimposition of **NIC**, **8** and **11** with **ligand** in the active site of (**A**) CDK7 and (**B**) JAK.

Molecular docking studies can show the dual inhibitory effect of our target compounds **8** and **11** by proving the higher molecule’s binding affinity to the active sites of both JAK1 and CDK7 enzymes. Binding energies and interaction visualization are typically used to assess this binding. Overall, we discovered a dual inhibitory mechanism of CDK7-JAK1 by superimposing our candidates **8** and **11** with NIC and their ligands, (Fig. 18).

*Molecular dynamic simulation*

**Fig. 19:**
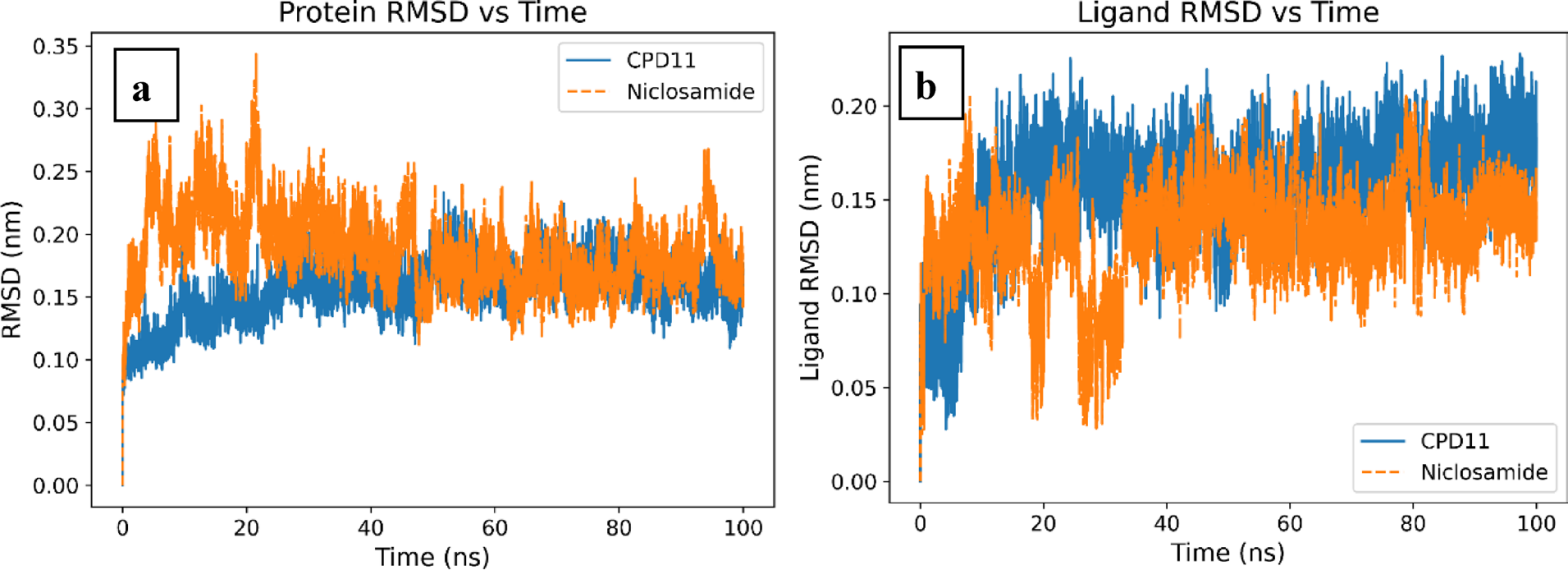
(**a**) Molecular dynamic simulation RMSD of protein, (**b**) RMSD variation between 2 ligands.

The best-scoring complex with of JAK1, compound 11 and NIC were was subjected to molecular dynamics simulations using the Groningen Machine for Chemical Simulations (GROMACS, version 2025.2)^54^. Protein topologies were generated with the CHARMM36 force field (July 2022, LJ-PME release)^55^, and ligand parameters were obtained using the CHARMM General Force Field (CGenFF)^56^. To validate the stability and reliability of the docking predictions, molecular dynamics simulations were performed over 100 nanoseconds, providing a dynamic view of protein-ligand behavior under physiological conditions. The RMSD analysis of the protein backbone demonstrated that both JAK1-compound **11** and JAK1-NIC complexes achieved stable conformations, with RMSD values stabilizing after an initial equilibration period. Importantly, the compound **11** complex exhibited slightly lower RMSD fluctuations compared to NIC, confirming that the predicted binding pose remains stable throughout the simulation and that compound **11** induces less structural perturbation in the protein. This stability is crucial because it indicates that the binding mode observed in docking is maintained over time and is not merely an artifact of the static docking calculation, (Fig. 19a). The ligand RMSD analysis further reinforced these findings by showing that compound **11** maintained a more consistent position within the binding pocket compared to NIC. The reduced positional variability of compound **11** suggests that its diverse interaction network effectively anchors the molecule, preventing significant drift or reorientation. In contrast, the slightly higher RMSD values observed for NIC indicate greater conformational flexibility or repositioning within the active site, which correlates with its lower binding affinity and fewer stabilizing interactions. This dynamic behavior provides molecular-level evidence that compound **11** superior docking score translates into enhanced binding stability under simulated physiological conditions, (Fig. 19b).

**Fig. 20:**
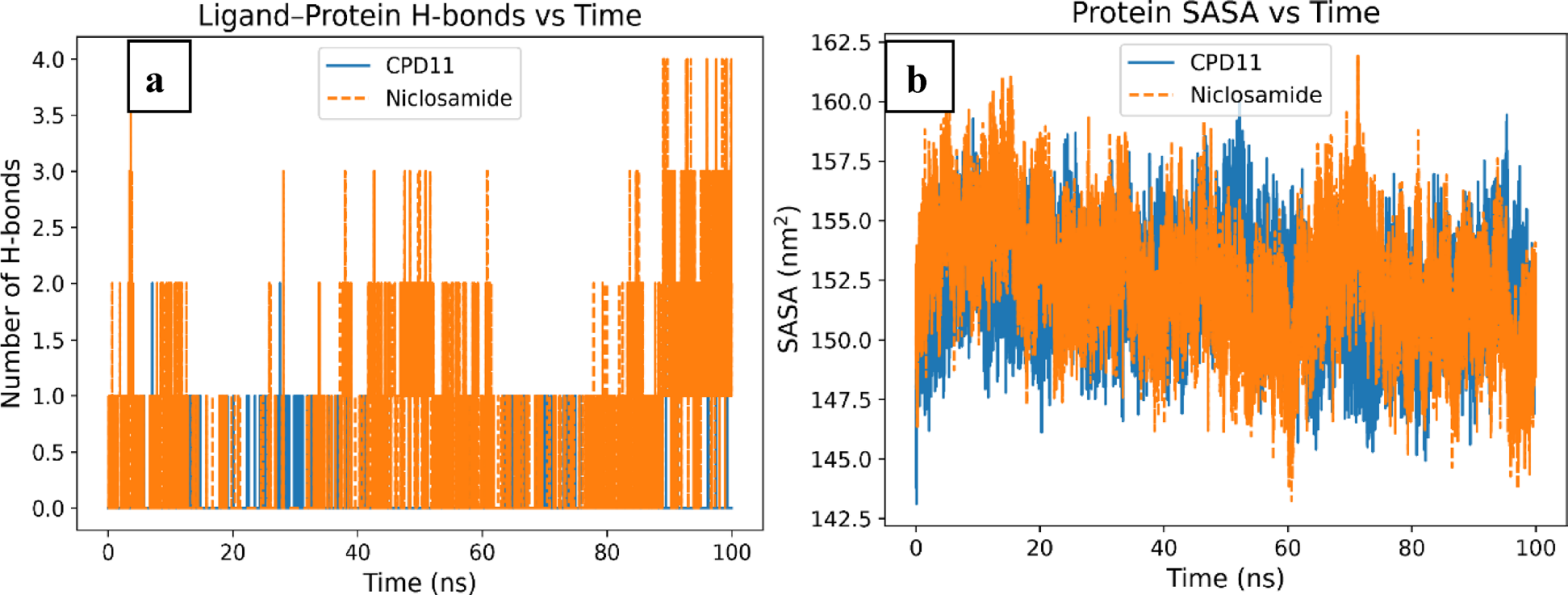
(**a**) Molecular dynamic simulation H-Bond variation, (**b**) Molecular dynamic simulation SASA variation.

The hydrogen bond analysis throughout the simulation trajectory and offered additional support for the docking results. Both compounds-maintained hydrogen bonding interactions with JAK1, but the pattern and consistency differed between the two ligands. Compound **11** consistently formed hydrogen bonds, reflecting the stable interactions predicted by docking, particularly with Leu959. The relatively stable hydrogen bond count for compound **11** throughout the simulation indicates that this key interaction is maintained, contributing to the overall binding stability. Meanwhile, NIC showed some fluctuation in hydrogen bond formation, consistent with its greater conformational flexibility observed in the ligand RMSD analysis. These findings demonstrate that the static docking predictions of hydrogen bonding patterns are validated by the dynamic simulation data, (Fig. 20a). The solvent-accessible surface area analysis provided deep details into the burial of the ligands within the JAK1 binding pocket. A decrease in SASA typically indicates that the ligand becomes more deeply embedded within the protein, shielded from the solvent, which is generally associated with stronger binding. Both ligands showed relatively stable SASA values after equilibration, but compound **11** exhibited a pattern consistent with deeper burial and more complete encapsulation by the protein structure. This observation aligns with its extensive hydrophobic interactions involving multiple leucine residues and aromatic contacts with phenylalanine and histidine residues. The reduced solvent exposure of compound **11** further explains its enhanced binding affinity, as desolvation is an energetically favorable process that contributes to the overall free energy of binding, (Fig. 20b).

Taken together, the molecular dynamics simulations not only confirm but also extend the insights gained from docking studies. While docking provided a snapshot of the most favorable binding poses and predicted interaction patterns, the MD simulations revealed that compound **11** maintains these interactions dynamically, with superior stability compared to NIC. The convergence of multiple analytical parameters protein RMSD, ligand RMSD, hydrogen bond persistence, and SASA paints a comprehensive picture: compound **11** forms a more stable, tightly bound complex with JAK1, characterized by a rich network of electrostatic, hydrophobic, and aromatic interactions.

## Conclusion

To enhance the anticancer properties of NIC, we synthesize thirteen schiff bases by combining them with different hydrophobic moieties. After that, every chemical is tested against the cell lines MCF-7, MDA-MB-231, PC-3, and WI38. It was discovered that, compounds **8** and **11** are the most selective cytotoxic agents. As a result, they were assessed further for a mechanistic analysis of JAK1 and CDK7. Remarkably, NIC (MCF-7, IC_50_ 0.22 ± 0.21, MDA-MB-231, IC_50_ 0.17 ± 0.05, PC-3, IC_50_ 0.20 ± 0.08 µM) was less effective JAK1 inhibitor than **8** (MCF-7, IC_50_ 0.017 ± 0.39, MDA-MB-231, IC_50_ 0.01 ± 0.11, PC-3, IC_50_ 0.10 ± 0.12 µM) and **11** (MCF-7, IC_50_ 0.05 ± 0.29, MDA-MB-231, IC_50_ 0.03 ± 0.15, PC-3, IC_50_ 0.04 ± 0.12 µM). Additionally, compound **11** produced a greater percentage of CDK7 inhibition than NIC, although compound **8** was similar. Interestingly, on the examined cancer cell lines, they demonstrated potential as dual inhibitors of JAK1 and CDK7. Likewise, these compounds ’ability to induce apoptosis is investigated as CDK7 inhibitors. They induce apoptosis by increasing the expression of caspases 1, 3, and 9 in MDA-MB-231 and PC-3 cells; however caspase 1 and 9 genes are not activated in the MCF-7 cell line. According to cell cycle analysis, compound **11** significantly increased the proportion of cells in the PreG1 phase by approximately 66.7 times and the percentage of cells in the G2/M phase by 1.67 times when compared to the control. Annexin V apoptosis assay revealed that, compound **11** was 8.15 times stronger apoptotic effect than the control trial in MCF-7 cells; it increases apoptotic cell death from 1.34 to 10.92% and necrotic cell death from 1.05 to 3.47%. *ADME* prediction demonstrated that most of our compounds are free of Lipiniski and Veber violations. Nearly, every compound exhibited improved absorption (79.51–87.72%) when taken orally, which is greater than NIC’s absorption (76.17%). Lastly, molecular docking of JAK1 and CDK7 verified our proposed mechanism of action. Our interesting candidates produced a binding pattern resembling the co-crystallized ligand with increased affinity owing to the aryl moiety of NIC-Schiff bases **8** and **11**. This suggests that compound **11** would likely show more potent and sustained JAK1 inhibition in biological systems, which could translate into improved therapeutic efficacy as an anticancer agent. Future directions will depend on the ongoing development of NIC by compromise between more hydrophobic groups with electron-rich substituents to Ar/R moiety of Schiff base along with innovative delivery mechanisms in order to address the issues related to bioavailability and solubility.

## Experimental section

Reagents and solvents were ordered from regular commercial suppliers and used without further purification. The reported yields apply to distilled products. All reactions were routinely checked with thin-layer chromatography (TLC) of Merck Silica Gel 60 F254 (0.25 mm thick) and visualization with a UV lamp. The melting points were measured in open capillary tubes using the Electrothermal LA 9000 SERIS, Digital Melting point Apparatus. IR spectra (KBr) were measured on a Shimadzu FT/IR 1650 (Perkin Elmer) spectrometer. ^1^H and ^13^C NMR spectra were recorded on JEOL JNM-ECZR Spectrometer at 500 MHz, and 13 C NMR spectra at 125 MHz at the National Research Institute in DMSO-*d*_*6.*_ Chemical shifts (*δ*) are reported in ppm relative to TMS as an internal standard. Mass Spectra were recorded at 70 EV on a DI-50 unit of Shimadzu GC/ MS-QP5050A Spectrometer at Regional Center for Mycology and Biotechnology, Al-Azhar University.

### *N*- (4-amino-2-chlorophenyl)-5-chloro-2-hydroxybenzamide (2)^24^

The reduction of nitro functionality of NIC to the corresponding amine, **2** involved the slow addition of zinc dust (0.46 g, 7.08 mmol) suspension in methanol to a methanolic mixture containing NIC (2 g, 5 mmol) and acetic acid (12.5 mL), followed by the addition of HCl drops. The reaction was concentrated to produce a buff solid powder *via* stirring it continuously until the effervescence vanished. Then the solid is recrystallized from absolute ethanol. m.p.(195–197 °C).

### General procedure for synthesis of compounds 3–15

A good yield of **3–10** can be obtained by adding a mixture of NIC-amine, 2 (0.01 mol), and different aldehydes (0.01 mol), such as 4-formylbenzonitrile, 4-(trifluoromethyl)benzaldehyde, 4-isopropylbenzaldehyde, 4-ethoxybenzaldehyde, 2-hydroxybenzaldehyde, 5-bromo-2-hydroxybenzaldehyde, 5-bromo-2-methoxybenzaldehyde, or 1-(4-bromophenyl)ethanone. The reaction took place under reflux for 4–8 h while ethanol (30 mL) and glacial acetic acid (1 mL) were present. In order to provide additional NIC-Schiff bases **11–15**, the same condensation reaction was performed using the following aldehydes: 1-phenyl-3-(thiophen-2-yl)-1 H-pyrazole-4-carbaldehyde, 2-naphthaldehyde, 2-hydroxy-1-naphthaldehyde, 2-chloro-7-ethoxyquinoline-3-carbaldehyde, or anthracene-9-carbaldehyde respectively. The completion of the reaction was monitored by TLC. The reaction mixture was allowed to cool down to room temperature and then poured into crushed ice. The precipitate was filtered, dried, and recrystallized from absolute ethanol.

### *(E)*-5-chloro-N-(2-chloro-4-((4-cyanobenzylidene)amino)phenyl)-2-hydroxybenzamide (3)

Light green powder, (yield 86%); m.p. (250-252oC); IR (KBr, *υ*/cm^-1^: 3433 (OH), 3158 (NH), 3050 (aromatic CH), 2229 (CN), 1631 (C=O of amide), 1576 (C=N); ^1^H NMR (500 MHz, in DMSO-*d*_6_) *δ* ppm: 12.23 (s, 1H, OH, D_2_O exchangeable), 10.89 (s, 1H, NH, D2O exchangeable), 8.76 (s, 1H, methine-H), 8.43 (d, *J* = 8.7 Hz, 1H, Ar-H), 8.04 (d, J = 8.3 Hz, 3H, Ar-Hs), 7.93 (d, *J* = 8.4 Hz, 2H, Ar-Hs), 7.56 (s, 1H, Ar-H), 7.45 (dd, *J* = 8.7, 2.8 Hz 1H, Ar-H), 7.35 (dd, *J* = 8.7 Hz, 2.3 Hz, 1H, Ar-H), 7.02 (d, *J* = 8.7 Hz, 1H, Ar-H); 13C NMR (125 MHz, in DMSO-d6) δ ppm: 162.89 (C=O), 159.88 (C=N), 155.76, 147.26, 140.17, 134.29, 133.82, 133.17 (2C), 130.35, 129.65 (2C), 124.26, 124.09, 123.20, 122.34, 121.91, 120.22, 119.50, 119.01, 113.99. M.S.(m/z %): 410 (M+., 55.25%) with a base peak at m/z: 254; Anal. Calc. for C21H13Cl2N3O2 (410.25): C, 61.48; H, 3.19; N, 10.24, Found C, 61.30; H, 3.20; N, 10.27 %.

### *(E)*-5-chloro-N-(2-chloro-4-((4-(trifluoromethyl)benzylidene)amino)phenyl)-2-hydroxy benzamide (4)

Light yellow powder, (yield 86%); m.p. (230–232 °C); IR (KBr, *υ*/cm^− 1^): s3205 (br-OH + NH), 3095 (aromatic CH), 2934 (aliphatic CH), 1643 (C = O of amide), 1559 (C = N); ^1^H NMR (500 MHz, in DMSO-*d*_*6*_) δ ppm: 10.96 (s, 1H, OH, D_2_O exchangeable), 10.36 (s, 1H, NH, D_2_O exchangeable), 8.81 (s, 1H, methine-H), 8.45 (d, *J* = 8.8 Hz, 1H, Ar-H), 8.11 (d, *J* = 8.1 Hz, 2 H, Ar-Hs), 7.96 (dd, *J* = 7.7, 4.9 Hz, 2 H, Ar-Hs), 7.86 (d, *J* = 8.2 Hz, 1H, Ar-Hs), 7.60 (s, 1H, Ar-H), 7.48 (dd, *J* = 8.7, 2.8 Hz 1H, Ar-H), 7.39 (dd, *J* = 8.7, 2.3 Hz 1H, Ar-H), 7.04 (d, *J* = 8.8 Hz, 1H, Ar-H); ^13^C NMR (125 MHz, in DMSO-*d*_*6*_) δ ppm: 163.19 (C = O), 160.53 (C = N), 156.33, 148.21, 147.83, 139.97, 134.07, 133.71, 130.77, 130.38 (2 C), 129.92, 126.30, 124.70, 124.03, 123.65, 122.33, 121.95, 120.24, 119.65, 113.97. Anal. Calc. for C_21_H_13_Cl_2_F_3_N_2_O_2_ (453.24): C, 55.65; H, 2.89; N, 6.18. Found: C, 55.45; H, 2.90; N, 6.20%.

### *(E)*-5-chloro-*N*-(2-chloro-4-((4-isopropylbenzylidene)amino)phenyl)-2-hydroxybenzamide (5)

Buff powder, (yield 73%); m.p. (240–242 °C); IR (KBr, *υ*/cm^− 1^): 3213 (br-OH + NH), 3091 (aromatic CH), 2985 (aliphatic CH), 1635 (C = O of amide), 1585 (C = N); ^1^H NMR (500 MHz, in DMSO-*d*_*6*_) δ ppm: 12.26 (s, 1H, OH, D_2_O exchangeable), 10.91 (s, 1H, NH, D_2_O exchangeable), 8.62 (s, 1H, methine-H), 8.38 (d, *J* = 8.7 Hz, 1H, Ar-H), 7.96 (s, 1H, Ar-H), 7.82 (d, *J* = 8.2 Hz, 1H, Ar-H), 7.51–7.46 (m, 3 H, Ar-Hs), 7.37 (d, *J* = 8.1 Hz, 2 H, Ar-Hs), 7.30 (dd, *J* = 8.8 Hz, 2.4 Hz 1H, Ar-H), 7.03 (d, *J* = 8.7 Hz, 1H, Ar-H), 3.01–2.87 (m, 1H, Ar-H), 1.20 (d, 6 H, Ar-Hs); ^13^C NMR (125 MHz, in DMSO-*d*_*6*_) δ ppm: 163.24 (C = O), 161.70 (C = N), 156.14, 153.07, 148.18, 134.27, 133.94, 133.33, 130.21, 129.50 (2 C), 127.36 (3 C), 124.78, 123.89, 122.07, 121.58, 119.16, 119.66, 39.79, 24.16 (2CH_3_). Anal. Calc. for C_23_H_20_Cl_2_N_2_O_2_ (426.09): C, 64.65; H, 4.72; N, 6.56. Found: C, 64.40; H, 4.73; N, 6.58%.

### *(E)*-5-chloro-*N*-(2-chloro-4-((4-ethoxybenzylidene)amino)phenyl)-2-hydroxybenzamide (6)

Buff powder, (yield 86%); m.p. (220–222 °C); IR (KBr, *υ*/cm^− 1^): 3201 (br-OH + NH), 3100 (aromatic CH), 2978 (aliphatic CH), 1635 (C = O of amide), 1604 (C = N); ^1^H NMR (500 MHz, in DMSO-*d*_*6*_) δ ppm: 10.95 (s, 1H, OH, D_2_O exchangeable), 10.40 (s, 1H, NH, D_2_O exchangeable), 8.56 (s, 1H, methine-H), 8.37 (d, J = 8.7 Hz, 1H, Ar-H), 7.96 (s, 1H, Ar-H), 7.82 (d, *J* = 8.6 Hz, 2 H, Ar-Hs), 7.49–7.43 (m, 2 H, Ar-Hs), 7.26 (dd, *J* = 8.7 Hz, 2 Hz, 1H, Ar-H), 7.07–7.01 (m, 3 H, Ar-Hs), 4.06 (q, 2 H, CH_2_), 1.30 (t, 3 H, CH_3_); ^13^C NMR (125 MHz, in DMSO-*d*_*6*_) δ ppm: 164.45 (C = O), 161.15 (C = N), 158.87, 157.05, 148.10, 137.21, 133.69, 132.42, 131.36, 130.18, 129.38, 127.27, 126.79, 123.26, 121.63, 119.66, 115.41, 115.32, 114.14, 113.76, 64.39, 15.33; Anal. Calc. for C_22_H_18_Cl_2_N_2_O_3_ (429.30): C, 61.55; H, 4.23; N, 6.53. Found: C, 61.35; H, 4.24; N, 6.55%.

### *(E)*-5-chloro-*N*-(2-chloro-4-((2-hydroxybenzylidene)amino)phenyl)-2-hydroxybenzamide (7)

Orange powder, (yield 86%); m.p. (270–272 °C); IR (KBr, *υ*/cm^− 1^): 3439 (2OH of phenol), 3285 (NH of amide), 3091 (aromatic CH), 1675 (C = O of amide), 1623 (C = N); ^1^H NMR (500 MHz, in DMSO-*d*_*6*_) δ ppm: 12.77 (s, 1H, OH, D_2_O exchangeable), 12.27 (s, 1H, OH, D_2_O exchangeable), 10.92 (s, 1H, NH, D_2_O exchangeable), 8.98 (s, 1H, methine-H), 8.45 (d, *J* = 8.7 Hz, 1H, Ar-H), 7.96 (s, 1H, Ar-H), 7.70 (s, 1H, Ar-H), 7.62 (d, *J* = 7.7 Hz, 1H, Ar-H), 7.50–7.44 (m, 2 H, Ar-Hs), 7.43–7.38 (m, 1H, Ar-H), 7.04 (d, *J* = 8.8 Hz, 1H, Ar-H), 6.96 (dd, *J* = 13.8 Hz, 7.8 Hz, 2 H, Ar-Hs); ^13^C NMR (125 MHz, in DMSO-*d*_*6*_) δ ppm: 164.25 (C = O), 163.16(C = N), 160.76, 155.98, 145.38, 134.22, 134.01, 133.11(2 C), 130.30, 124.74, 124.07, 123.68, 122.48, 121.91, 120.19, 119.79 (2 C), 119.64, 117.17; Anal. Calc. for C_20_H_14_Cl_2_N_2_O_3_ (401.24): C, 59.87; H, 3.52; N, 6.98 Found: C, 59.65; H, 3.50; N, 6.95.

### *(E)-N*-(4-((5-bromo-2-hydroxybenzylidene)amino)-2-chlorophenyl)-5-chloro-2-hydroxy benzamide (8)

Yellow powder, (yield 86%); m.p. (290–292 °C); IR (KBr, *υ*/cm^− 1^): 3410 (NH-amide), 3410, 3255, 3224 (2OH, NH), 3100 (aromatic CH), 1643 (C = O of amide), 1620 (C = N); ^1^H NMR (500 MHz, in DMSO-*d*_*6*_) δ ppm: 12.70 (s, 1H, OH, D_2_O exchangeable), 12.55 (s, 1H, OH, D_2_O exchangeable), 11.26 (s, 1H, NH, D_2_O exchangeable), 8.95 (s, 1H, methine-H), 8.49 (d, *J* = 8.6 Hz, 1H, Ar-H), 7.94 (s, 1H, Ar-H), 7.83 (s, 1H, Ar-H), 7.68 (s, 1H, Ar-H), 7.52 (d, *J* = 8.1 Hz, 1H, Ar-H), 7.43 (d, *J* = 7.9 Hz, 2 H, Ar-Hs), 7.01 (d, *J* = 8.8 Hz, 1H, Ar-H), 6.92 (d, *J* = 8.5 Hz, 1H, Ar-H); ^13^C NMR (125 MHz, in DMSO-*d*_*6*_) δ ppm: 163.0 (C = O), 152.42 (C = N), 159.73, 156.09, 145.10, 136.16, 123.55, 123.96, 124.60, 134.40, 133.98, 130.30 (2 C), 122.60, 121.94 (2 C), 121.82, 120.19, 119.63, 110.55; Mass spectrometry spectrum (m/z %): 479.88 (M^+.^, 55.25%) with a base peak at m/z: 154.9; Anal. Calc. for C_20_H_13_BrCl_2_N_2_O_3_ (480.14): C, 50.03; H, 2.73; N, 5.83 Found: C, 50.23; H, 2.71; N, 5.80%.

### *(E)-N*-(4-((5-Bromo-2-methoxybenzylidene)amino)-2-chlorophenyl)-5-chloro-2-hydroxy benzamide (9)

Pale yellow powder, (yield 86%); m.p. (245–247 °C); IR (KBr, *υ*/cm^− 1^): 3224, 3167 (br-OH + NH), 3109 (aromatic CH), 2978 (aliphatic CH), 1651 (C = O of amide), 1604 (C = N); ^1^H NMR (500 MHz, in DMSO-*d*_*6*_) δ ppm: 12.30 (s, 1H, OH, D_2_O exchangeable), 10.96 (s, 1H, NH, D_2_O exchangeable), 8.95 (s, 1H, methine-H), 8.49 (d, *J* = 8.6 Hz, 1H, Ar-H), 7.94 (s, 1H, Ar-H, Ar-H), 7.83 (s, 1H, Ar-H), 7.68 (s, 1H, Ar-H), 7.46 (s, 1H, Ar-H), 7.52 (d, *J* = 8.1 Hz 1H, Ar-H), 7.44 (d, *J* = 7.9 Hz 1H, Ar-H), 7.01 (d, *J* = 8.8 Hz, 1H, Ar-H), 6.92 (d, *J* = 8.5 Hz, 1H, Ar-H), 3.47 (s, 3 H, CH_3_); ^13^C NMR (125 MHz, in DMSO-*d*_*6*_) δ ppm: 159.08 (C = O), 157.22 (C = N), 156.05, 155.14, 148.49, 136.02 (2 C), 130.43, 130.26 (2 C), 129.46, 127.02, 126.28, 123.99, 123.76, 122.52, 121.41, 119.66 (2 C), 109.41, 56.81; Anal. Calc. for C_21_H_15_Br_2_Cl_2_N_2_O_3_ (494.17): C, 51.04; H, 3.06; N, 5.67 Found: C, 51.23; H, 3.04; N, 5.64%.

### *(E)-N*-(4-((1-(4-Bromophenyl)ethylidene)amino)-2-chlorophenyl)-5-chloro-2-hydroxybenzamide (10)

Buff powder, (yield 86%); m.p. (140–142 °C); IR (KBr, *υ*/cm^− 1^): 3290, 3240 (br-OH + NH), 3078 (aromatic CH), 2905 (aliphatic CH), 1631 (C = O of amide), 1604 (C = N); ^1^H NMR (500 MHz, in DMSO-*d*_*6*_) δ ppm: 12.23 (s, 1H, OH, D_2_O exchangeable), 10.37 (s, 1H, NH, D_2_O exchangeable), 7.97 (s, 1H, Ar-H), 7.84 (d, *J* = 8.5 Hz, 2 H, Ar-Hs), 7.69 (d, *J* = 8.5 Hz, 2 H, Ar-Hs), 7.62 (d, *J* = 8.7 Hz, 1H, Ar-H), 7.44 (dd, *J* = 8.8 Hz, 2.7 Hz, 1H, Ar-H), 6.98 (d, *J* = 8.7, Hz, 1H, Ar-H), 6.68 (s, 1H, Ar-H), 6.51 (d, *J* = 8.7 Hz, 1H, Ar-H), 2.54 (s, 3 H, CH_3_); ^13^C NMR (125 MHz, in DMSO-*d*_*6*_) δ ppm: 164.38 (C = O), 162.19 (C = N), 157.05, 147.92, 145.44, 144.64, 136.37, 133.69, 132.33, 130.71, 129.39, 127.83, 127.38, 126.82, 123.60, 123.31, 121.64, 119.74, 114.16, 113.50, 27.64; Anal. Calc. for C_21_H_15_BrCl_2_N_2_O_2_ (478.17): C, 52.75; H, 3.16; N, 5.86 Found: C, 52.95; H, 3.14; N, 5.83%.

### *(E)*-5-chloro-*N*-(2-chloro-4-(((1-phenyl-3-(thiophen-2-*yl*)-1*H*-pyrazol-4-*yl*)methylene)amino) phenyl)-2-hydroxybenzamide (11)

Greenish yellow powder, (yield 86%); m.p. (220–222 °C); IR (KBr, *υ*/cm^− 1^): 3421, 3383 (OH + NH), 3070 (aromatic CH), 1663 (C = O of amide), 1581 (C = N); ^1^H NMR (500 MHz, in DMSO-*d*_*6*_) δ ppm: 12.31 (s, 1H, OH, D_2_O exchangeable), 10.89 (s, 1H, NH, D_2_O exchangeable), 9.14 (s, 1H, methine-H), 8.71 (s, 1H, Ar-H), 8.38 (d, *J* = 8.7 Hz, 1H, Ar-H), 8.01–7.94 (m, 4 H, Ar-Hs), 7.66–7.64 (m, 1H, Ar-H), 7.57–7.50 (m, 3 H, Ar-Hs), 7.48 (dd, *J* = 8.7 Hz, 2.8 Hz, 1H, Ar-H), 7.40–7.30 (m, 2 H, Ar-Hs), 7.18 (dd, *J* = 5.0 Hz, 3.7 Hz, 1H, Ar-H), 7.04 (d, *J* = 8.8 Hz, 1H, Ar-H); ^13^C NMR (125 MHz, in DMSO-*d*_*6*_) δ ppm: 163.26 (C = O), 156.15, 153.51 (2 C = N), 149.15, 147.23, 139.17, 134.46, 133.93, 132.24, 131.71, 130.23 (3 C), 128.95, 128.56 (2 C), 127.85, 124.87, 124.01, 121.98, 121.38, 120.17, 119.98, 119.66, 119.43 (2 C), 100.0 (pyrazole-C). Mass spectrometry spectrum (m/z %): 532 (M^+.^, 0.45%) with a base peak at m/z: 142; Anal. Calc. for C_27_H_18_Cl_2_N_4_O_2_S (532.43): C, 60.79; H, 3.40; N, 10.50. Found: C, 60.55; H, 3.41; N, 10.54.

### *(E)*-5-chloro-*N*-(2-chloro-4-((naphthalen-2-ylmethylene)amino) phenyl)-2-hydroxybenzamide (12)

Yellow powder, (yield 86%); m.p. (255–257 °C); IR (KBr, *υ*/cm^− 1^): 3201, 3163 (br-OH + NH), 3062 (aromatic CH), 1620 (C = O of amide), 1593 (C = N); ^1^H NMR (500 MHz, in DMSO-*d*_*6*_) δ ppm: 12.30 (s, 1H, OH, D_2_O exchangeable), 10.96 (s, 1H, NH, D_2_O exchangeable), 8.84 (s, 1H, methine-H), 8.43 (d, *J* = 8.7 Hz, 1H, Ar-H), 8.39 (s, 1H, Ar-H), 8.10 (dd, *J* = 8.6 Hz, 1.4 Hz 1H, Ar-H), 8.06–7.96 (m, 4 H, Ar-Hs), 7.63–7.56 (m, 3 H, Ar-Hs), 7.48 (dd, *J* = 8.8, 2.8 Hz, 1H, Ar-H), 7.48 (dd, *J* = 8.7 Hz, 2.4 Hz, 1H, Ar-H), 7.04 (d, *J* = 8.7 Hz, 1H, Ar-H); ^13^C NMR (125 MHz, in DMSO-*d*_*6*_) δ ppm: 161.76 (C = O), 149.31 (C = N), 148.50, 146.35, 135.13, 133.91, 133.68, 133.39, 132.12, 130.20 (2 C), 129.36, 129.28 (2 C), 129.08, 128.42 (3 C), 127.45, 124.09, 123.83, 122.21, 121.70, 119.81; Anal. Calc. for C_24_H_16_Cl_2_N_2_O_2_ (435.30): C, 66.22; H, 3.70; N, 6.44 Found: C, 66.40; H, 3.68; N, 6.41%.

### *(E)*-5-chloro-*N*-(2-chloro-4-(((2-hydroxynaphthalen-1-*yl*)methylene)amino)phenyl)-2-hydroxy benzamide (13)

Orange red powder, (yield 73%); m.p. (325–327 °C); IR (KBr, *υ*/cm^− 1^): 3425 (2OH), 3271 (NH), 3070 (aromatic CH), 1624 (C = O of amide), 1585 (C = N); ^1^H NMR (500 MHz, in DMSO-*d*_*6*_) δ ppm: 15.53 (s, 1H, OH, D_2_O exchangeable), 12.27 (s, 1H, OH, D_2_O exchangeable), 10.94 (s, 1H, NH, D_2_O exchangeable), 9.69 (s, 1H, methine-H), 8.52 (dd, *J* = 11.9 Hz, 8.6 Hz, 2 H, Ar-Hs), 8.02–7.90 (m, 3 H, Ar-Hs), 7.79 (d, *J* = 7.9 Hz, 1H, Ar-H), 7.67–7.45 (m, 3 H, Ar-Hs), 7.39–7.32 (m, 1H, Ar-H), 7.04 (t, *J* = 9.4 Hz, 2 H, Ar-Hs); ^13^C NMR (125 MHz, in DMSO-*d*_*6*_) δ ppm: 168.83 (C = O), 164.92, 163.12 (C = N), 157.47, 142.25, 137.16, 134.01, 133.89 (2 C), 130.29 (2 C), 129.54, 128.59, 124.18 (2 C), 123.79, 121.83, 121.66 (2 C), 121.55, 121.26, 120.20, 119.69 (2 C); Anal. Calc. for C_24_H_16_Cl_2_N_2_O_3_ (451.30): C, 63.87; H, 3.57; N, 6.21 Found: C, 63.65; H, 3.62; N, 6.23%.

### *(E)*-5-chloro-*N*-(2-chloro-4-(((2-chloro-7-ethoxyquinolin-3-*yl*)methylene)amino)phenyl)-2-hydroxybenzamide (14)

Reddish brown powder, (yield 86%); m.p. (225–227 °C); IR (KBr, *υ*/cm^− 1^): 3213, 3159 (br-OH + NH), 3105 (aromatic CH), 2981 (aliphatic CH), 1643 (C = O of amide), 1579 (C = N); ^1^H NMR (500 MHz, in DMSO-*d*_*6*_) δ ppm: 12.25 (s, 1H, OH, D_2_O exchangeable), 10.90 (s, 1H, NH, D_2_O exchangeable), 9.01 (s, 1H, methine-H), 8.90 (s, 1H, Ar-H), 8.44 (d, *J* = 8.8 Hz, 1H, Ar-H), 8.11 (d,, 1H, Ar-H), 7.96 (s, 1H, Ar-H), 7.59 (s, 1H, Ar-H), 7.47 (dd, *J* = 8.7 Hz, 2.8 Hz, 1H, Ar-H), 7.39 (dd, *J* = 8.7 Hz, 2.3 Hz, 1H, Ar-Hs), 7.35 (s, 1H, Ar-H), 7.30 (dd, *J* = 8.9 Hz, 2.5 Hz, 1H, Ar-Hs), 7.04 (d, *J* = 8.7 Hz, 1H, Ar-H), 4.20 (q, 2 H, CH_2_), 1.38 (t, 3 H, CH_3_); ^13^C NMR (125 MHz, in DMSO-*d*_*6*_) δ ppm: 162.99 (C = O), 162.48, 156.61 (C = N), 155.90, 150.58, 150.46 (quinoline C = N), 148.09, 141.05, 137.90, 133.88, 131.19, 130.29 (2 C), 126.29, 124.02, 122.33, 121.70, 121.34, 119.58, 113.98, 107.72, 107.46, 98.76, 64.74, 14.70. Mass spectrometry spectrum (m/z %): 513 (M^+.^, 1.54%) with a base peak at m/z: 154.9; Anal. Calc. for C_25_H_18_Cl_3_N_3_O_3_ (513.79): C, 58.33; H, 3.52; N, 8.16 Found: C, 58.55; H, 3.50; N, 8.12%.

### *(E)-N*-(4-((anthracen-9-ylmethylene)amino)-2-chlorophenyl)-5-chloro-2-hydroxybenzamide (15)

Orange powder, (yield 86%); m.p. (260–262 °C); IR (KBr, *υ*/cm^− 1^): 3363 (OH), 3302 (NH), 3078 (aromatic CH), 1639 (C = O of amide), 1581 (C = N); ^1^H NMR (500 MHz, in DMSO-*d*_*6*_) δ ppm: 12.30 (s, 1H, OH, D_2_O exchangeable), 10.97 (s, 1H, NH, D_2_O exchangeable), 9.84 (s, 1H, methine-H), 8.86 (d, *J* = 8.9 Hz, 2 H, Ar-Hs), 8.79 (s, 1H, Ar-H), 8.48 (d, *J* = 8.7 Hz, 1H, Ar-H), 8.15 (d, *J* = 8.3 Hz, 2 H, Ar-Hs), 8.0 (s, 1H, Ar-H), 7.79 (s, 1H, Ar-H), 7.65–7.54 (m, 4 H, Ar-Hs), 7.50 (dd, *J* = 8.8 Hz, 2.8 Hz, 1H, Ar-H), 7.06 (d, *J* = 8.8 Hz, 2 H, Ar-Hs); ^13^C NMR (125 MHz, in DMSO-*d*_*6*_) δ ppm: 163.32 (C = O), 161.17 (C = N), 156.10, 149.18, 133.99, 133.74, 131.48, 130.27, 129.89, 129.53 (2 C), 128.33, 128.05 (2 C), 127.22, 126.36, 126.17 (2 C), 125.57, 124.91, 124.91, 124.45, 124.04, 123.94, 122.35, 121.86, 120.20, 119.67; Mass spectrometry spectrum (m/z %): 485 (M^+.^, 1.53%) with a base peak at m/z: 142. Anal. Calc. for C_28_H_18_C_l2_N_2_O_2_ (485.36): C, 69.29; H, 3.74; N, 5.77 Found: C, 69.55; H, 3.72; N, 5.74%.

#### Cytotoxic evaluation

Regarding the origin of the cancer cell lines used in the study, ATCC provided the human lung fibroblast (WI38) cell line, two breast cancer cell lines (MCF-7), (MDA-MB-231) and prostate cancer cell line (PC-3), through VACSERA, Cairo, Egypt. This colorimetric assay is based on the conversion of the yellow tetrazolium bromide (MTT) to a purple formazan derivative by mitochondrial succinate dehydrogenase in viable cells. Cell lines were cultured in RPMI-1640 medium with 10% fetal bovine serum. Antibiotics added were 100 units/ml penicillin and 100 µg/ml streptomycin at 37 C in a 5% CO_2_ incubator. The cell lines were seeded in a 96-well plate at a density of 1.0 × 10 4 cells/ well at 37 °C for 48 h under 5% CO_2_. After incubation the cells were treated with different concentration of compounds and incubated for 24 h. After 24 h of drug treatment, 20 µl of MTT solution at 5 mg/ml was added and incubated for 4 h. Dimethyl sulfoxide (DMSO) in volume of 100 µl is added into each well to dissolve the purple formazan formed. The colorimetric assay is measured and recorded at absorbance of 570 nm using a plate reader (EXL 800, USA). The relative cell viability in percentage was calculated as (A570 of treated samples/A570 of untreated sample) X 100.

#### Evaluation of JAK1 inhibitory activity^39^

Human Jak1 ELISA Novus Kit (Colorimetric) NBP2-80249; the Detection Range: 0.16-10 ng/mL.

#### Evaluation of CDK7 inhibitory activity^40^

 Human Cyclin-dependent Kinase 7, CDK-7 ELISA Kit, E0951Hu; the Standard Curve Range 0.05-20ng/mL.

##### Cell cycle analysis

FACS Caliber flow cytometer was utilized to determine the effect of **11** on the cell cycle of breast tumour MCF-7 guided by the previously reported method^24^

####  Effect of 8 and 11 on active caspase-1, -3 and 9 levels

Caspase-3 is evaluated by using the reported method^42,43^. Elisa assay was used to determine the concentrations of human caspases-1, -3, and − 9. Human CASP1(Caspase 1) ELISA Kit, Catalog No : E-EL-H0016, Human CASP3(Caspase 3) ELISA Kit, Catalog No : E-EL-H0017 and Human Caspase 9 ELISA Kit, Catalog Number NBP1-83734.

#### Annexin-V-FITC apoptosis assay^46^

The apoptosis of **11** was detected through Annexin V-FITC/PI apoptosis detection kit utilizing FACS Caliber flow cytometer following the reported procedure.

## Supplementary Information

Below is the link to the electronic supplementary material.

Supplementary Material 1

Supplementary Material 2

## Data Availability

Chemistry data are submitted as Supplementary material S1 and IC 50 data are submitted as Supplementary material S2.
