# Supplementary material for: Novel niclosamide-derived Schiff bases as a dual-targeted anticancer agents
Source: Sci Rep. 2026 Jan 13;16:1959. doi: 10.1038/s41598-025-33185-2 (PMC12804884; doi:10.1038/s41598-025-33185-2)
Supplement: Supplementary file 1 — Supplementary Material 1 [file 41598_2025_33185_MOESM1_ESM.pdf]

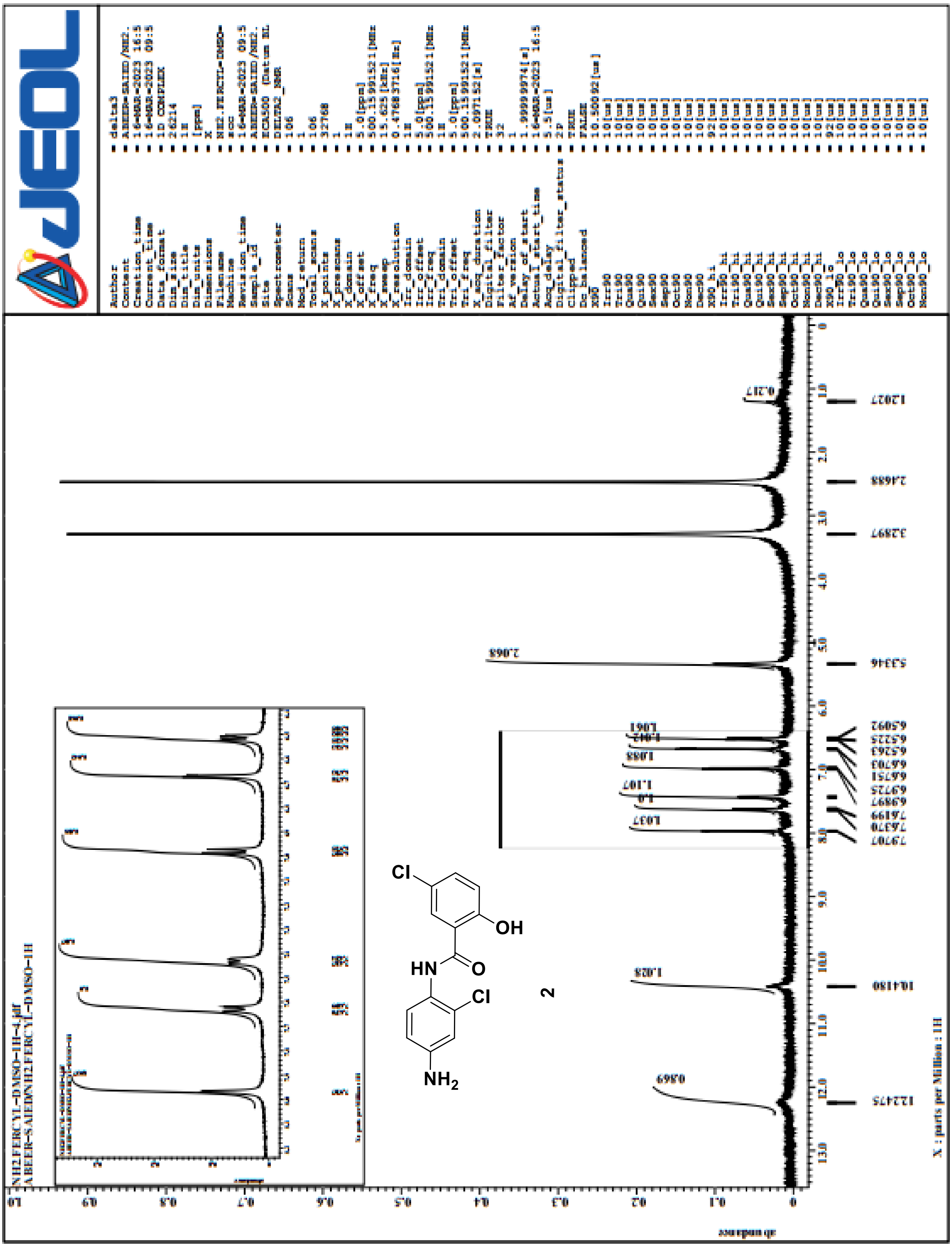

Appendix (1a): ^{1}H NMR spectrum of **compound 2**

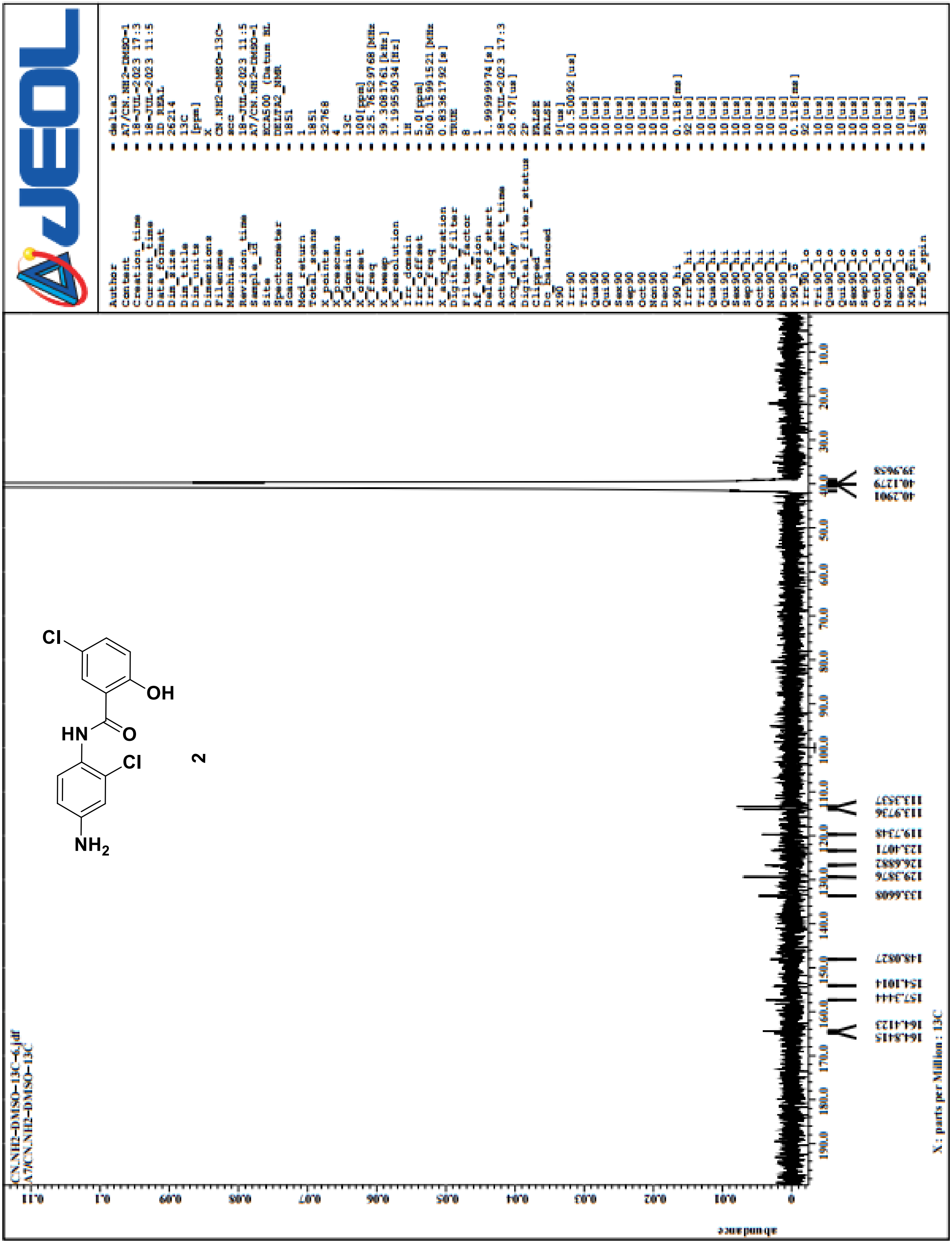

Appendix (1b): ^{13}C NMR spectrum of **compound 2**

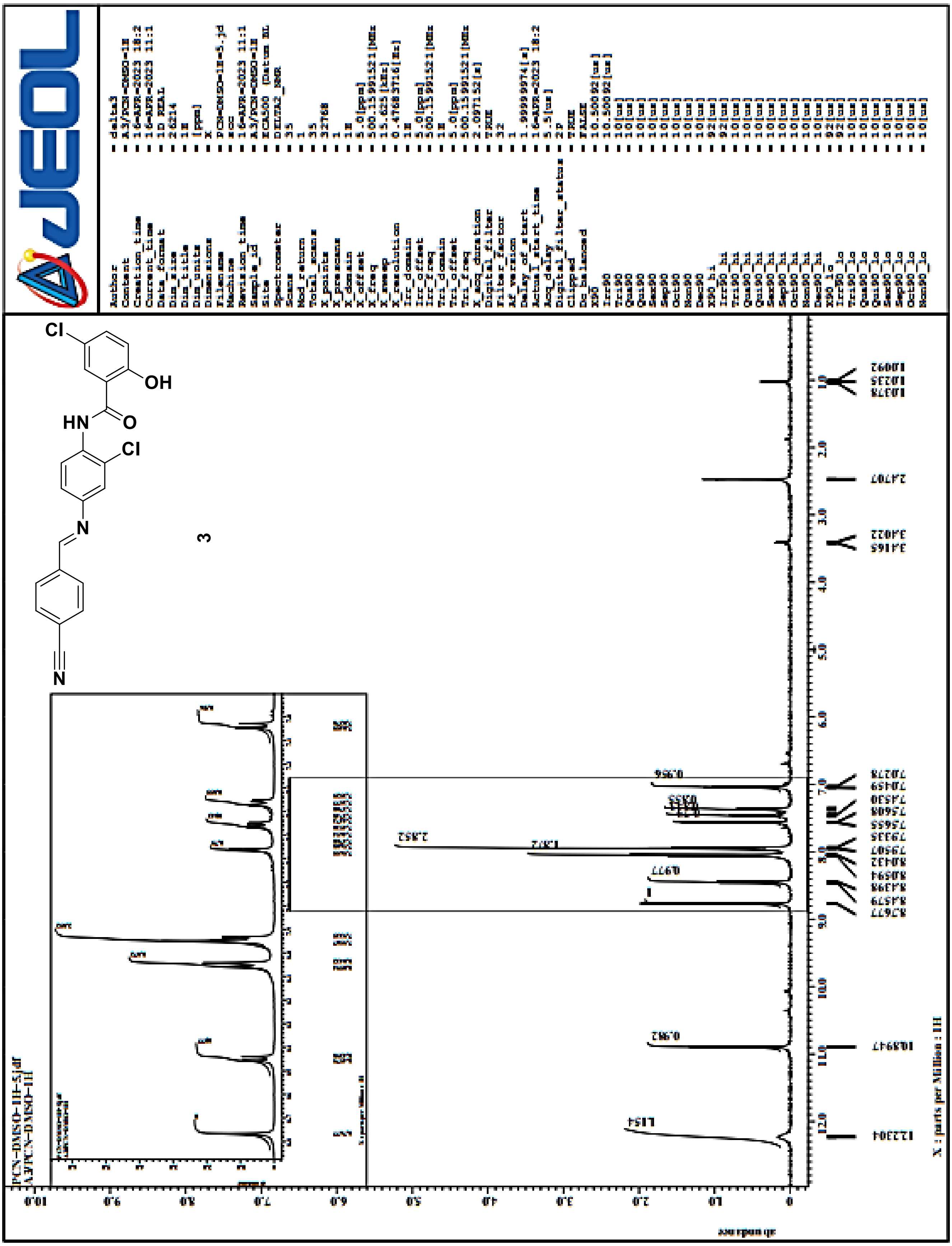

Appendix (2a): ^{1}H NMR spectrum of **compound 3**

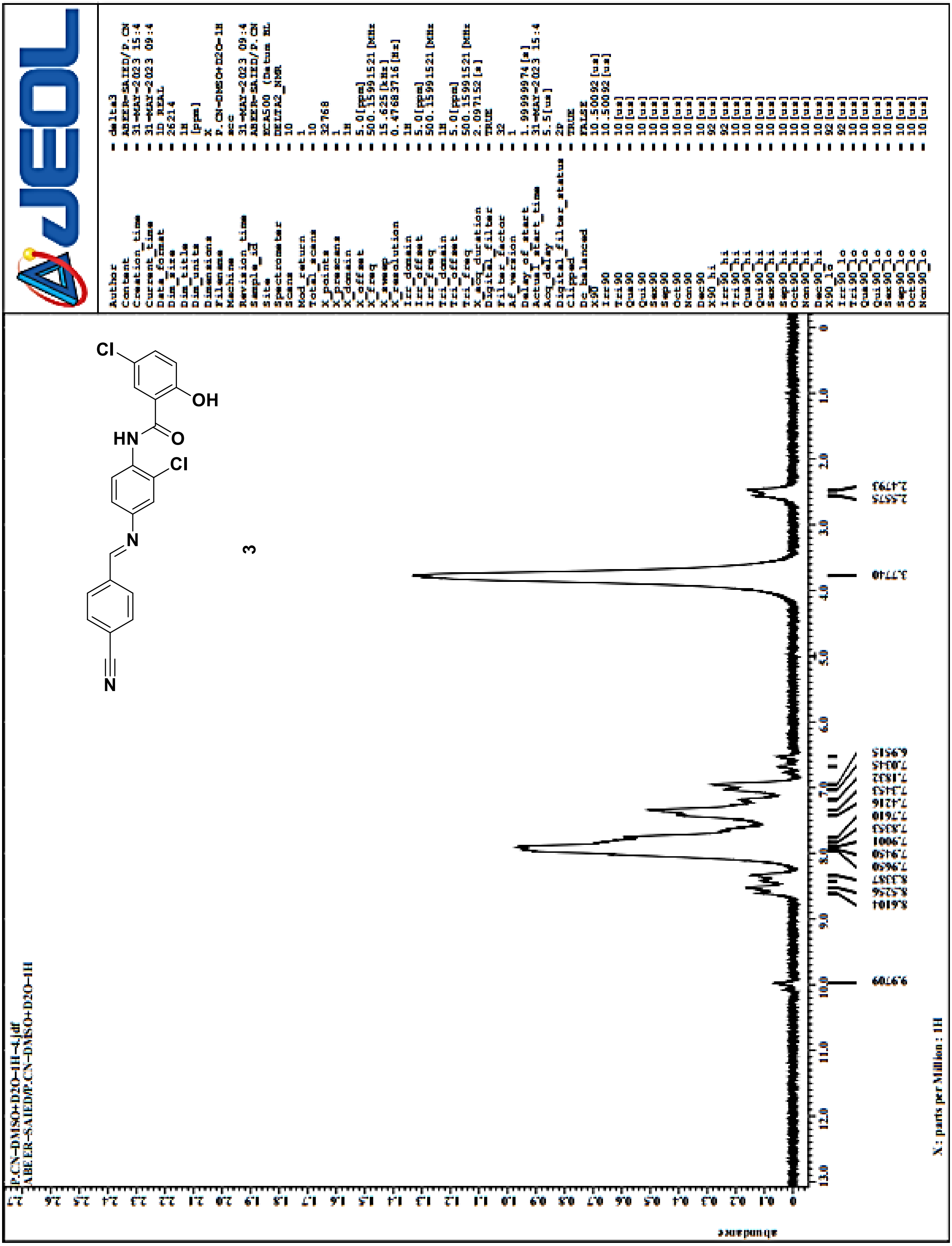

Appendix (2b): ^{1}H NMR spectrum of **compound 3 (D_2O)**

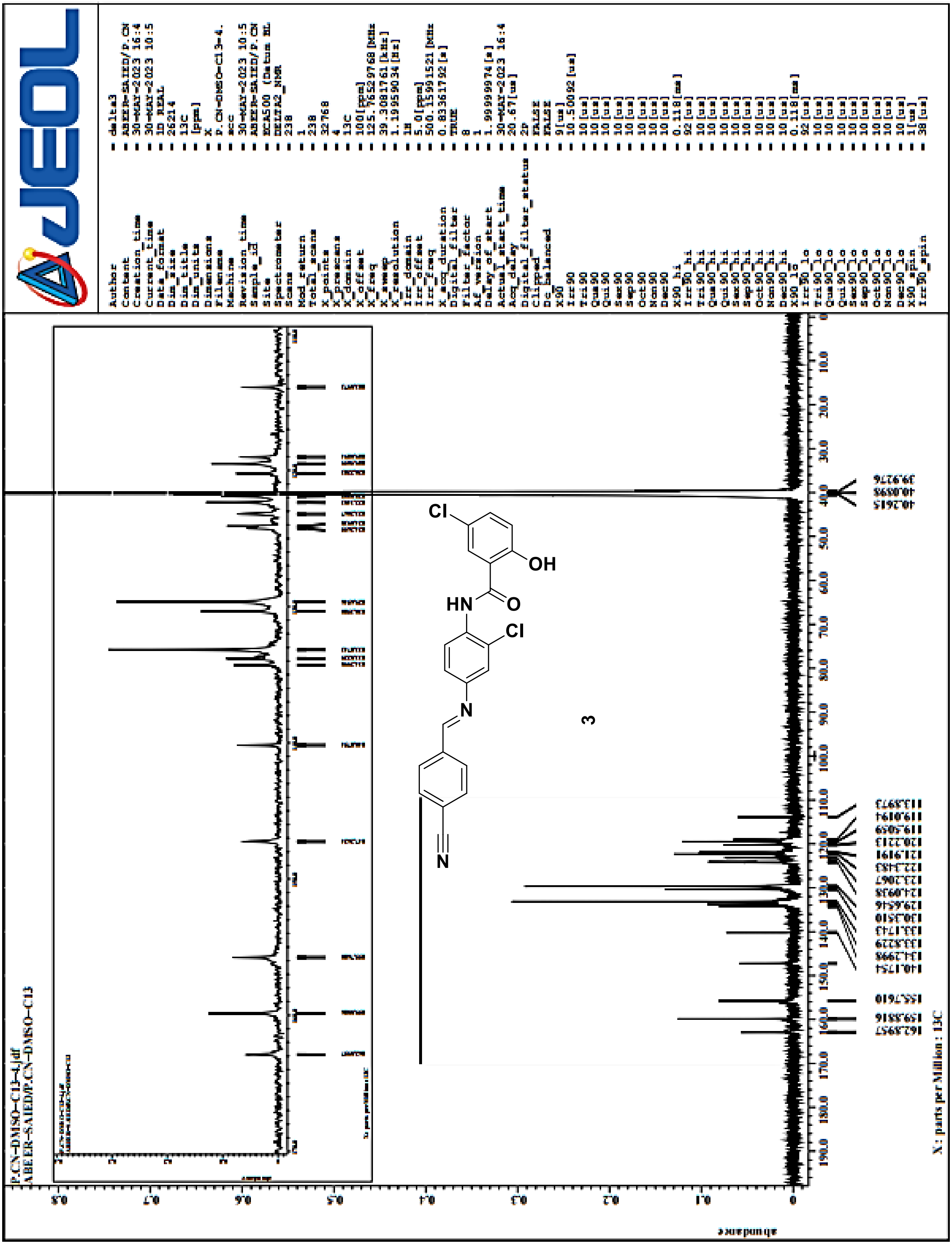

Appendix (2c): ^{13}C NMR spectrum of **compound 3**

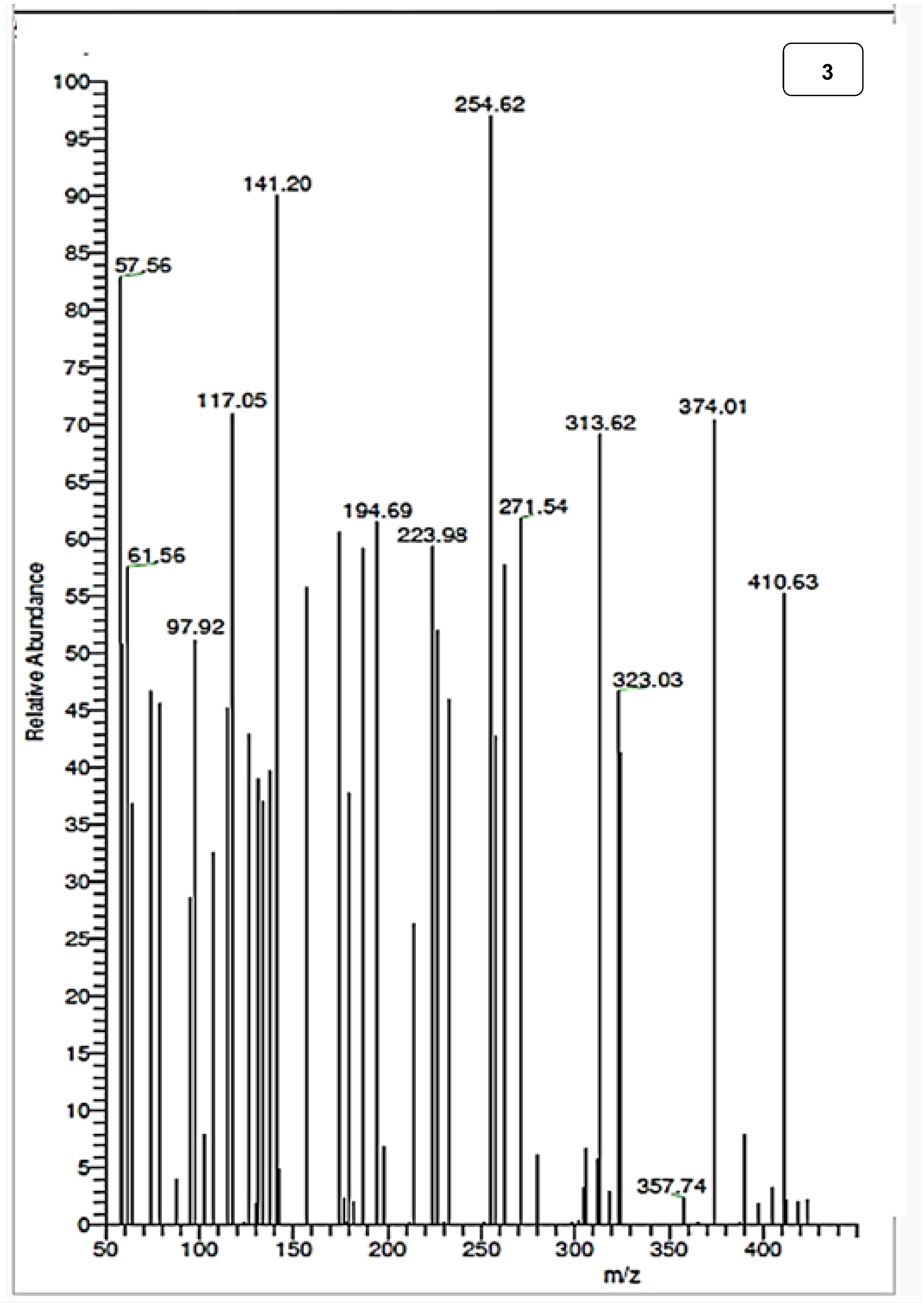

Appendix (2d): Mass spectrum of **compound 3**

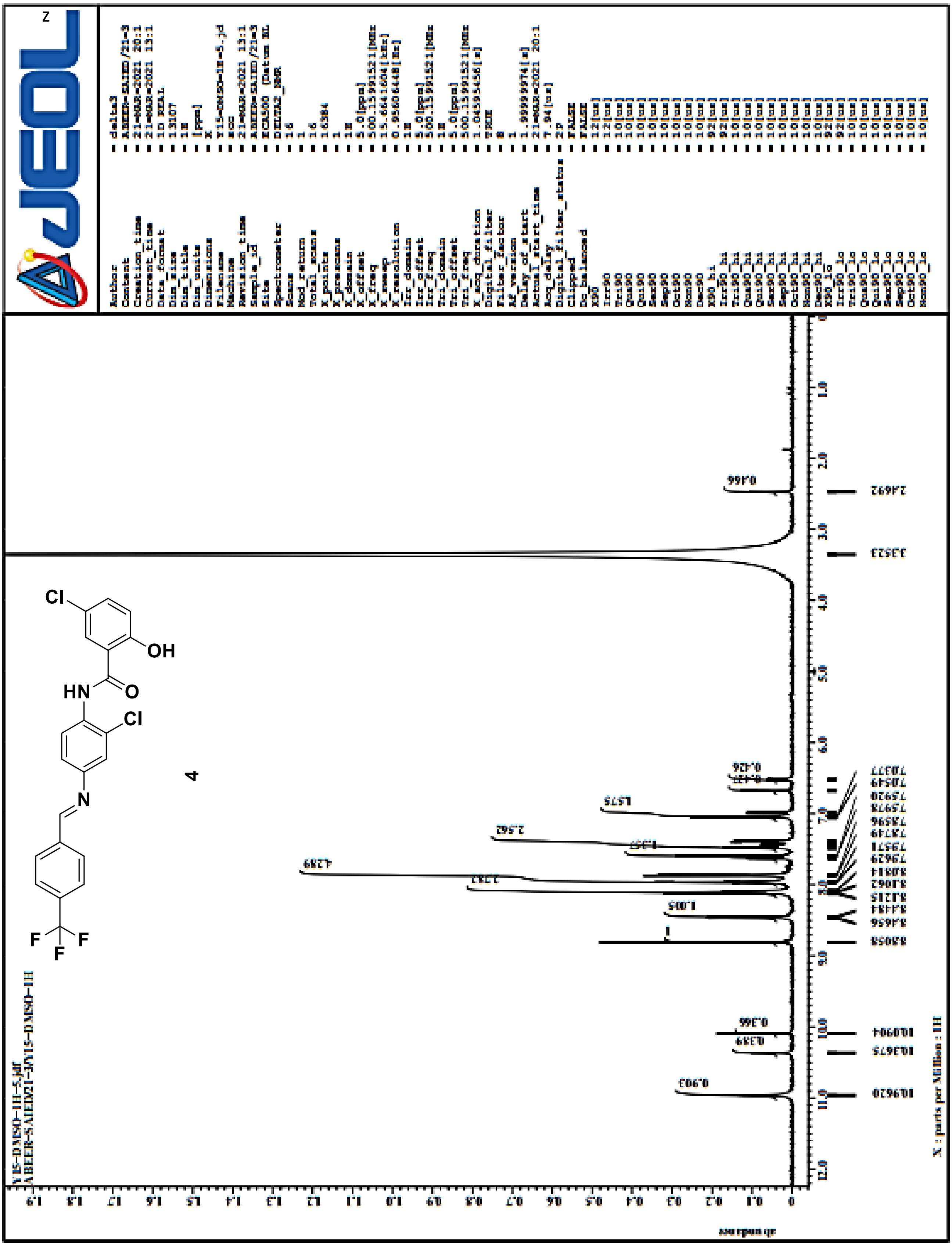

Appendix (3a): ^{1}H NMR spectrum of **compound 4**

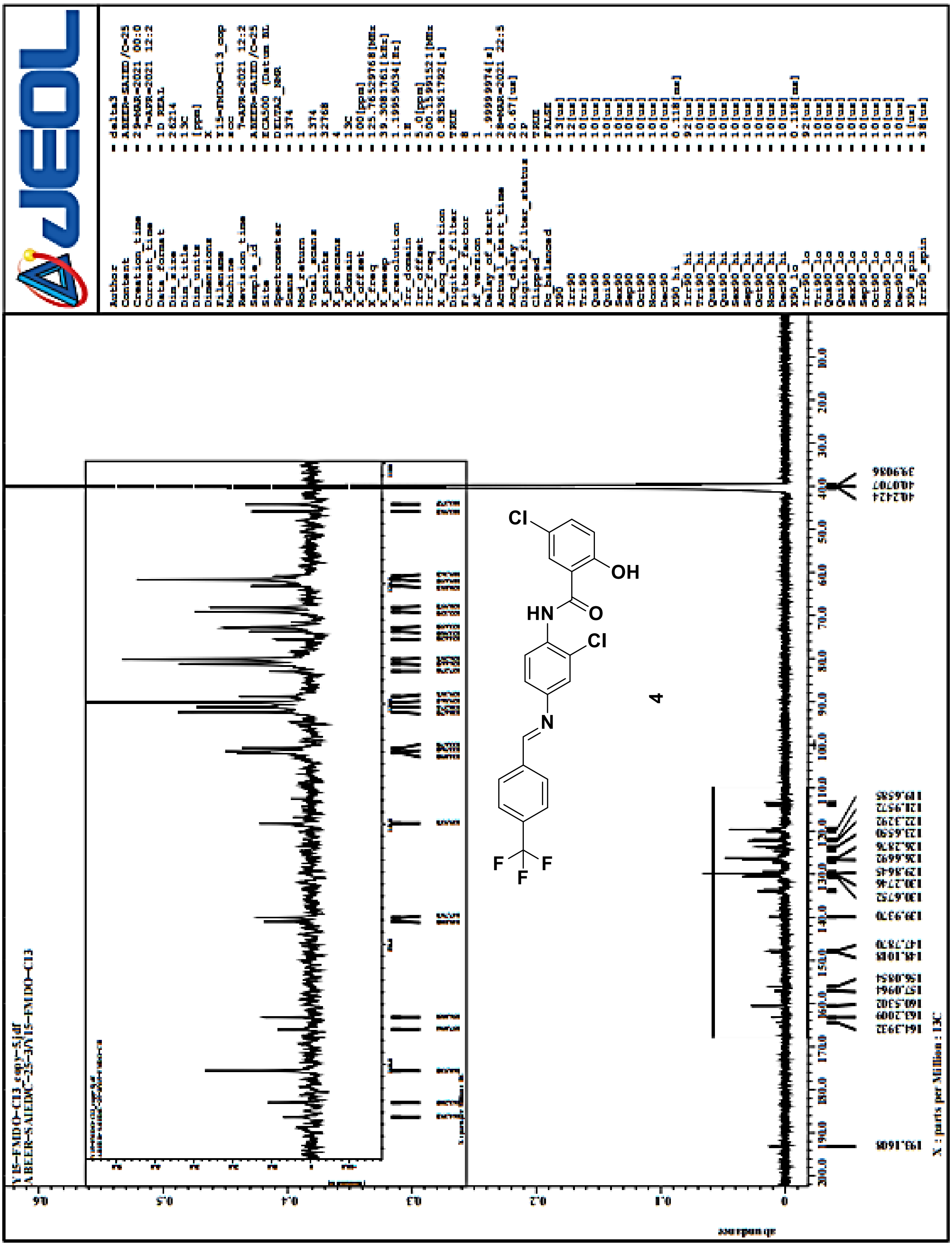

Appendix (3b): ^{13}C NMR spectrum of **compound 4**

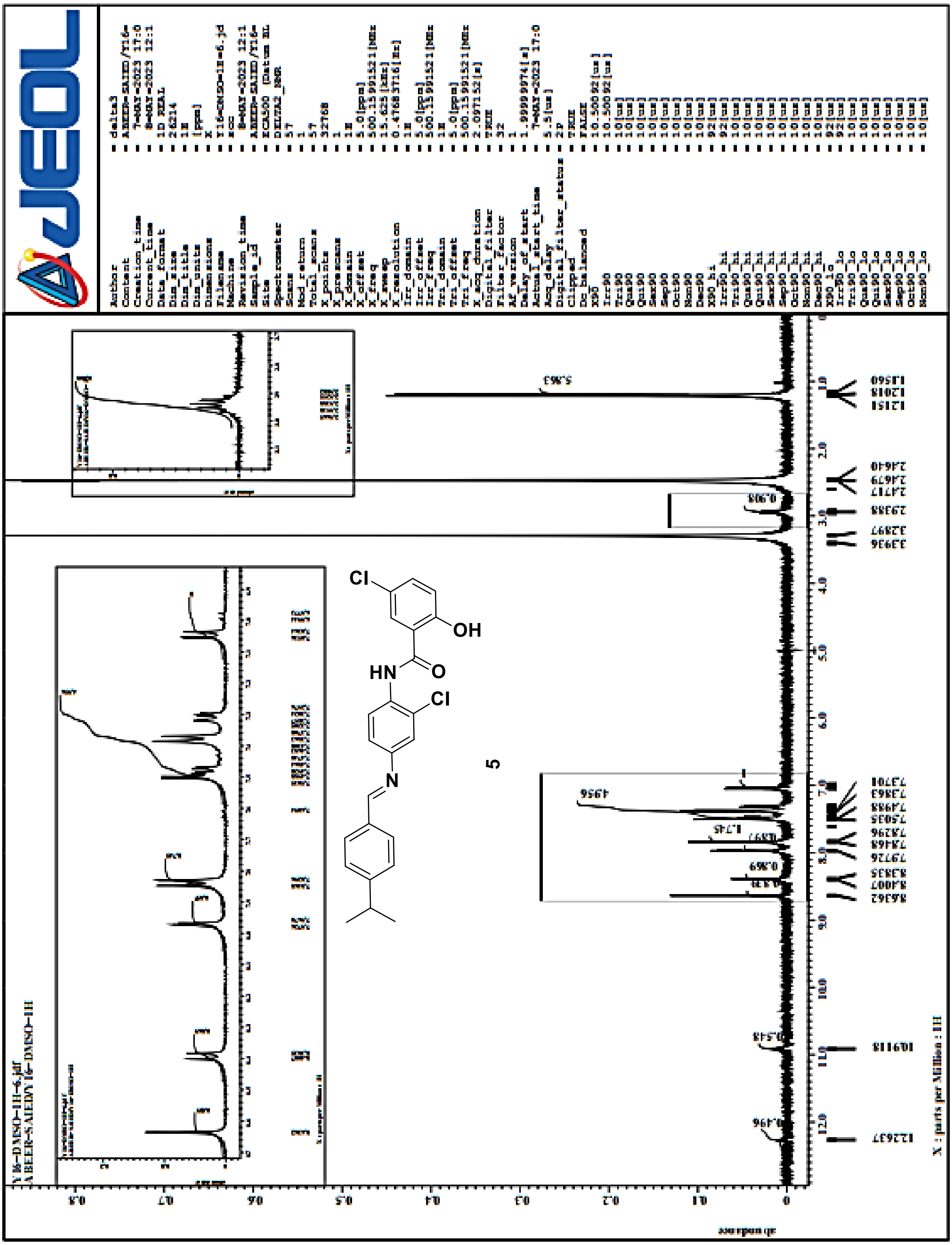

Appendix (4a): ^{1}H NMR spectrum of **compound 5**

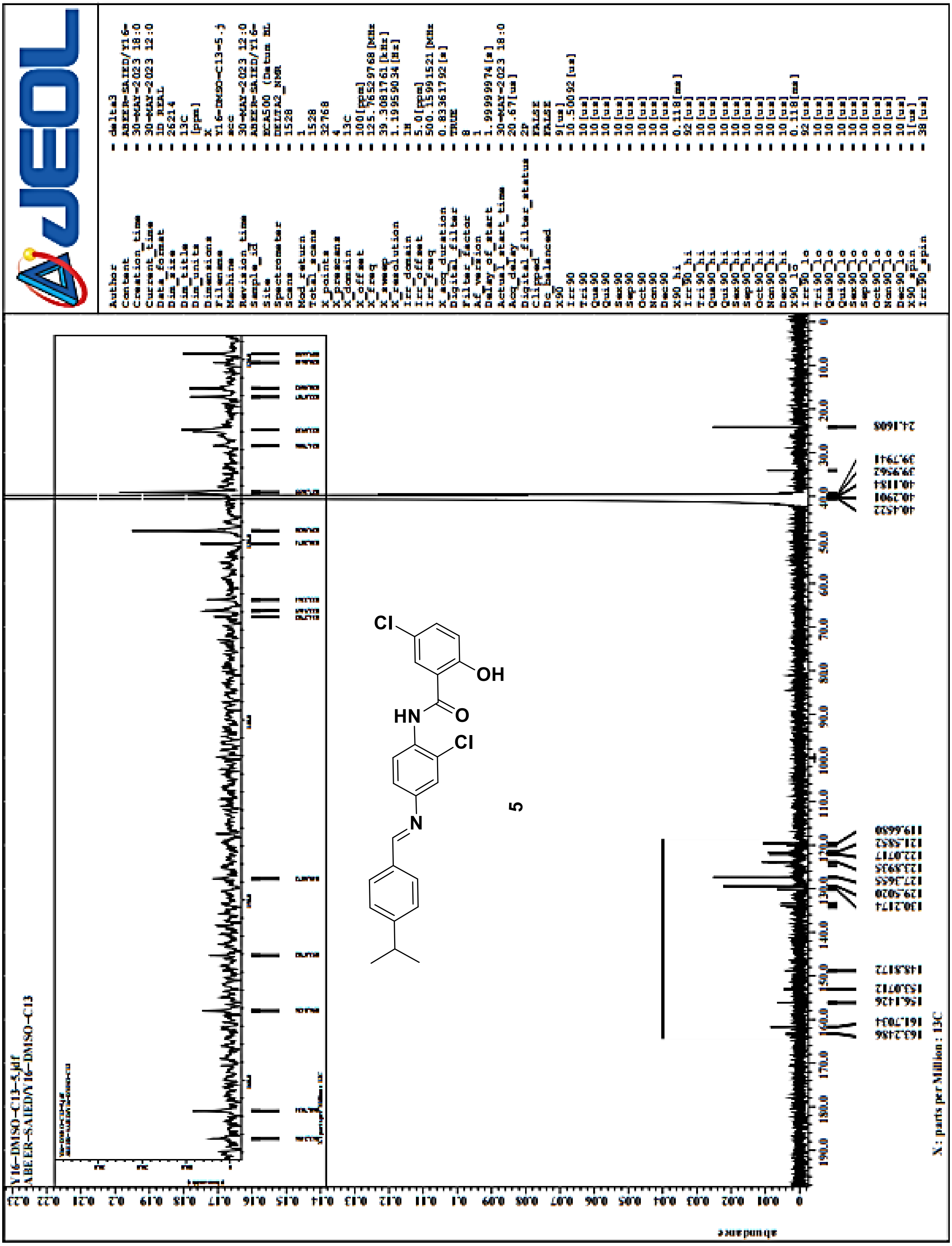

Appendix (4b): ^{13}C NMR spectrum of **compound 5**

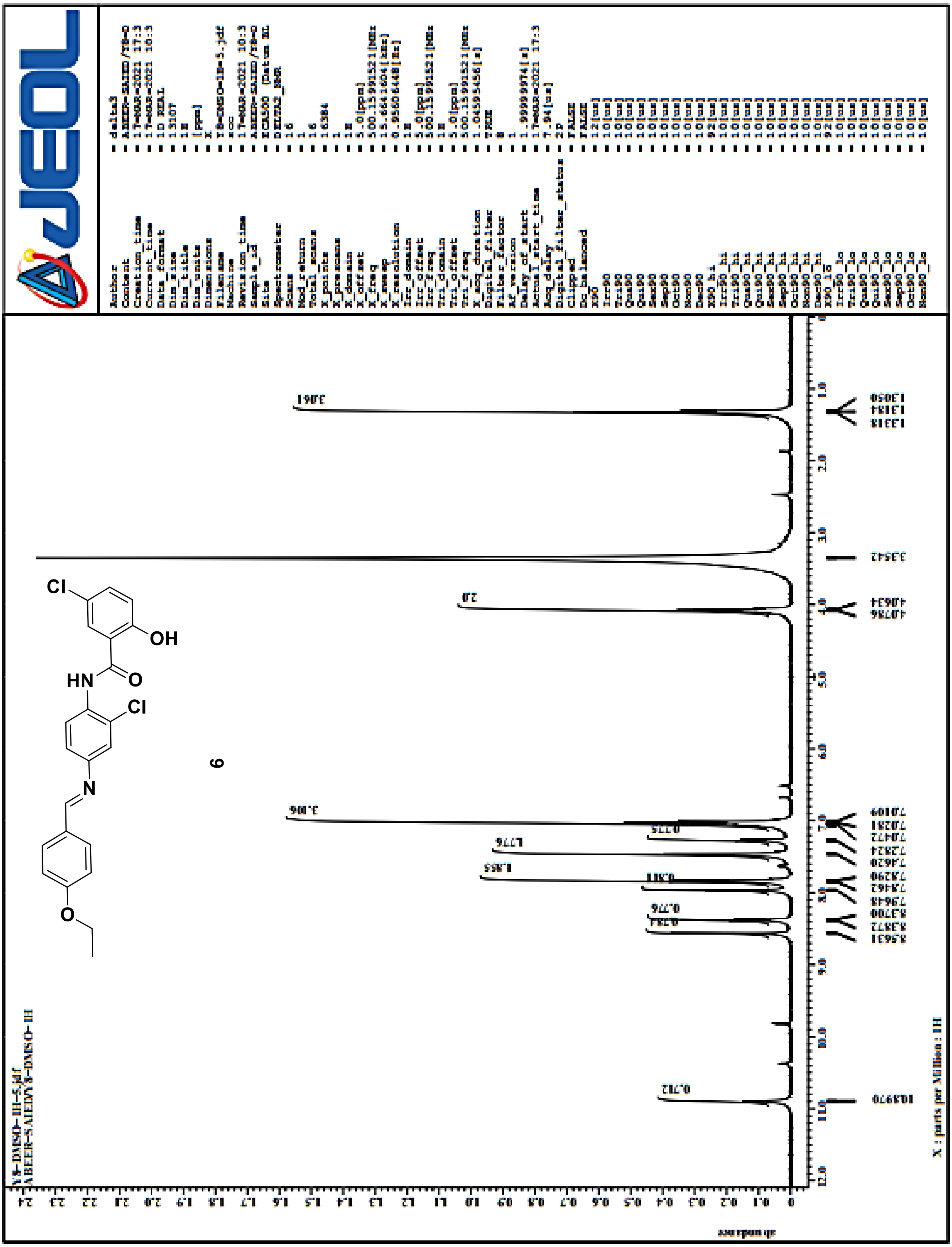

Appendix (5a): ^{1}H NMR spectrum of **compound 6**

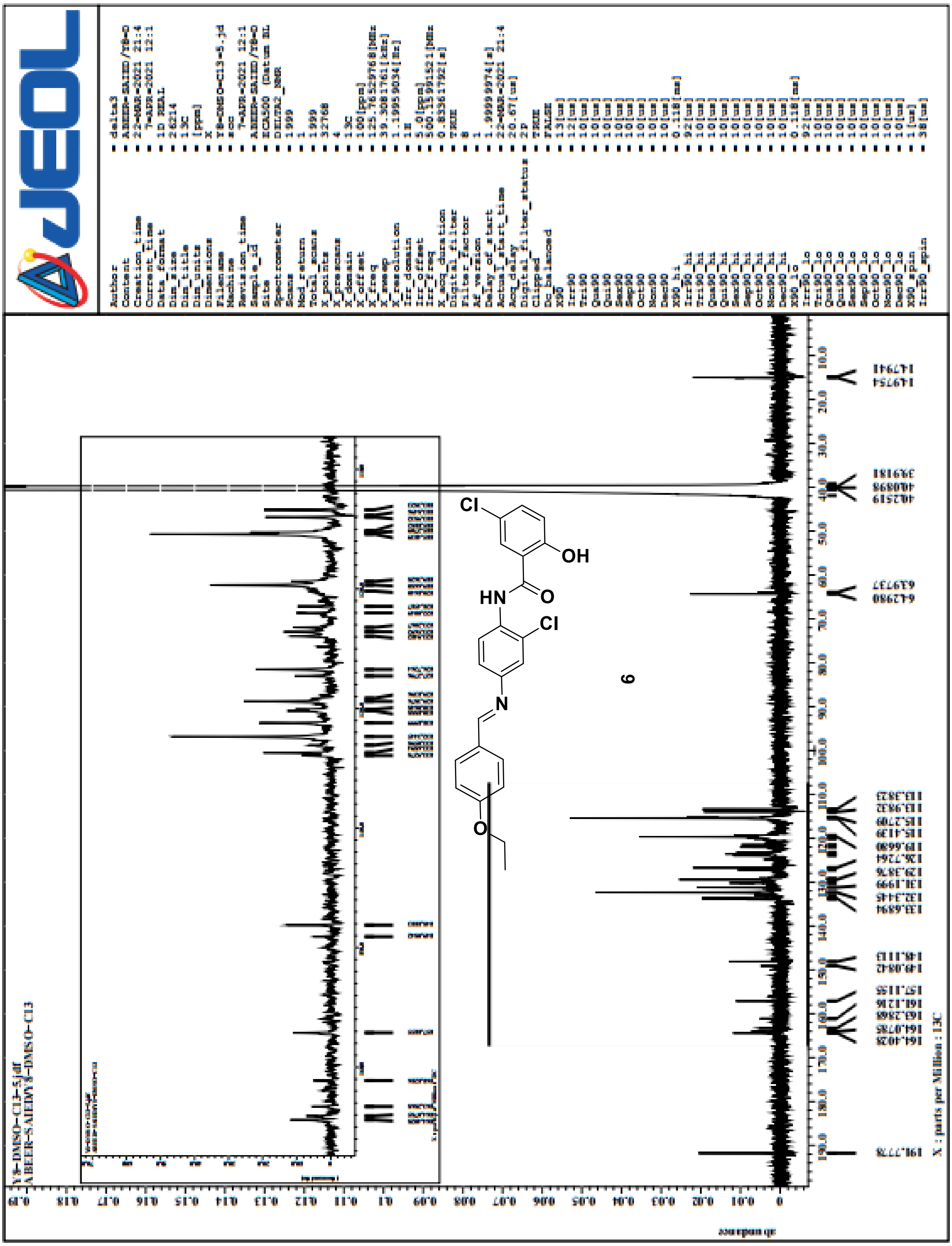

Appendix (5b): ^{13}C NMR spectrum of **compound 6**

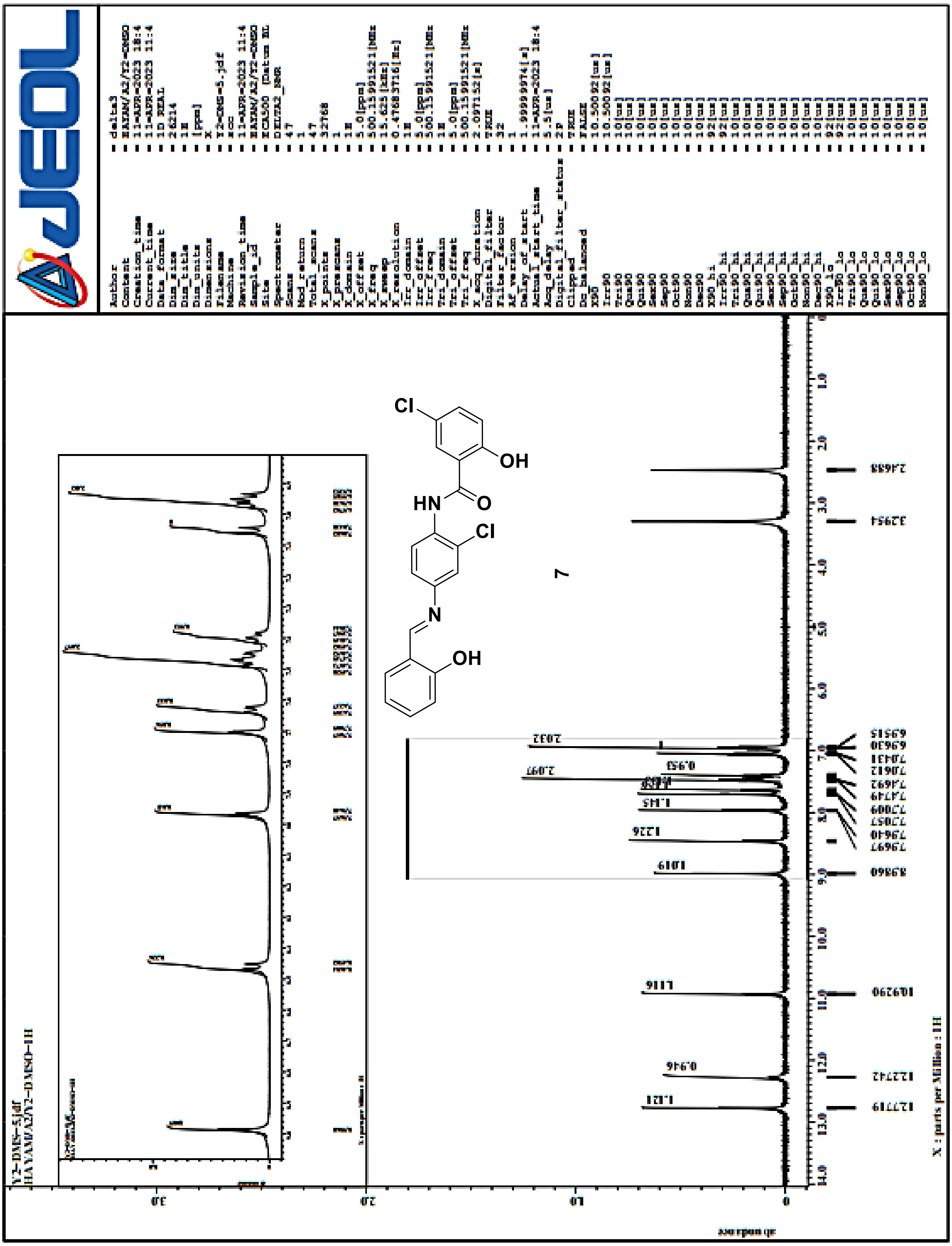

Appendix (6a): ^{1}H NMR spectrum of **compound 7**

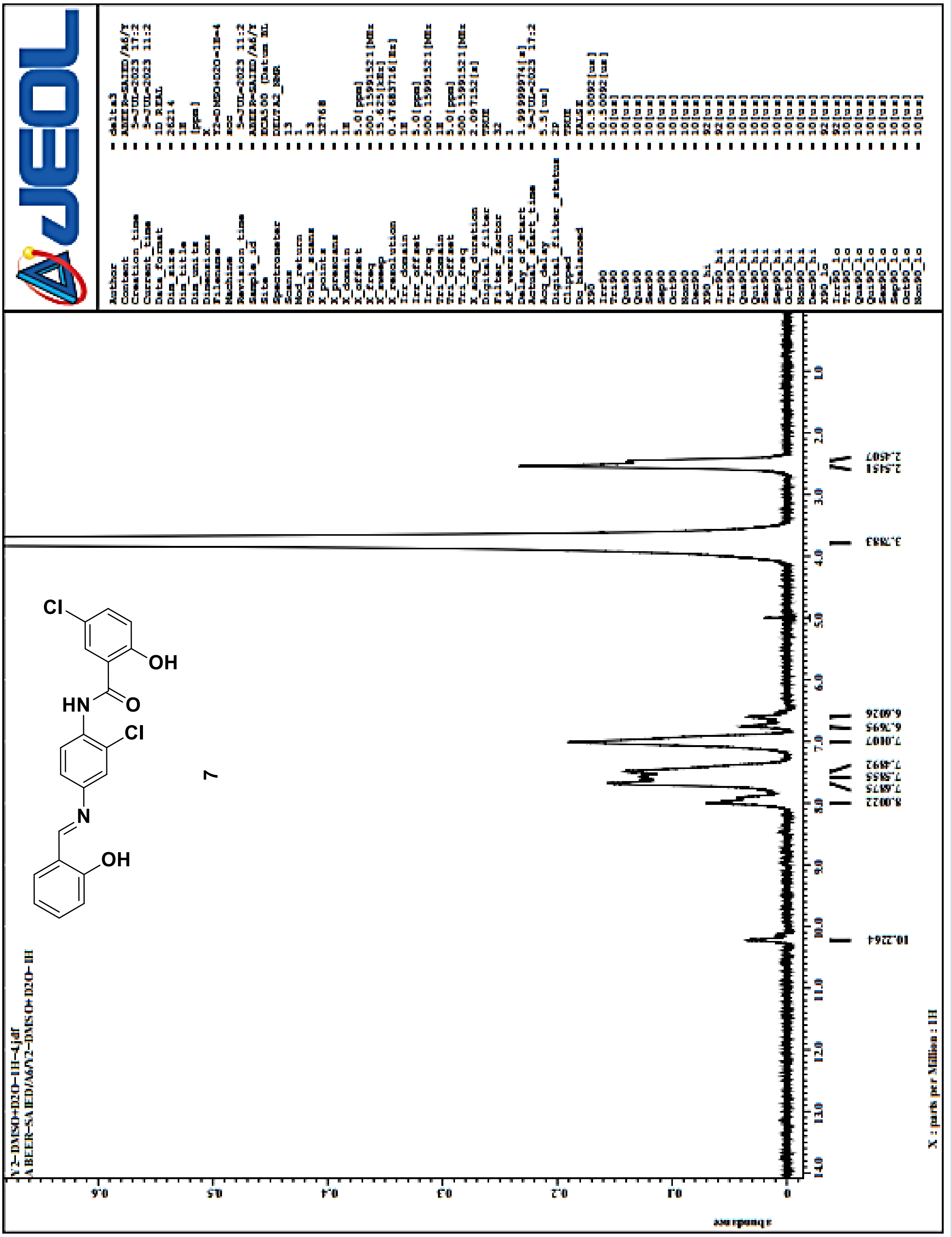

Appendix (6b): ^{1}H NMR spectrum of **compound 7 (D_2O)**

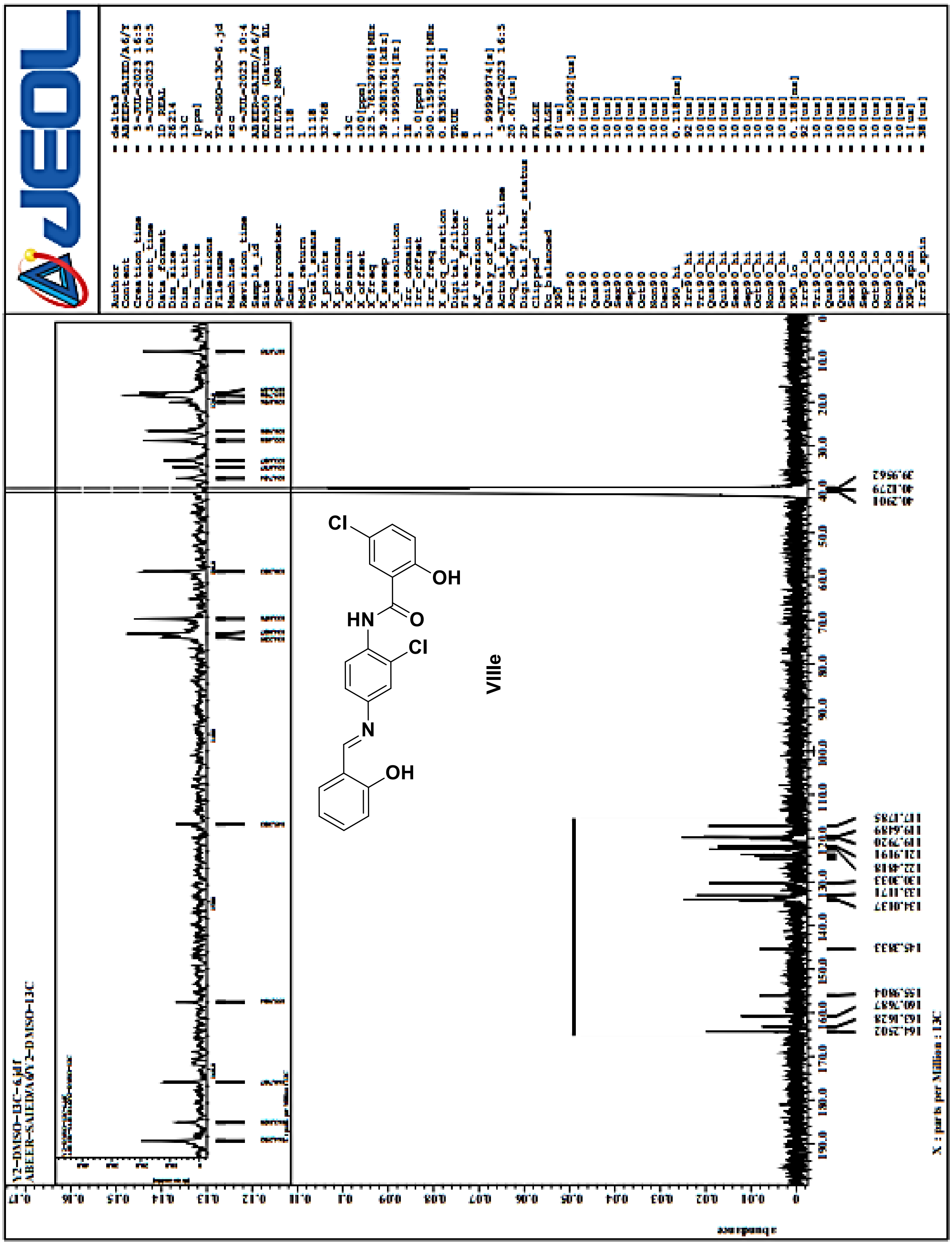
Cl
OH
HN
O
Cl
N
OH
VIIIe

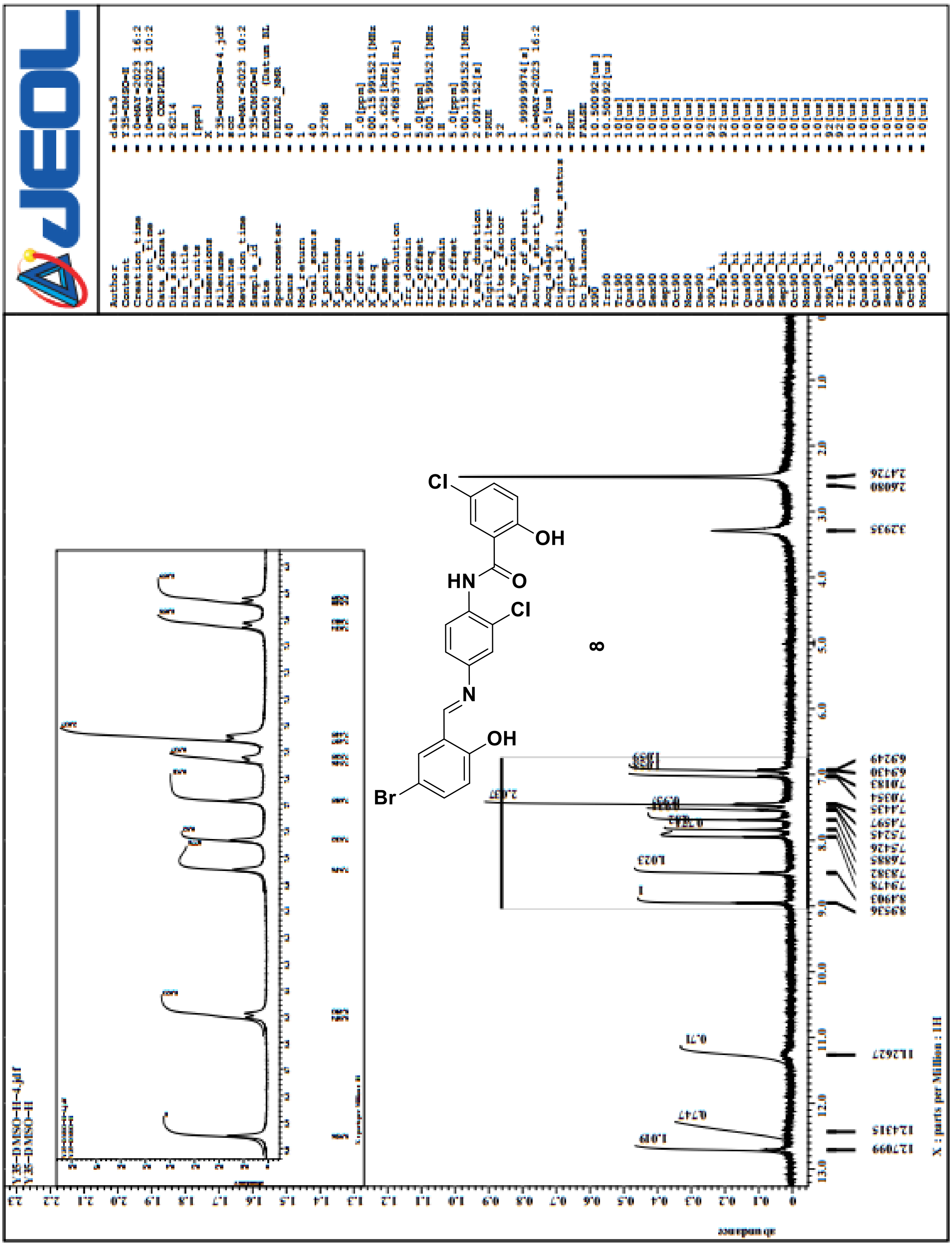

Appendix (7a): ^{1}H NMR spectrum of **compound 8**

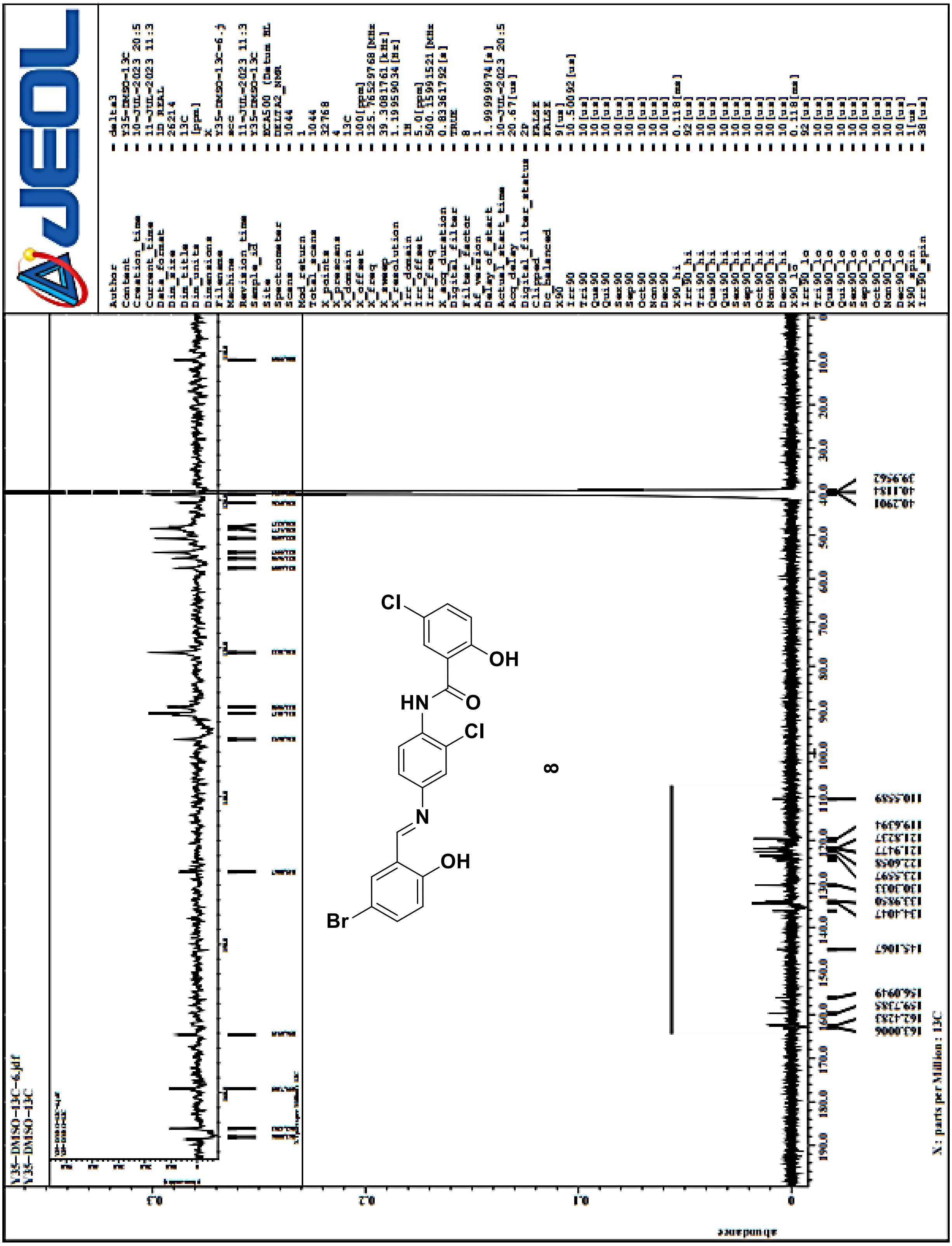

Appendix (7b): ^{13}C NMR spectrum of **compound 8**

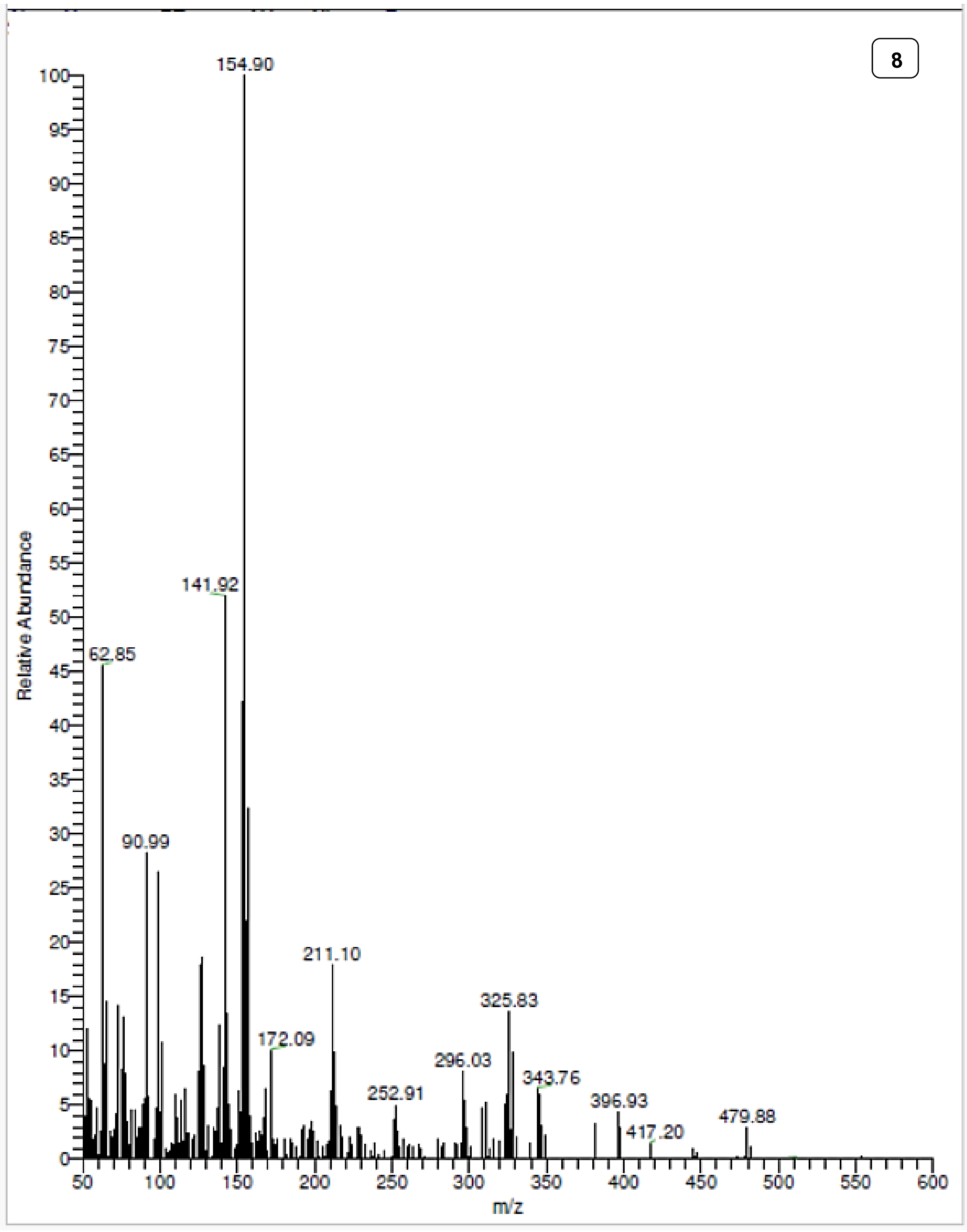

Appendix (7c): Mass spectrum of **compound 8**

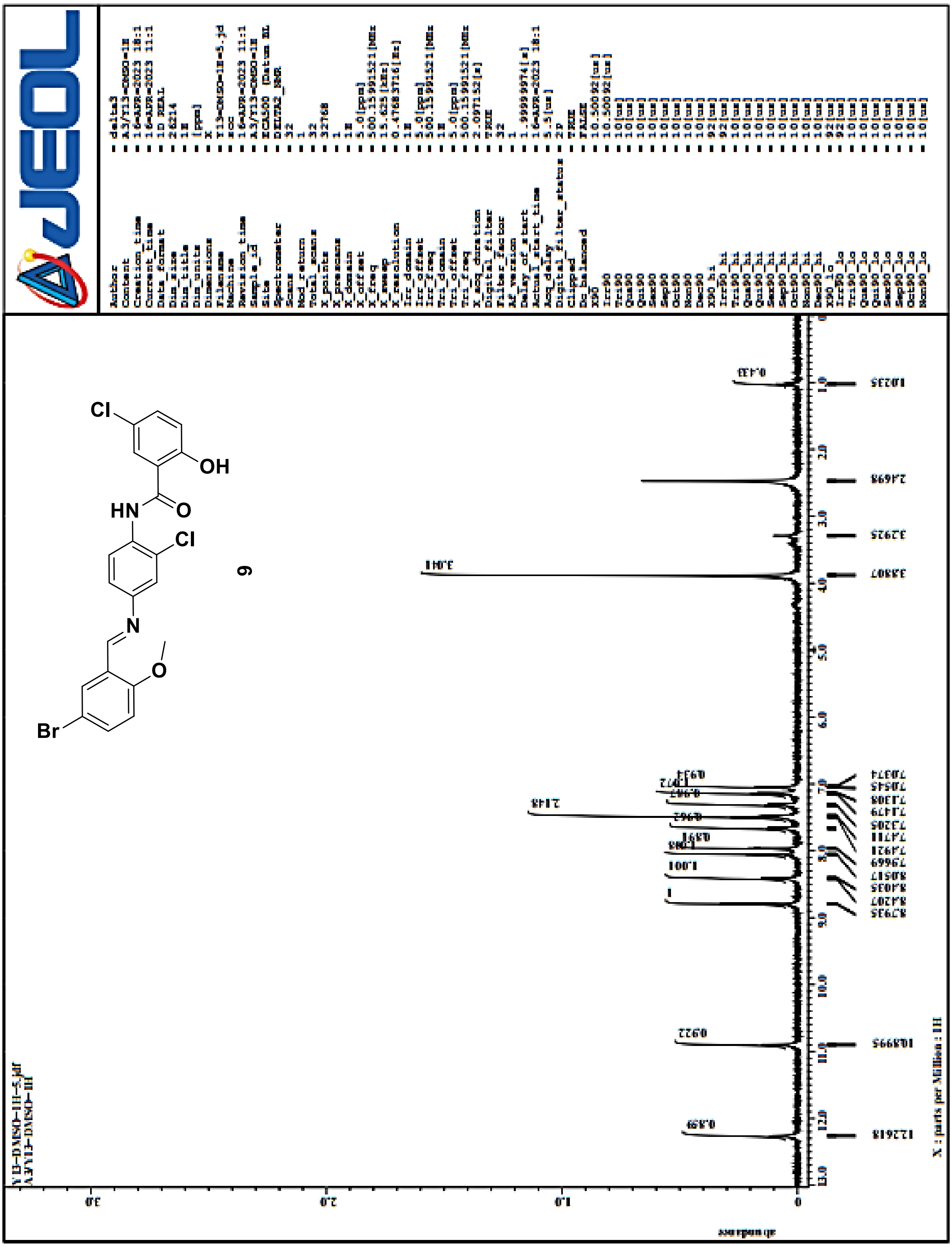

Appendix (8a): ^{1}H NMR spectrum of **compound 9**

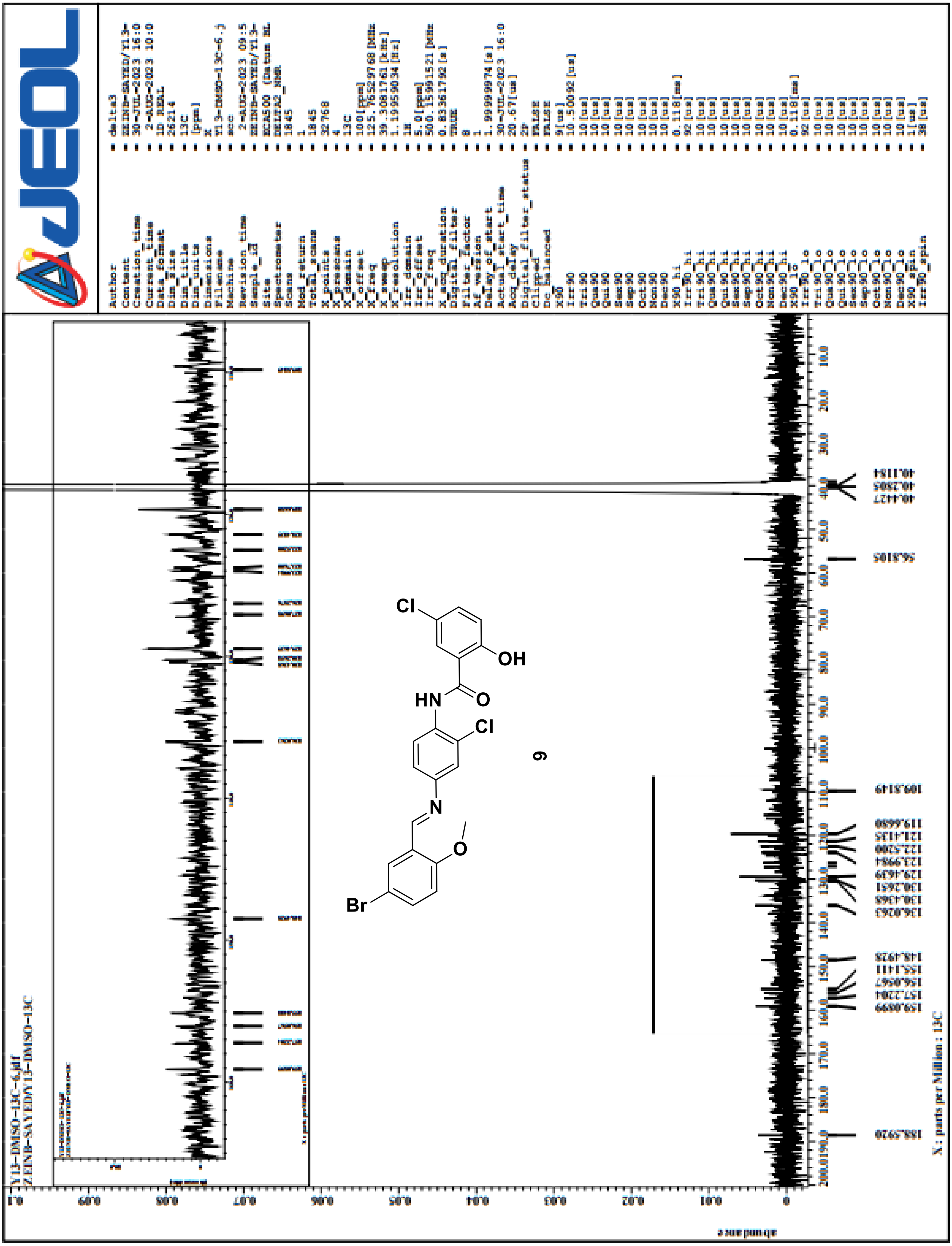

Appendix (8a): ^{13}C NMR spectrum of **compound 9**

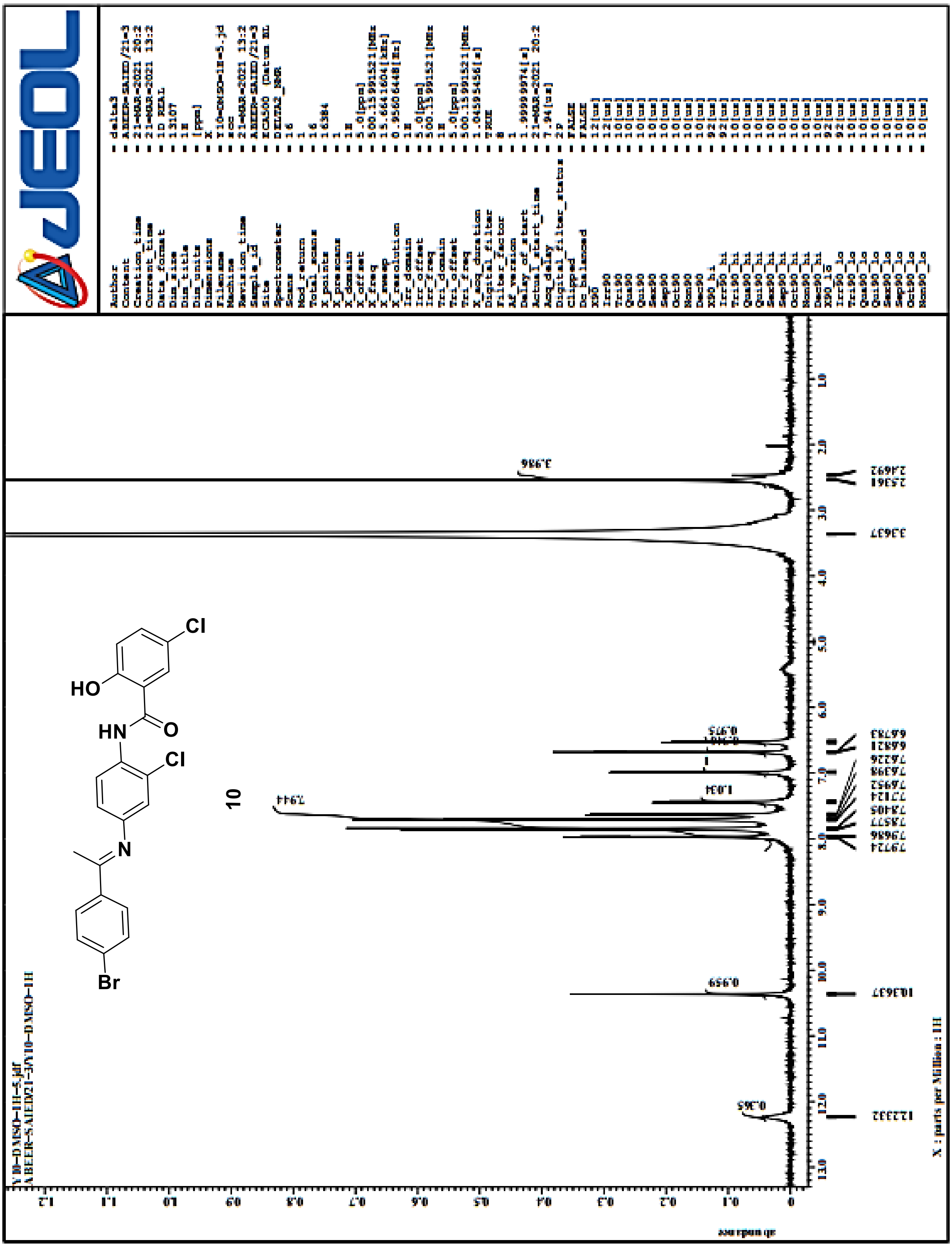

Appendix (9a): ^{1}H NMR spectrum of **compound 10**

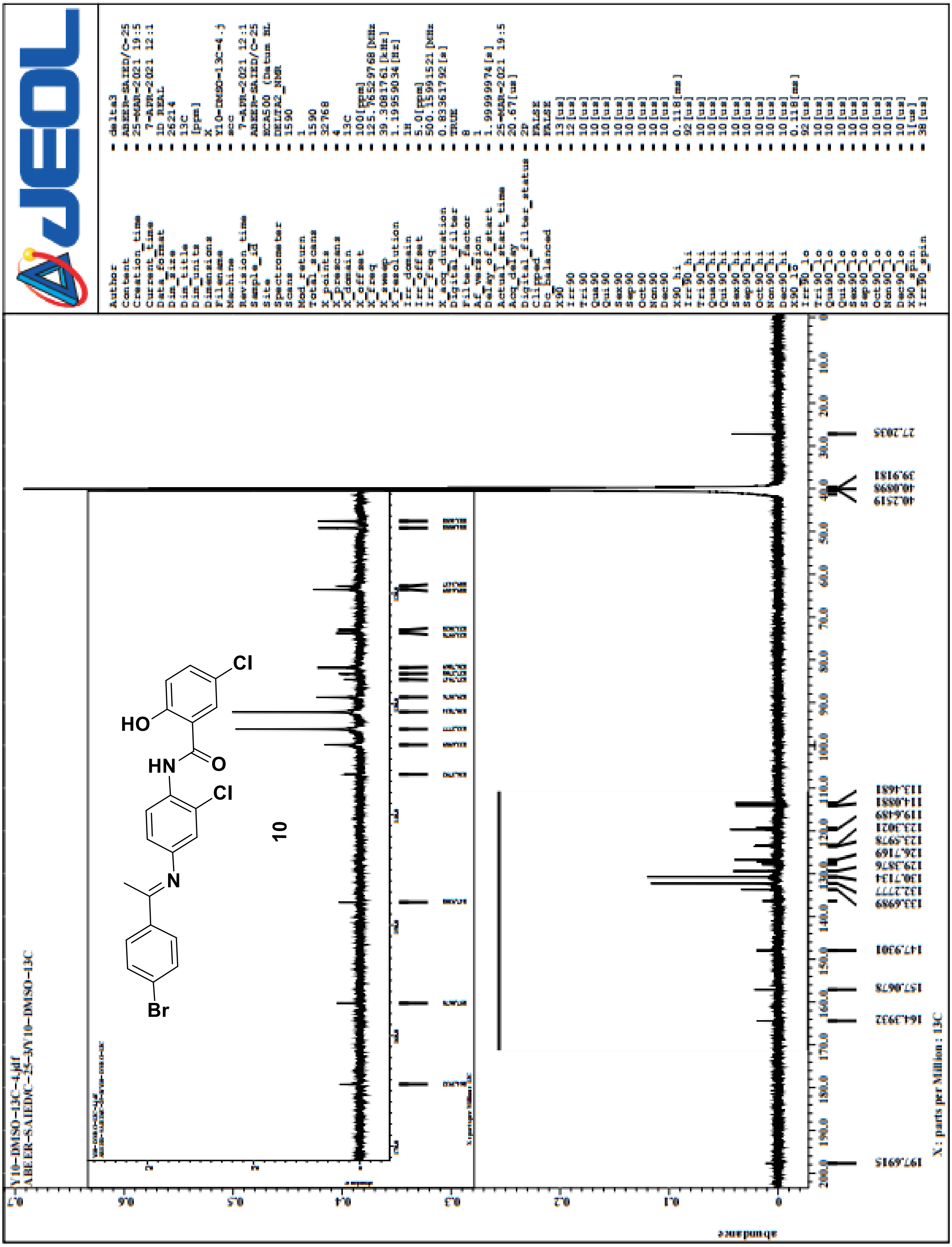

Appendix (9b): ^{13}C NMR spectrum of **compound 10**

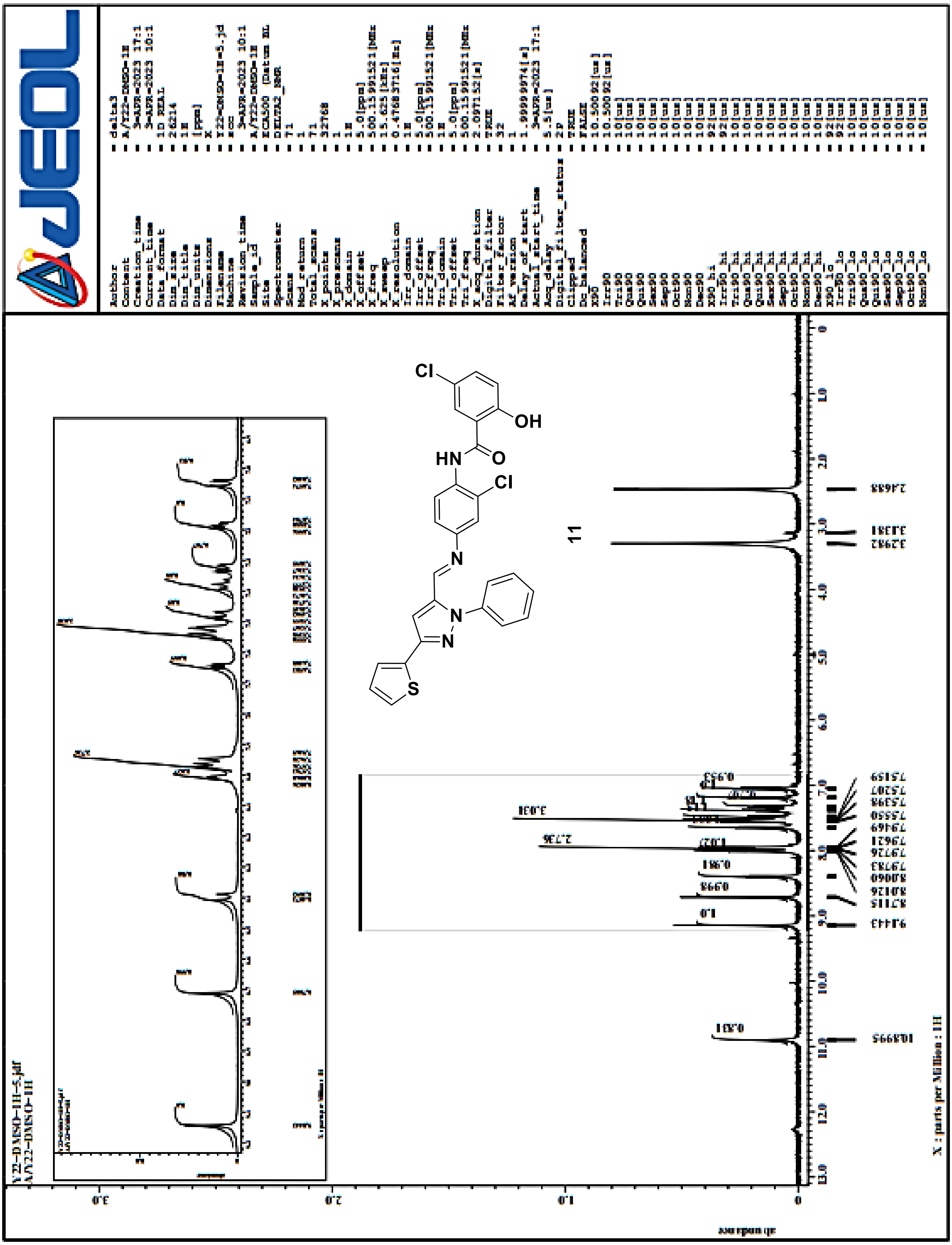

Appendix (10a): ^{1}H NMR spectrum of **compound 11**

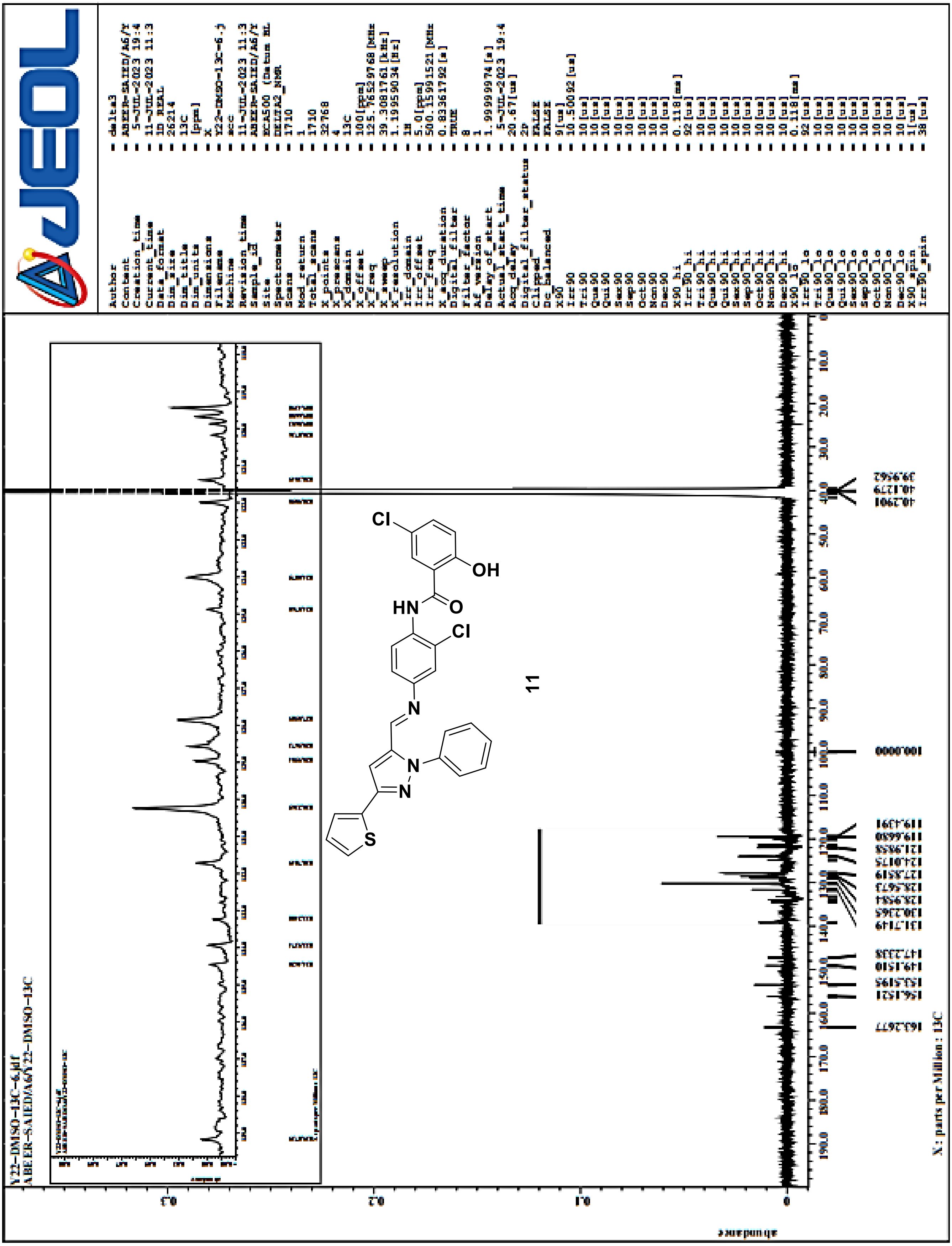

Appendix (10b): ^{13}C NMR spectrum of **compound 11**

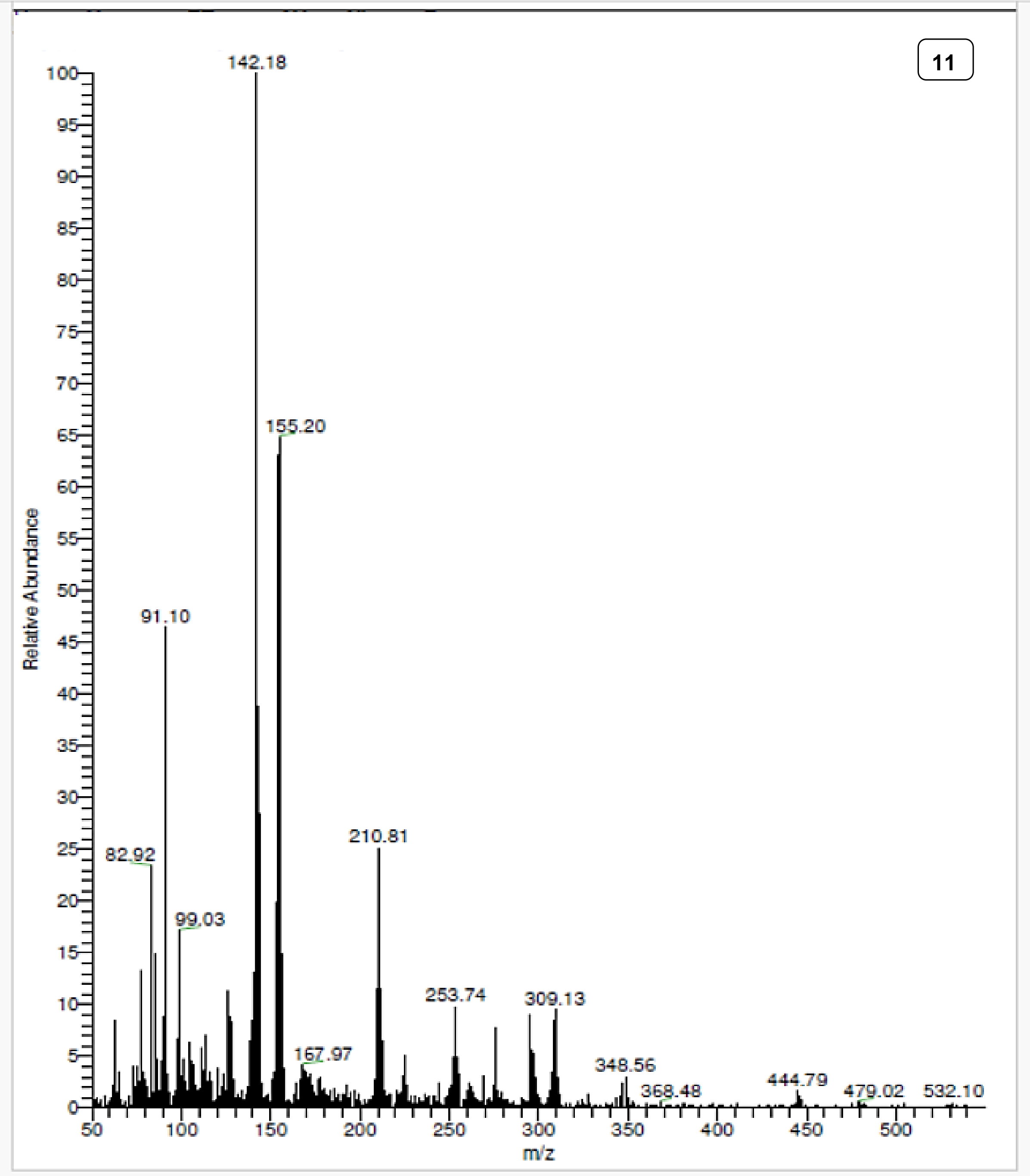
11
142.18
155.20
91.10
210.81
82.92
99.03
253.74
309.13
167.97
348.56
368.48
444.79
479.02
532.10
Relative Abundance
m/z

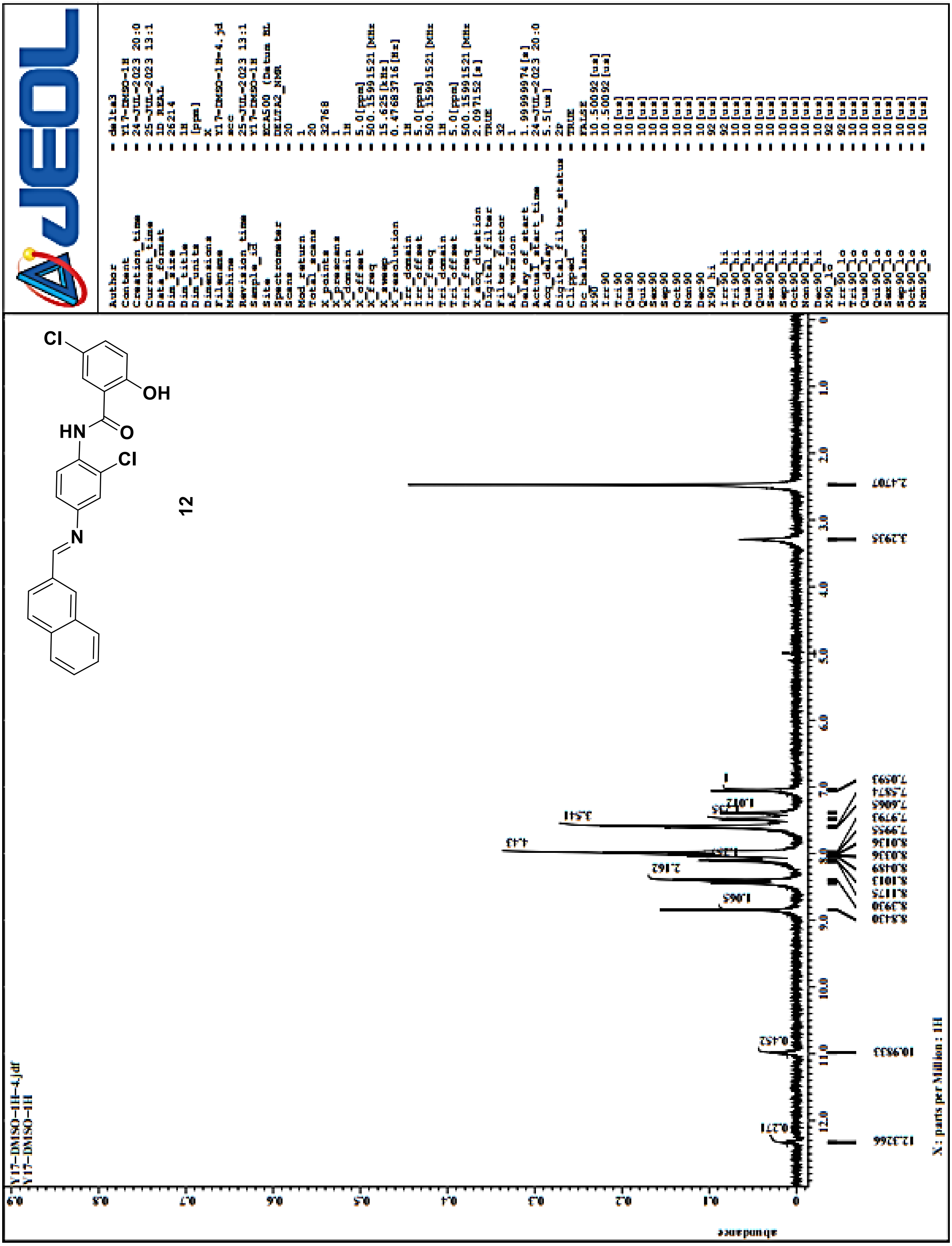

Appendix (11a): ^{1}H NMR spectrum of **compound 12**

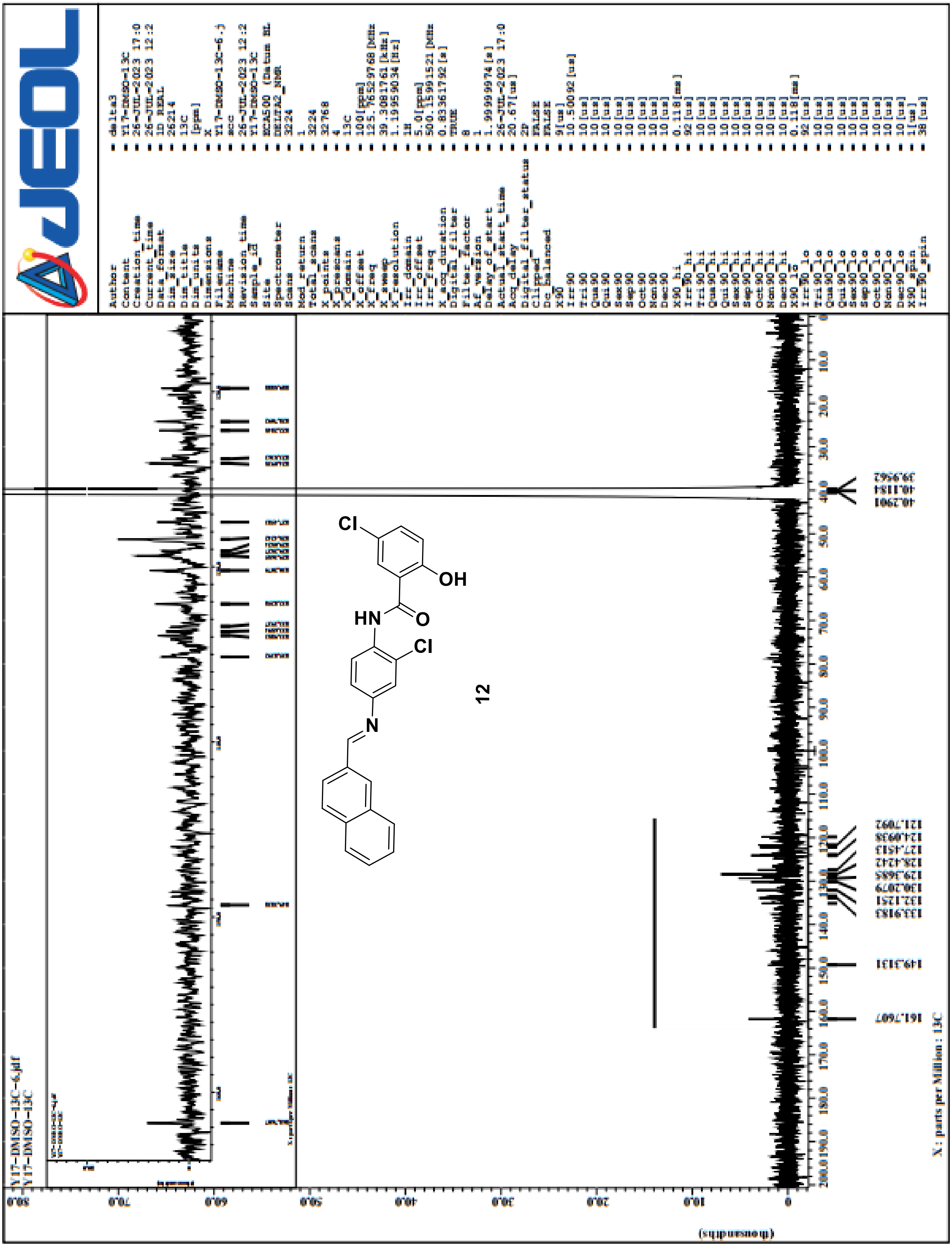

Appendix (11b): ^{13}C NMR spectrum of **compound 12**

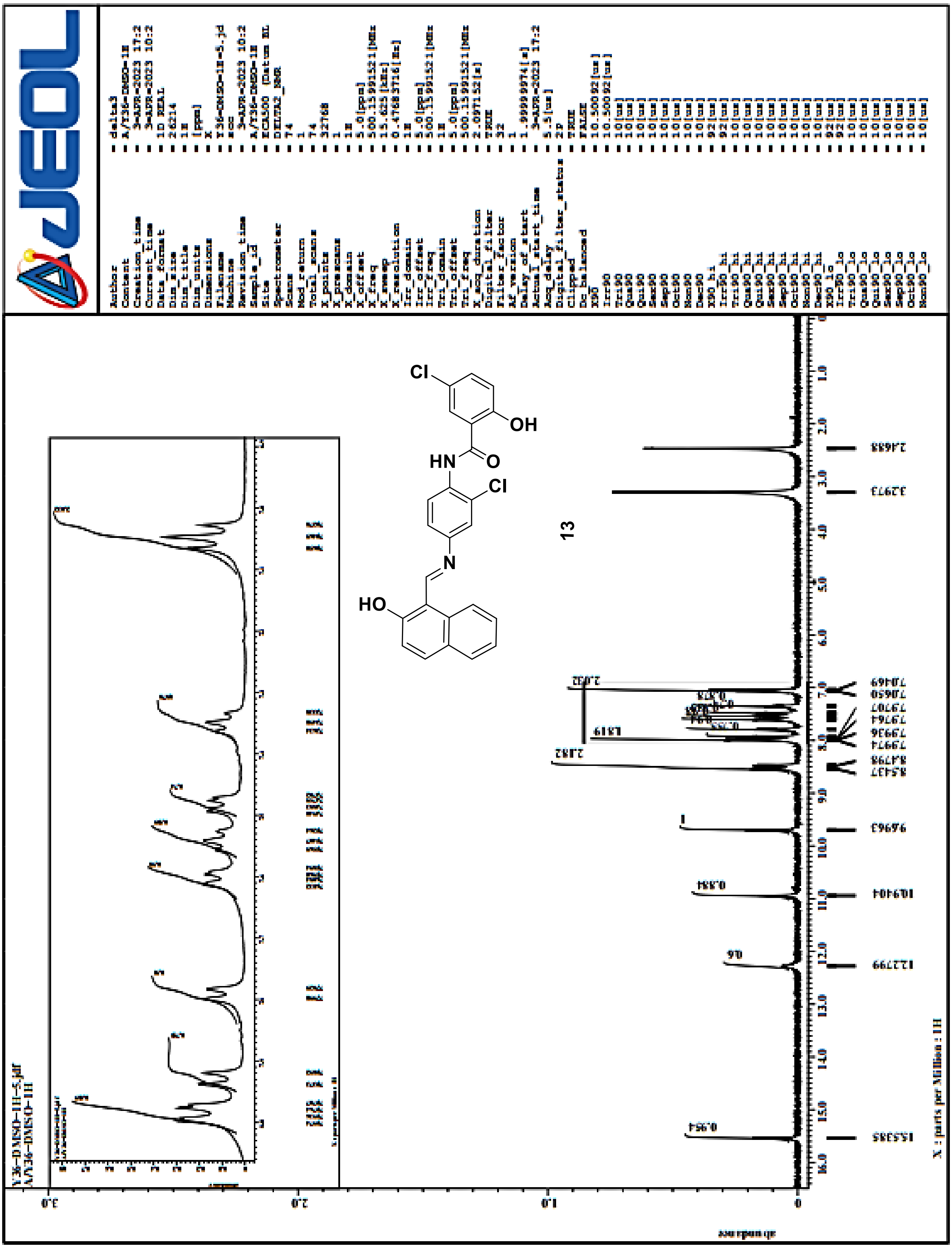

Appendix (12a): ^{1}H NMR spectrum of **compound 13**

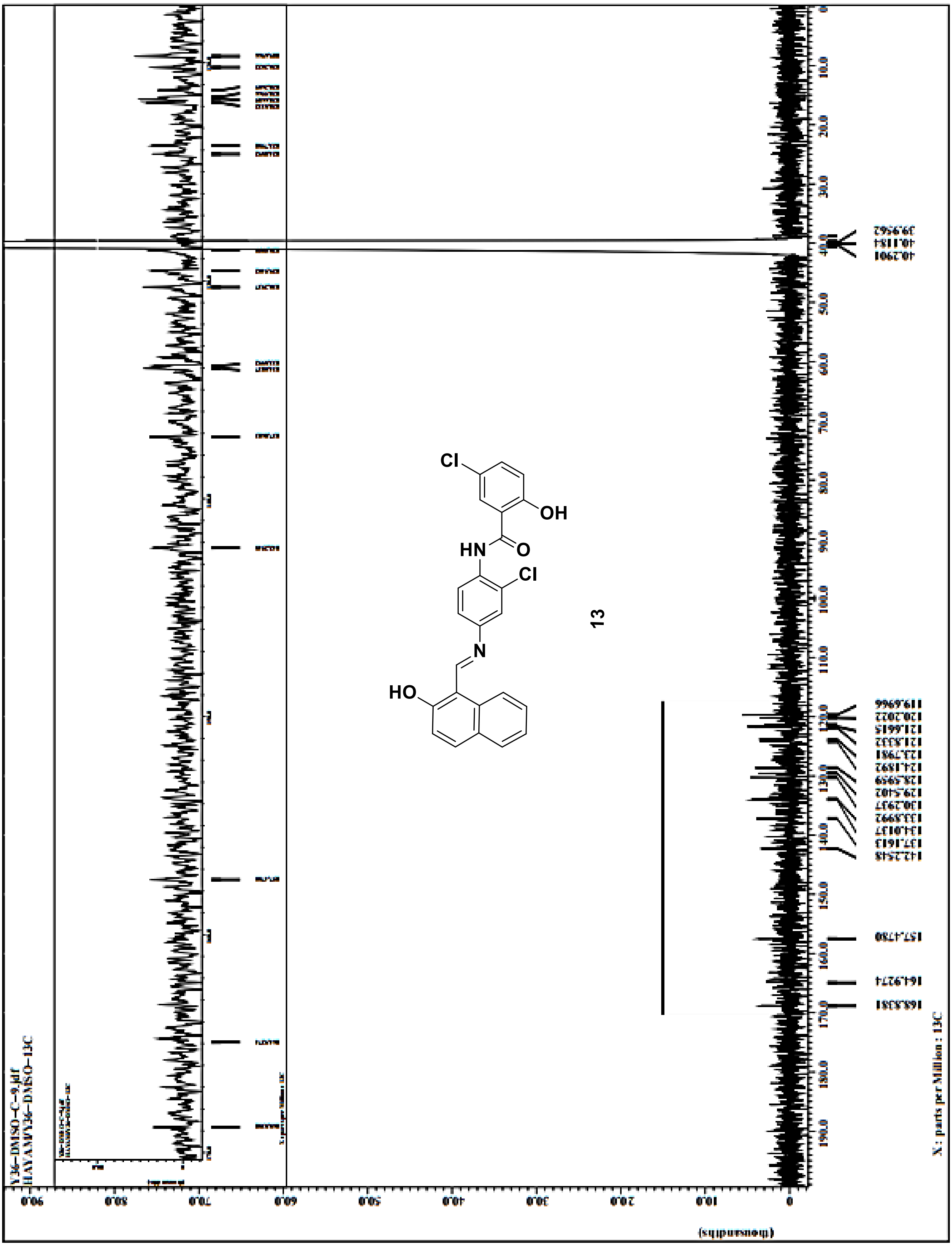

Appendix (12b): ^{13}C NMR spectrum of **compound 13**

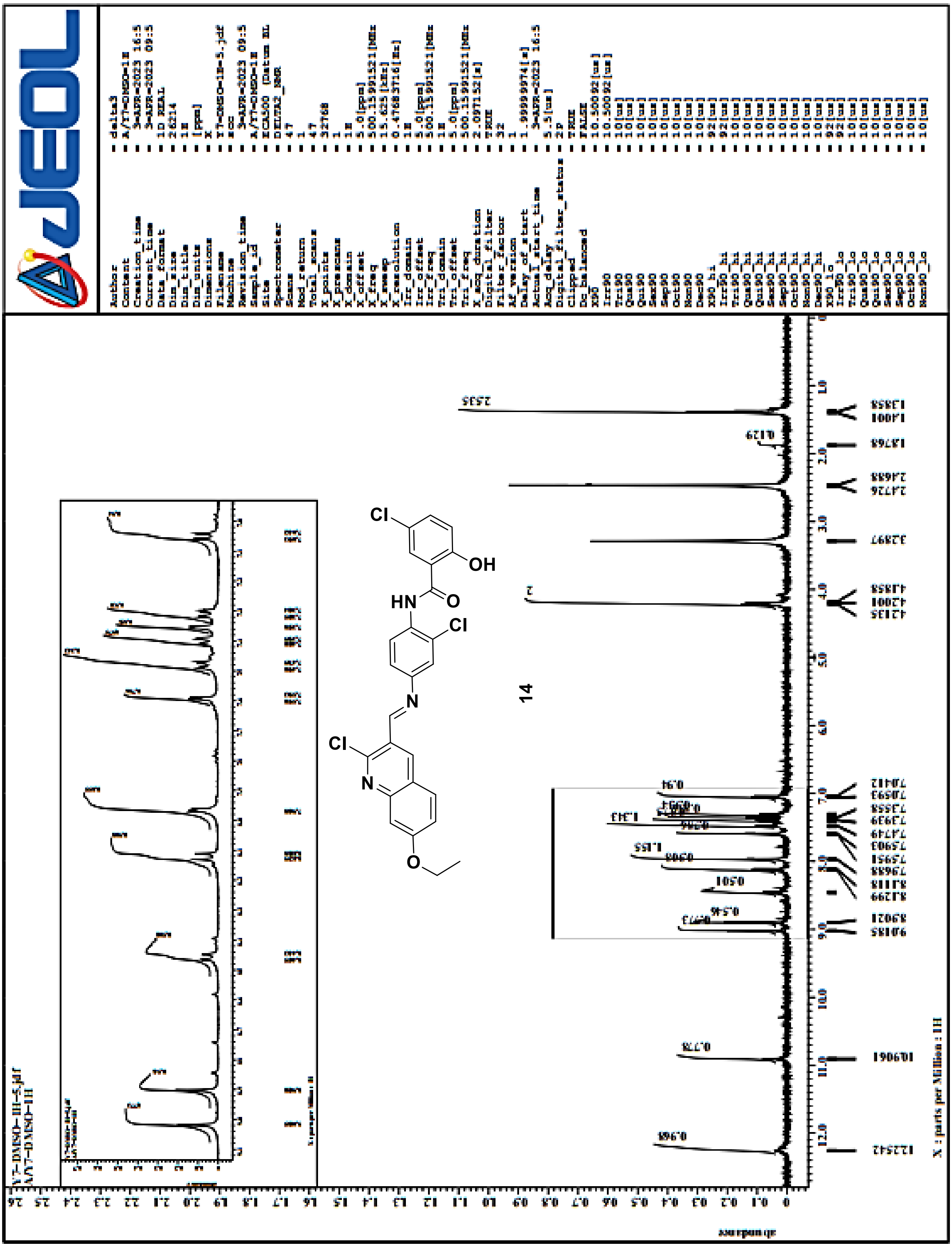

Appendix (13a): ^{1}H NMR spectrum of **compound 14**

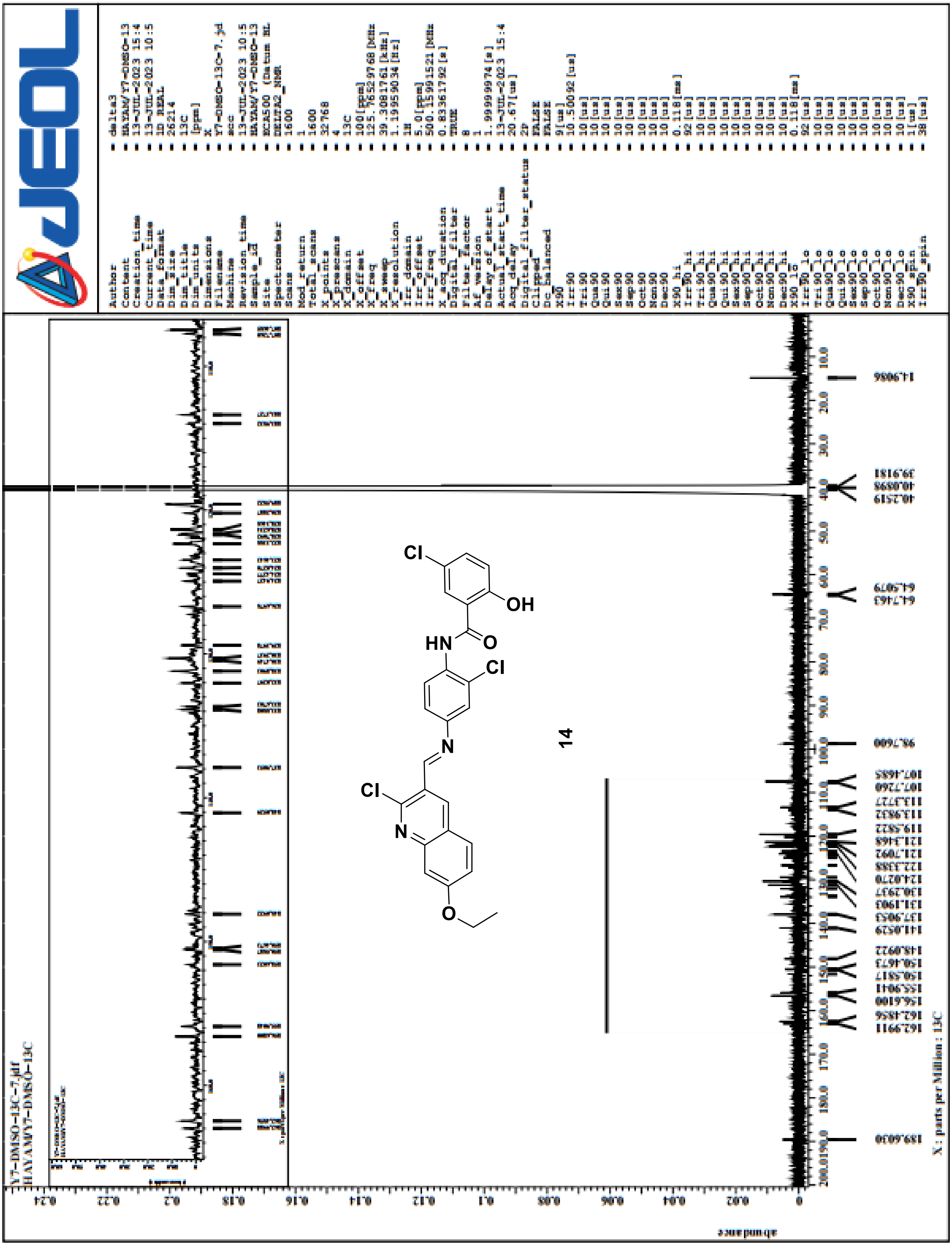

Appendix (13b): ^{13}C NMR spectrum of **compound 14**

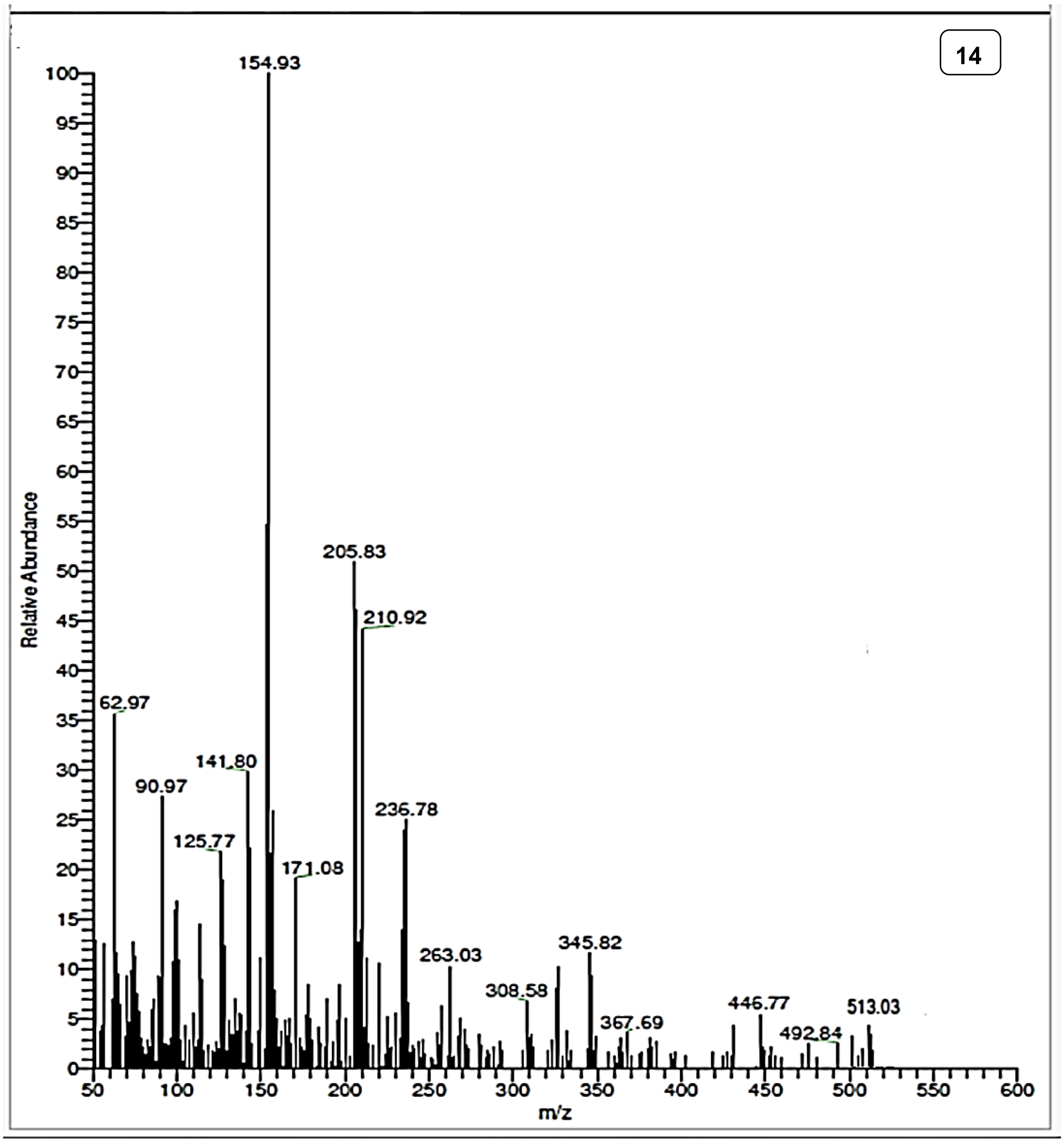

Appendix (13C): Mass spectrum of **compound 14**

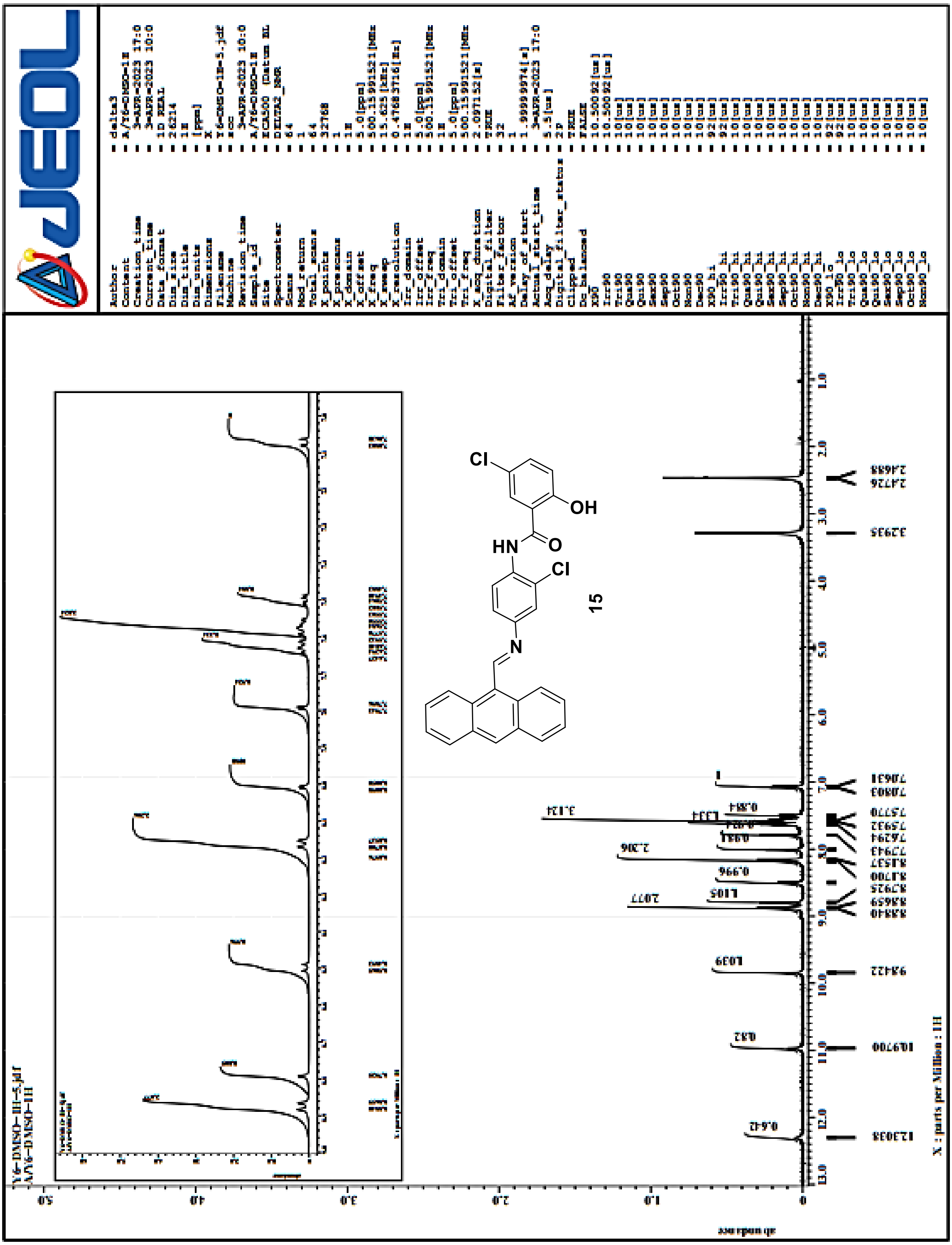

Appendix (14a): ^{1}H NMR spectrum of **compound 15**

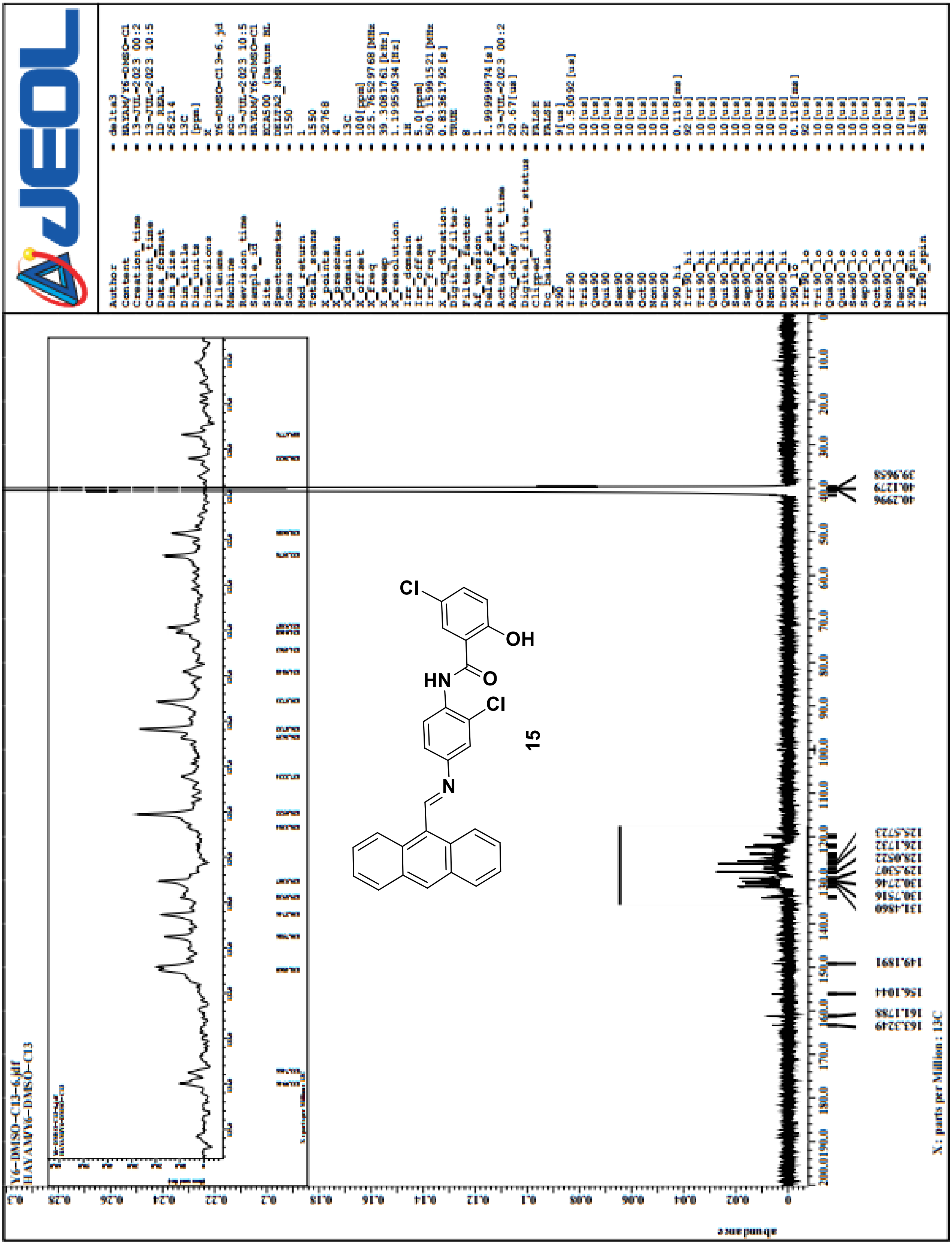

Appendix (14b): ^{13}C NMR spectrum of **compound 15**

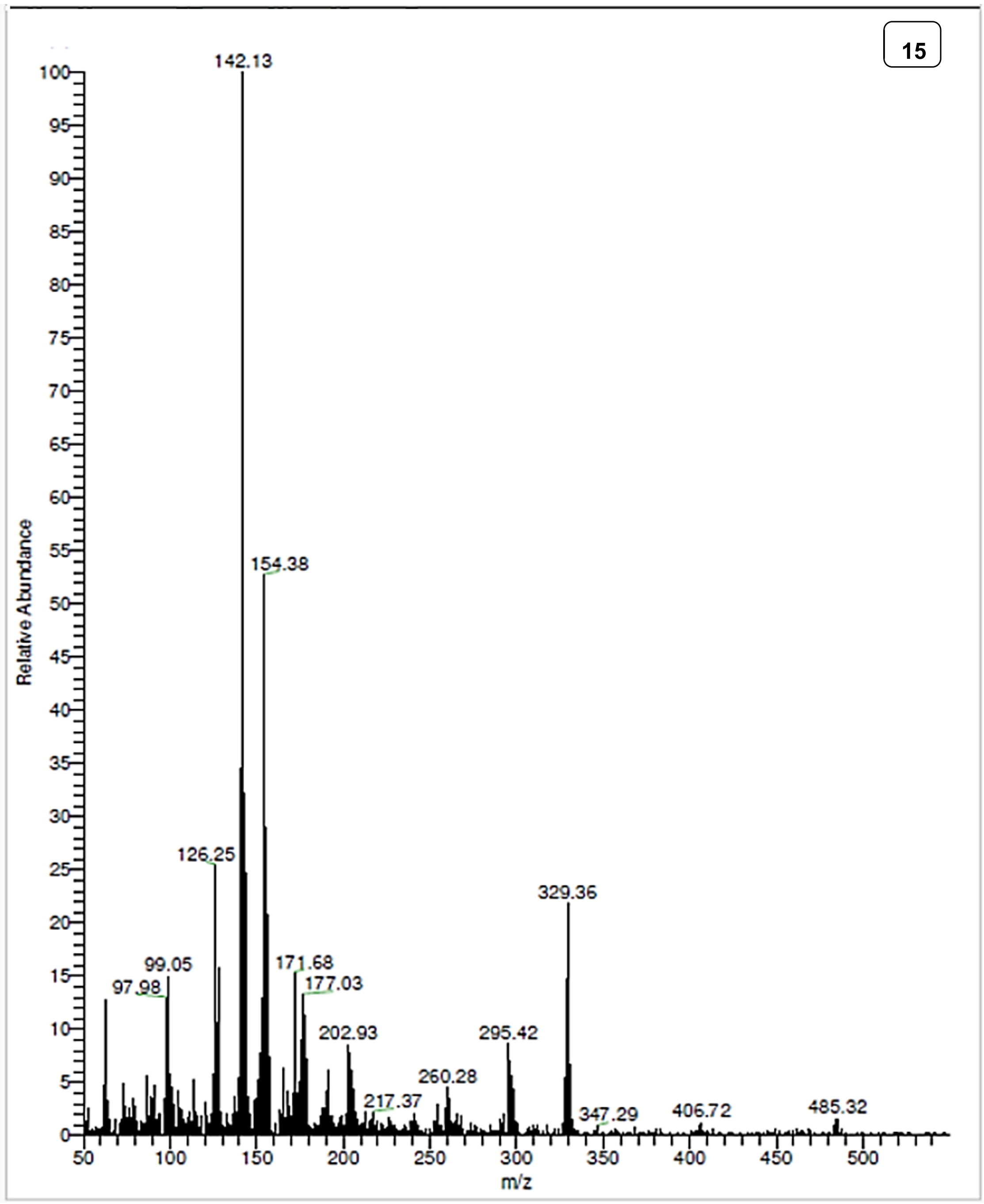

Appendix (14C): Mass spectrum of **compound 15**
