# Supplementary material for: Novel niclosamide-derived Schiff bases as a dual-targeted anticancer agents
Source: Sci Rep. 2026 Jan 13;16:1959. doi: 10.1038/s41598-025-33185-2 (PMC12804884; doi:10.1038/s41598-025-33185-2)
Supplement: Supplementary file 2 — Supplementary Material 2 [file 41598_2025_33185_MOESM2_ESM.docx]

**Supplementary material S2**

**Cytotoxic evaluation of the Selected Compounds IC_50_ µM**

Conc.

| **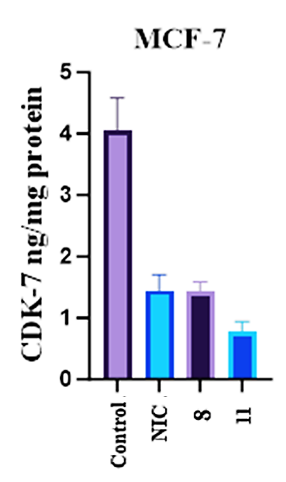** | **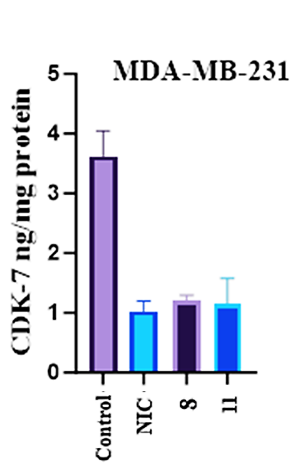** | **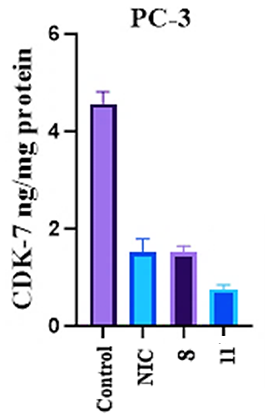** |
| --- | --- | --- |

Inhibition % of CDK7 on the three tested cell lines

Evaluation of JAK1 inhibition (IC_50_ uM) on the three tested cell lines
